# Exploring the Causal Association between Morning Diurnal Preference and Psychiatric Disorders: A Bidirectional Two-Sample Mendelian Randomization Analysis

**DOI:** 10.3390/life14101225

**Published:** 2024-09-25

**Authors:** Manman Chen, Din-Son Tan, Xijie Wang, Zichen Ye, Zhilan Xie, Daqian Zhang, Dandan Wu, Yuankai Zhao, Yimin Qu, Yu Jiang

**Affiliations:** 1 School of Population Medicine and Public Health, Chinese Academy of Medical Sciences and Peking Union Medical College, Beijing 100730, China; chenmm@pumc.edu.cn (M.C.); ye18700579760@163.com (Z.Y.); xzl_719@163.com (Z.X.); zhangdaqian@sph.pumc.edu.cn (D.Z.); wudandan@sph.pumc.edu.cn (D.W.); z4311652@163.com (Y.Z.); quyimin@sph.pumc.edu.cn (Y.Q.); 2 Vanke School of Public Health & Institute for Healthy China, Tsinghua University, Beijing 100084, China; hongchen_huajun@163.com; 3 School of Health Policy and Management, Chinese Academy of Medical Sciences and Peking Union Medical College, Beijing 100730, China

**Keywords:** chronotype, psychiatric disorders, Mendelian randomization, single-nucleotide polymorphisms

## Abstract

Background: The causal connection between morning diurnal preference and psychiatric disorders remains enigmatic. Using bidirectional two-sample Mendelian randomization (MR), we aim to explore the potential causal associations between morning diurnal preference and seven prominent psychiatric disorders. Methods: MR is a genetic epidemiological method that leverages genetic variants as instrumental variables to infer causal associations between exposures and outcomes. We obtained morning diurnal preference data from genome-wide association study (GWAS) datasets and identified 252,287 individuals as morning people. Psychiatric disorder data were sourced from the FinnGen consortium R9 dataset. Our primary analysis used the inverse-variance weighted (IVW) approach to evaluate the overall causal effect by combining the estimates from each genetic variant. Addition analyses, including weighted median, MR-Egger regression, weighted mode, and simple mode techniques were conducted to ensure robustness. Results: Being a morning person is related to reduced odds of multiple psychiatric disorders, including depression or dysthymia (OR: 0.93, 95% CI: 0.88, 0.999), anxiety disorders (OR: 0.90, 95% CI: 0.84, 0.96), self-harming behaviors (OR: 0.87, 95% CI: 0.76, 0.99), substance-use disorders (OR: 0.81, 95% CI: 0.71, 0.93), alcohol dependence (OR: 0.82, 95% CI: 0.73, 0.92), alcohol use disorders (OR: 0.85, 95% CI: 0.76, 0.94), acute alcohol intoxication (OR: 0.86, 95% CI: 0.76, 0.96), schizophrenia (OR: 0.77, 95% CI: 0.65, 0.92), and schizophrenia or delusion (OR: 0.80, 95% CI: 0.70, 0.92). Alcohol dependence (OR: 0.97, 95% CI: 0.94, 0.999) and alcohol use disorders (OR: 0.96, 95% CI: 0.94, 0.99) were also related to a lower morning diurnal preference. Conclusions: Our study provides evidence that being a morning person is a protective factor for various psychiatric disorders from a genetic perspective. The results provide insights for potential targeted interventions to improve mental wellbeing.

## 1. Introduction

Psychiatric disorders have shown no evidence of reduction since 1990, and they are now one of the leading causes of disease burden globally [1]. According to the latest Global Burden of Diseases (GBD) study, mental disorders contribute to disability-adjusted life years (DALYs) across all age groups [2]. In 2019, an estimate of 418 million DALYs (16% of global total) could be attributable to psychiatric disorders, resulting in an economic loss estimated at USD 5 trillion [3]. Furthermore, in recent years, the situation has potentially worsened. The emergence of the COVID-19 pandemic has led to unfavorable lifestyles, such as increased screen time and reduced physical activity, which can disturb normal circadian rhythms [4]. Moreover, these circadian rhythm disruptions, coupled with the social isolation and stress induced by the COVID-19 pandemic, have further exacerbated psychiatric disorders [5]. This confluence of factors underscores the urgent need for a comprehensive and effective approach to address the global mental health crisis.

Altered circadian rhythms are commonly observed in individuals with psychiatric disorders [6]. Observational studies have reported that individuals an evening (“owl-like”) diurnal preference tend to have higher risks of psychiatric disorders, while those with a morning diurnal preference are often associated with lowered disease risks [7,8,9]. Nevertheless, evidence suggests that sleep-wake cycles could be adaptable to environmental factors, regardless of genetic predispositions for circadian preferences. For instance, individuals living in Middle and South America tend to adapt quickly due to the cultural practice of taking an afternoon nap (“siesta”) [10,11,12]. This adaptability highlights the complexity of the relationship between environment, diurnal preference, and mental health.

A recent genome-wide association study (GWAS) identified 351 genetic variants associated with diurnal preference, with an estimated heritability of 13.7 [13]. The findings utilized genetic techniques such as Mendelian randomization (MR) to investigate potential causal associations between diurnal preference and psychiatric disorders. Although several studies reported that morning preference was associated with lowered risks of schizophrenia [13], depression, and poor mental wellbeing [14], most of the previous studies have been limited to a narrow range of psychiatric disorders. Significant gaps remain in understanding the association between diurnal preference and other psychiatric conditions.

Given the high overlapping nature of various features across multiple psychiatric disorders, diurnal preference might be a common psychopathology factor that mediates phenotypic presentations across psychiatric disorders [15]. However, further evidence and rigorous investigations are needed to validate this hypothesis. In the current study, we performed a bidirectional two-sample MR analysis to assess the potential causal relationship between morning diurnal preference and seven major categories of psychiatric disorders [16], namely mood disorders, anxiety, substance-use disorders, impulse-control disorders, eating disorders, schizophrenia and other psychotic disorders, and dementia. Our findings may provide evidence to guide the development of targeted interventions and therapies aimed at improving mental health outcomes for individuals with varying diurnal preferences across these psychiatric conditions.

## 2. Materials and Methods

### 2.1. Study Design

**Figure 1:**
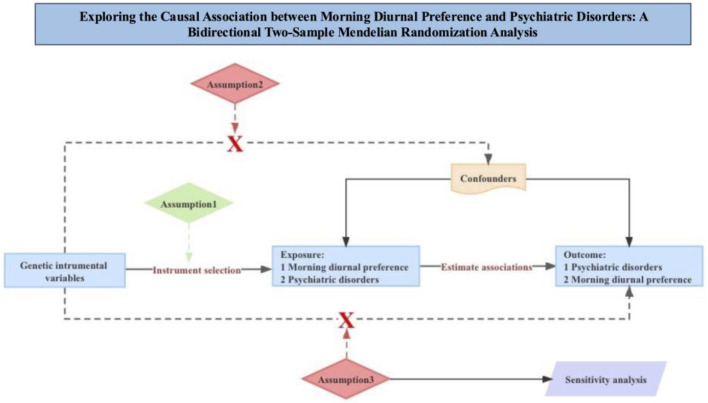
The fundamentals of Mendelian randomization.

Our bidirectional two-sample MR study was carried out within the framework depicted in Figure 1. We utilized genetic variants to investigate the potential causal relationship between morning diurnal preference and psychiatric disorders, as well as to explore reverse causation separately. To ensure the robustness of our results, we imposed three essential assumptions on the effective genetic instrumental variables: (1) the instrumental variables were predictive of the exposure; (2) the instrumental variables were not related to any confounding factors that were associated with both the exposure and the outcome; and (3) the instrumental variables influenced outcome only through the exposure. In each direction of inference, our analysis comprised three main steps: selecting appropriate instrumental variables for the exposure of interest, applying multiple MR methods, and conducting sensitivity analyses.

Note: Mendelian randomization requires valid genetic instrumental variants satisfying three assumptions: Assumption 1—genetic variants are predictive of the exposure; Assumption 2—genetic variants are independent of confounders; Assumption 3—genetic variants affect the association of the outcome solely through the exposure rather than via other pathways.

### 2.2. Data Sources

#### 2.2.1. Genetic Instrumental Variables

**Figure 2:**
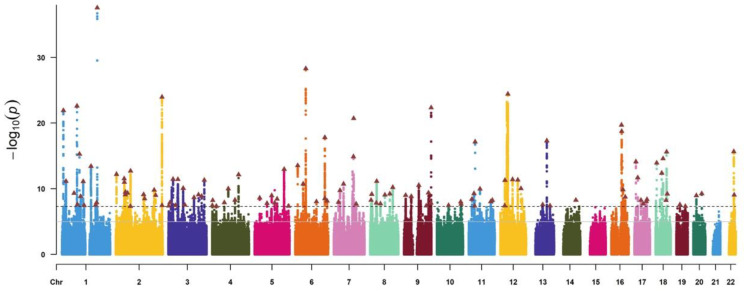
Manhattan plot of the morning diurnal preference meta-analysis GWAS.

Our MR study relied on publicly available studies and shared datasets. We initially identified single-nucleotide polymorphisms (SNPs) associated with morning diurnal preference and psychiatric disorders at genome-wide significance (*p* < 5 × 10^−8^) from the full GWAS summary data. Subsequently, we applied the PLINK method to clump the SNPs, adhering to criteria of linkage disequilibrium (LD) R^2^ < 0.001 and genomic windows exceeding 10,000 kb to ensure the independence of the genetic instrumental variables. In cases where SNPs exhibited LD, we retained the one with the lowest *p* value (Figure 2 for the Manhattan plot illustrating morning diurnal preference).

Note: chr, chromosome: The black dotted line indicates the typical genome-wide significance threshold of *p* = 5 × 10^−8^, and the solid gray line marks the threshold of *p* = 5 × 10^−5^ identified through permutation testing. Lead variants are annotated with a triangle.

It is important to note that certain SNPs may exert pleiotropic effects, potentially introducing bias in causal interpretations. To mitigate the risk of potential horizontal pleiotropy in the MR analyses, we additionally excluded SNPs that exhibited significant associations (*p* < 1 × 10^−5^) with key confounding factors such as mood swings, blood pressure, and blood glucose, as identified in the PhenoScanner database (http://www.phenoscanner.medschl.cam.ac.uk/, accessed on 1 September 2023) [17,18].

SNPs highly associated with the outcome variables (*p* < 5 × 10^−8^) were further excluded. The GWAS summary data for the exposure and outcome variables were aligned and harmonized to guarantee uniformity in the gene sequencing, ensuring that effect estimates were consistently assigned to the same alleles. Palindromic SNPs (those with alleles on the forward strand matching those on the reverse strand) with intermediate effect-allele frequencies were excluded due to their ambiguous effect directions. Proxy SNPs were not used in this study [19].

Additionally, weak instrument bias, where SNPs showed weak associations with morning diurnal preference, could reduce the robustness of the causal estimates and lead to underpowered results. Therefore, the F-statistic, an indicator of instrument strength, was calculated for each SNP using the following formula [20,21]:

$$F=\frac{R^{2}(N-2)}{1-R^{2}}$$

$$R^{2}=2\times EAF\times \left(1-EAF\right)\times \beta^{2}$$

where *EAF* denotes the effect-allele frequency, and *β* is the estimate for the genetic effect of the SNP on the exposure variable. The *F*-statistic could characterize the proportion of variance (*R*^2^) in the exposure phenotype explained by the genetic instrumental variables. The bias of the instrumental variable estimator equals 1/*F* of the bias of the observational estimator, and an *F*-statistic >10 could effectively avoid weak instrumental variable bias [19]. (The method for the *F*-statistic computations is shown in Tables S1 and S2).

#### 2.2.2. MR of Morning Diurnal Preference and Psychiatric Disorders

This study coded a binary phenotype variable to characterize morning diurnal preference [13]. Participants who indicated being “Definitely an ‘evening’ person” or “More an ‘evening’ than a ‘morning’ person” were categorized as 0 (controls), while those who identified as “Definitely a ‘morning’ person” or “More a ‘morning’ than ‘evening’ person” were designated as 1 (cases). Participants who responded with “Do not know” or “Prefer not to answer” were coded as missing values. The GWAS comprised a total of 403,195 participants, consisting of 252,287 cases and 150,908 controls. We obtained data on psychiatric disorders from the FinnGen consortium R9 release. Comprehensive information regarding the methods, including the data collection, participating cohorts, genotyping, and data analysis, can be accessed online (https://www.finngen.fi/fi, accessed on 1 September 2023). An overview of the data sources for the instrumental variables utilized in the MR study is provided in Table S3.

#### 2.2.3. Bidirectional Mendelian Randomization Analyses

Univariable MR analyses were conducted to explore the causal effects of morning diurnal preference on psychiatric disorders. The primary findings were derived using the inverse-variance weighted (IVW) model and were calculated based on the Wald ratio estimates (SNP-outcome coefficient divided by SNP-exposure coefficient) of each instrumental variable. Meanwhile, we also performed the weighted median, the MR-Egger regression, weighted mode, and simple mode methods in the sensitivity analyses.

The weighted median method can provide robust effect estimates when up to 50% of the SNPs are weak instrumental variables, where the contribution of one SNP to the empirical distribution is proportional to its weight [22]. The MR-Egger regression method can deal with presence of horizontal pleiotropy by performing a weighted linear regression of the SNP-outcome coefficient and the SNP-exposure coefficient [23]. The weighted mode method groups SNPs based on the similarity of their causal effects and calculates the causal effect by considering the cluster with the highest number of SNPs. This approach can yield an unbiased estimate if the SNPs contributing to the largest cluster are valid [24]. In addition, the simple mode can also provide relatively robust results against pleiotropy [25].

### 2.3. Statistical Analysis

All of the aforementioned findings were presented in the form of odds ratios (ORs) and their corresponding 95% confidence intervals (CIs) for psychiatric disorders when comparing morning persons to non-morning persons. Regarding the reverse causal direction, univariable MR analyses, following the same procedure, were performed to assess the causal effects of psychiatric disorders on morning diurnal preference.

Several additional analyses and sensitivity analyses were further performed to examine the robustness of the causal effects. First, the MR Steiger directionality test was employed to verify the correctness of direction of the casual effects [26]. Second, the intercept of the MR-Egger regression method was examined, where a *p* value < 0.05 indicated the presence of horizontal pleiotropy [23]. Third, Cochran’s Q test was performed to evaluate the heterogeneity in the SNP effect in the IVW model [27]. Fourth, scatter plot, forest plot, funnel plot, and “leave-one-out” analyses were conducted to evaluate whether the effects could be strongly influenced by one single SNP [28]. In addition, for casual effects with heterogeneity (i.e., P for Cochran’s Q < 0.05), we further performed the Mendelian Randomization Pleiotropy RESidual Sum and Outlier (MR-PRESSO) test to identify potential outlier SNPs. We also repeated the previous MR analyses after excluding these outlier SNPs [29].

### 2.4. Power Calculations

We assessed the statistical power of our MR analyses using an online tool (https://shiny.cnsgenomics.com/mRnd/, accessed on 20 September 2023) [30] based on factors such as the sample size of the GWAS data, a Type-I error rate set at 0.05, the proportion of cases, the causal effect, and the proportion of variance (*R*^2^) in the exposure phenotype explained by the genetic instrumental SNPs. The details of our power calculations are provided in Tables S4 and S5.

All the statistical analyses were performed using the R software (R Core Team, version 4.3.1, Vienna, Australia) incorporated with the “TwoSampleMR” [31], “Phenoscanner” [18], and “MRPRESSO” [29] packages. A *p* value < 0.05 (two-sided) indicated statistical significance.

## 3. Results

### 3.1. Genetic Instruments of Exposure and Outcome

After the selection of SNPs with *p* < 5 × 10^−8^, conducting pairwise LD clumping, aligning coding alleles between the exposure and outcome summary statistics, and excluding SNPs associated with potential confounders (refer to Supplementary Table S6), we identified valid instrumental variables (IVs) that adhered to the three fundamental MR assumptions. The study included a total of 403,195 participants with information on morning diurnal preference, of whom 252,287 (62.6%) identified as morning people. Demographic data and information on psychiatric disorders were sourced from the FinnGen consortium R9 release. Table S3 provides details on the GWAS summary statistics and download paths used in the MR study.

### 3.2. Effects of Morning Diurnal Preference on Psychiatric Disorders

**Figure 3:**
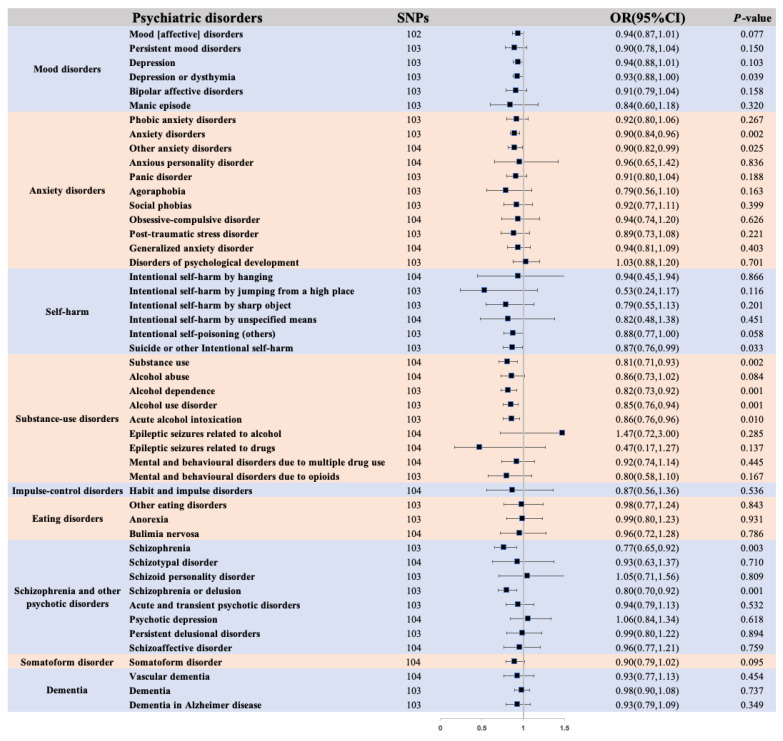
The association between morning diurnal preference and psychiatric disorders using IVW methods.

Results from the univariable MR analyses (Figure 3) revealed a positive effect of morning diurnal preference on the risks of psychiatric disorders. Using the IVW method, individuals identifying as morning people exhibited reduced odds of mood disorders (depression or dysthymia, OR: 0.93, 95% CI: 0.88, 0.999; *p* = 0.039) compared with those identifying as evening people. Similar trends were observed for anxiety disorders (anxiety disorders, OR: 0.90, 95% CI: 0.84, 0.96; *p* = 0.002; other anxiety disorders, OR: 0.90, 95% CI: 0.82, 0.99; *p* = 0.025), as well as self-harm (suicide or other intentional self-harm, OR: 0.87, 95% CI: 0.76, 0.99; *p* = 0.033). Notably, there was consistent evidence of causal effects on substance-use disorders (substance use, OR: 0.81, 95% CI: 0.71, 0.93; *p* = 0.002; alcohol dependence, OR: 0.82, 95% CI: 0.73, 0.92; *p* = 0.001; alcohol use disorder, OR: 0.85, 95% CI: 0.76, 0.94; *p* = 0.001; acute alcohol intoxication, OR: 0.86, 95% CI: 0.76, 0.96; *p* = 0.010). Furthermore, the IVW method indicated a significant association between morning diurnal preference and schizophrenia and other psychotic disorders (schizophrenia, OR: 0.77, 95% CI: 0.65, 0.92; *p* = 0.003; schizophrenia or delusion, OR: 0.80, 95% CI: 0.70, 0.92; *p* = 0.001). Conversely, no substantial causal effects of morning diurnal preference were observed on impulse-control disorders, eating disorders, or dementia. The weighted median and the MR-Egger regression methods providing evidence of morning diurnal preference and psychiatric disorders are shown in Table S7.

In addition, scatterplots, forest plots, leave-one-out plots, and funnel plots for the relationship of morning diurnal preference with psychiatric disorders are shown in Figure S1–S48.

These plots included the scatterplot depicting SNP effects on psychiatric disorders, where the slope of each line corresponded to the estimated MR effect using IVW, weighted median, and MR-Egger methods. Furthermore, the analysis included the forest plot that presented individual and combined SNP MR-estimated effect sizes for the psychiatric disorders. Additionally, the leave-one-out plot was used to visualize how the removal of a single variant influenced the causal estimates (represented as points with horizontal lines) for the effect of morning diurnal preference on psychiatric disorders. Lastly, a funnel plot was employed to assess heterogeneity, with the blue line representing the inverse-variance weighted estimate and the dark blue line representing the MR-Egger estimate.

The MR Steiger directionality test indicated that the correctness in the direction of the casual effects was true (Table S8). No substantial evidence for horizontal pleiotropy was detected in the MR-Egger regression analyses for all analyses (Table S9). The results of the leave-one-out analyses did not indicate that the effects were disproportionately influenced by a single SNP. This was also indicated in the scatterplot, forest plot, and funnel plot analyses (Figures S1–S48). There was evidence for heterogeneity between the casual effects evaluated by Cochran’s Q statistic (i.e., P for Cochran’s Q < 0.05) in the IVW model (Table S10). In addition, we performed the MR-PRESSO test to identify and eliminate potential outlier SNPs, and the results are shown in Table S11.

### 3.3. Reversed Effects of Psychiatric Disorders on Morning Diurnal Preference

**Figure 4:**
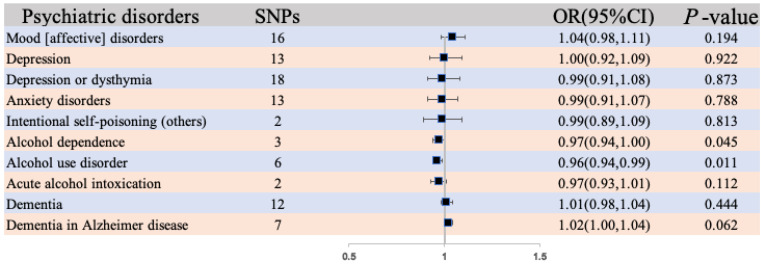
The association between psychiatric disorders and morning diurnal preference using IVW methods.

MR estimates for the effects of the SNPs associated with psychiatric disorders on morning diurnal preference are presented in Figure 4. The IVW method provided evidence that psychiatric disorders (alcohol dependence, OR: 0.97, 95% CI: 0.94, 0.999; *p* = 0.045); alcohol use disorder, OR: 0.96, 95% CI: 0.94, 0.99; *p* = 0.011) were associated with morning diurnal preference. The reversed univariable MR estimates were not statistically significant for the effect of mood (affective) disorders (OR = 1.04; 95% CI: 0.98, 1.11; *p* = 0.194), depression (OR = 1.00; 95% CI: 0.92, 1.09; *p* = 0.922), depression or dysthymia (OR = 0.99; 95% CI: 0.91, 1.08; *p* = 0.873), anxiety disorders (OR = 0.99; 95% CI: 0.91, 1.07; *p* = 0.788), intentional self-poisoning (others) (OR = 0.99; 95% CI: 0.89, 1.09; *p* = 0.813), acute alcohol intoxication (OR = 0.97; 95% CI: 0.93, 1.01; *p* = 0.112), dementia (OR = 1.01; 95% CI: 0.98, 1.04; *p* = 0.444), and dementia in Alzheimer disease (OR = 1.02; 95% CI: 1.00, 1.04; *p* = 0.062) on morning diurnal preference. The weighted median and the MR-Egger regression providing evidence between psychiatric disorders and morning diurnal preference are shown in Table S12.

In addition, Figures S49–S58 show the Mendelian randomization plots that elucidated the associations between psychiatric disorders and morning diurnal preference. These visualizations encompassed a scatterplot illustrating the influence of SNPs on morning diurnal preference, where each line’s slope reflected the estimated MR effect obtained through IVW, weighted median, and MR-Egger methods. Furthermore, the analysis included a forest plot presenting both individual and aggregated SNP MR-estimated effect sizes for various psychiatric disorders. Additionally, a leave-one-out plot was employed to visually demonstrate how the removal of a single variant impacted the causal estimates, depicted as data points with accompanying horizontal lines, pertaining to the influence of psychiatric disorders on morning diurnal preference. Lastly, a funnel plot was utilized to assess heterogeneity, with the blue line representing the inverse-variance weighted estimate and the dark blue line denoting the MR-Egger estimate.

The MR Steiger directionality test validated the correctness of the causal effect directions (Table S13). Across all analyses in the MR-Egger regression, there was no substantial evidence of horizontal pleiotropy (Table S14). Leave-one-out analyses provided no indication of disproportionate influence by a single SNP on the effects, a finding consistent with our scatter plot, forest plot, and funnel plot analyses. However, we did observe evidence of heterogeneity among the causal effects, as indicated by Cochran’s Q statistic (i.e., P for Cochran’s Q < 0.05) in the IVW model (Table S15). To address potential outlier SNPs, we conducted the MR-PRESSO test, with the results presented in Table S16.

## 4. Discussion

In this two-sample bidirectional Mendelian randomization study, we used genetic variants as instrumental variables for morning diurnal preference and evaluated its relationships with various psychiatric disorders. We observed evidence indicating a protective causal effect of morning diurnal preference and mood disorders, anxiety disorders, self-harm, substance-use disorders, schizophrenia, and other psychotic disorders. The results were robust across different MR methods, indicating that our results were unlikely to be related to horizontal pleiotropy. We also conducted a reverse MR analysis and found bidirectional causation between lower alcohol misuse risk and morning-type diurnal preference.

Previous population-based observational studies [6] have reported the potential protective effect of morning diurnal preference against depression and schizophrenia. To reduce the influence of confounding factors, genetic approaches such as the MR method have emerged [14,32], providing more robust evidence that morning people have a lower risk of developing depression and schizophrenia. A large MR analysis [13] involving 697,828 individuals from UK Biobank and 23andMe found that being a morning person was associated with reduced odds of schizophrenia, depression, and better subjective wellbeing. However, the relationship between diurnal preference and self-harm, substance use of alcohol, and other psychotic disorders have been largely explored from observational studies [33,34,35] or animal experiments [36]. Given the highly overlapping nature of various psychiatric disorders [15], it is important to explore the common underlying factors that may mediate similar phenotypic presentations across distinguished but related disorders in order to seek possible universal intervention strategies to reduce disease risk.

Several biological mechanisms may explain the benefits of being a morning person in reducing the risk of psychiatric disorders. In the central nervous system, serotonin (5-hydroxytryptamine, 5-HT) modulates a broad spectrum of functions, including mood, cognition, anxiety, learning, memory, reward processing, and sleep. These processes are mediated through 5-HT binding to 5-HT receptors (5-HTRs) and result in various pathological conditions when deficits occur [37]. Misalignment of the circadian rhythm also disrupts melatonin secretion, which acts on various pathways involved in the progression of psychiatric disorders [38]. Studies found that decreased melatonin levels and circadian disruptions were common in patients with schizophrenia [39] and other psychiatric disorders [40], while specific melatonin supplements and agonists could help restore normal circadian rhythms [41]. Several other studies reported that people with major depressive disorder exhibited advanced biological rhythms and melatonin secretion, suggesting that these changes were more pronounced in mental disorders [42]. Experimental studies also found that unanticipated daytime melatonin secretion on a simulated night-shift schedule could generate a distinctive 24 h melatonin rhythm with antiphrastic daytime and night-time peaks [43], and this may therefore cause further physiological and pathological changes. Meanwhile, evidence suggests that serotonergic signaling is regulated by melatonin, although the effects may vary depending on the specific brain structures involved [44], suggesting interactions between serotonin and melatonin secretion in circadian regulation [45]. Overall, there is emerging evidence showing that circadian disruption is accompanied by psychiatric disorders across the lifespan, although the effect might differ by age, disease type, and severity.

Despite the aforementioned hypotheses and validation studies with limited sample sizes, the underlying physiological mechanisms remain incompletely understood. Much of the current research on associations between diurnal preference and psychiatric disorders focuses on a narrow range of disorders, such as schizophrenia and depression. However, observation studies suggest that various psychiatric disorders usually occur in clusters, commonly accompanied by unfavorable sleep patterns [46]. This has been especially common during the COVID-19 pandemic over the past few years, which presents significant challenges for the promotion of mental wellbeing in the coming years. Our study, along with the results from previous related studies, demonstrates that circadian misalignment could lead to interruptions to sleep homeostasis and cause psychological vulnerability in the general population [47,48]. Nevertheless, maintaining a morning diurnal preference could be a protective factor. In addition, as we found a reverse causal relationship between decreased alcohol misuse with morning diurnal preference, we also recommend minimizing alcohol intake to maintain mental wellbeing.

While our study benefits from a substantial population size, comprehensive classification of psychiatric disorders, and robust MR analyses, several limitations should be considered when interpreting the findings. Firstly, despite selecting SNPs that are strongly associated with diurnal preference, these genetic variants may not fully explain the overall variance in diurnal preference and, therefore, may not precisely capture individual differences. Secondly, the MR approach characterizes the cumulative lifelong impact of genetic variants and should not be directly interpreted as evidence of clinical intervention. Thirdly, our study may not be generalized to other demographic groups, as the genetic data were predominantly derived from European ancestry. Fourthly, estimates derived from MR studies involving unrelated individuals may exhibit bias stemming from environmental and social factors, such as assortative mating, genetic nurturing, and population structure. Future research could address these limitations by incorporating within-family GWASs, which may help to mitigate these potential biases. Finally, socioeconomic and environmental factors, such as healthcare access and living conditions, play a significant role in the development of mental disorders, and their influence could not be fully addressed in this study.

## 5. Conclusions

In conclusion, using large-exposure and outcome GWAS databases to conduct MR analyses, we found robust genetic evidence that morning diurnal preference had protective effects on multiple psychiatric disorders, including mood disorders, anxiety disorders, self-harm, substance-use disorders, and schizophrenia. A reversed causal relationship was also found between alcohol misuse and morning diurnal preference. Results of our study suggest that being a morning person would help to promote overall psychiatric wellbeing. Further research is warranted to extend these findings to diverse populations beyond those of European ancestry and to account for the influence of external factors such as social environment and lifestyle behaviors.

## Data Availability

All data are available in the main text or the Supplementary Materials. Morning diurnal preference data was obtained from genome-wide association study (GWAS) datasets and the 252,287 individuals identified as morning people; psychiatric disorders were sourced from the FinnGen consortium R9 dataset. Further inquiries can be directed to the corresponding authors.

## Supplementary Materials

The following supporting information can be downloaded at: https://www.mdpi.com/article/10.3390/life14101225/s1, Supplementary Table S1: Summary information on diurnal preference SNPs used as genetic instruments for the Mendelian randomization analyses; Supplementary Table S2: Summary information on psychiatric disorders SNPs used as genetic instruments for the Mendelian randomization analyses; Supplementary Table S3: GWAS summary statistics: overview of the data sources of the instrumental variables used in the MR study; Supplementary Table S4: Post hoc power calculations for Mendelian randomization analyses performed at varying causal effect sizes; Supplementary Table S5: Post hoc power calculations for Mendelian randomization analyses performed at varying causal effect sizes; Supplementary Table S6: Potential confounders of morning person SNPs with *p* < 1 × 10^−5^ using the PhenoScanner database; Supplementary Table S7: The association between morning diurnal preference and psychiatric disorders using MR methods; Supplementary Table S8: Results of the MR Steiger direction test for morning diurnal preference on psychiatric disorders; Supplementary Table S9: Inspection of the horizontal pleiotropy through its intercept and provided estimates after correcting for the pleiotropic effects in the MR-Egger regression; Supplementary Table S10: Verification of the heterogeneity between the causal estimates of each SNPs in the IVW and MR-Egger methods; Supplementary Table S11: Identifying outlier SNPs using outlier (MR-PRESSO) test in IVW methods; Supplementary Table S12: The association between psychiatric disorders and morning diurnal preference using MR methods; Supplementary Table S13: Results of the MR Steiger direction test for psychiatric disorders on morning persons; Supplementary Table S14: Inspection of the horizontal pleiotropy through its intercept and provided estimates after correcting for the pleiotropic effects in the MR-Egger regression; Supplementary Table S15: Verification of the heterogeneity between the causal estimates of each SNPs in the IVW and MR-Egger methods; Supplementary Table S16: Identifying outlier SNPs using outlier (MR-PRESSO) test in MR methods; Supplementary Figure S1: Mendelian randomization plots for the relationship of morning diurnal preference with mood (affective) disorders; Supplementary Figure S2: Mendelian randomization plots for the relationship between morning diurnal preference and persistent mood disorders; Supplementary Figure S3: Mendelian randomization plots for the relationship between morning diurnal preference and depression; Supplementary Figure S4: Mendelian randomization plots for the relationship between morning diurnal preference and depression or dysthymia; Supplementary Figure S5: Mendelian randomization plots for the relationship between morning diurnal preference and bipolar affective disorders; Supplementary Figure S6: Mendelian randomization plots for the relationship between morning diurnal preference and manic episode; Supplementary Figure S7: Mendelian randomization plots for the relationship between morning diurnal preference and phobic anxiety disorders; Supplementary Figure S8: Mendelian randomization plots for the relationship between morning diurnal preference and anxiety disorders; Supplementary Figure S9: Mendelian randomization plots for the relationship between morning diurnal preference and other anxiety disorders; Supplementary Figure S10: Mendelian randomization plots for the relationship between morning diurnal preference and anxious personality disorder; Supplementary Figure S11: Mendelian randomization plots for the relationship between morning diurnal preference and panic disorder; Supplementary Figure S12: Mendelian randomization plots for the relationship between morning diurnal preference and agoraphobia; Supplementary Figure S13: Mendelian randomization plots for the relationship between morning diurnal preference and social phobias; Supplementary Figure S14: Mendelian randomization plots for the relationship between morning diurnal preference and obsessive–compulsive disorder; Supplementary Figure S15: Mendelian randomization plots for the relationship between morning diurnal preference and post-traumatic stress disorder; Supplementary Figure S16: Mendelian randomization plots for the relationship between morning diurnal preference and generalized anxiety disorder; Supplementary Figure S17: Mendelian randomization plots for the relationship between morning diurnal preference and disorders of psychological development; Supplementary Figure S18: Mendelian randomization plots for the relationship between morning diurnal preference and intentional self-harm by hanging; Supplementary Figure S19: Mendelian randomization plots for the relationship between morning diurnal preference and intentional self-harm by jumping from a high place; Supplementary Figure S20: Mendelian randomization plots for the relationship between morning diurnal preference and intentional self-harm by a sharp object; Supplementary Figure S21: Mendelian randomization plots for the relationship between morning diurnal preference and intentional self-harm by unspecified means; Supplementary Figure S22: Mendelian randomization plots for the relationship between morning diurnal preference and intentional self-poisoning (others); Supplementary Figure S23: Mendelian randomization plots for the relationship between morning diurnal preference and suicide or other intentional self-harm; Supplementary Figure S24: Mendelian randomization plots for the relationship between morning diurnal preference and substance use; Supplementary Figure S25: Mendelian randomization plots for the relationship between morning diurnal preference and alcohol abuse; Supplementary Figure S26: Mendelian randomization plots for the relationship between morning diurnal preference and alcohol dependence; Supplementary Figure S27: Mendelian randomization plots for the relationship between morning diurnal preference and alcohol use disorder; Supplementary Figure S28: Mendelian randomization plots for the relationship between morning diurnal preference and acute alcohol intoxication; Supplementary Figure S29: Mendelian randomization plots for the relationship between morning diurnal preference and epileptic seizures related to alcohol; Supplementary Figure S30: Mendelian randomization plots for the relationship between morning diurnal preference and epileptic seizures related to drugs; Supplementary Figure S31: Mendelian randomization plots for the relationship between morning diurnal preference and mental and behavioral disorders due to multiple drug use; Supplementary Figure S32: Mendelian randomization plots for the relationship between morning diurnal preference and mental and behavioral disorders due to opioids; Supplementary Figure S33: Mendelian randomization plots for the relationship between morning diurnal preference and habit and impulse disorders; Supplementary Figure S34: Mendelian randomization plots for the relationship between morning diurnal preference and other eating disorders; Supplementary Figure S35: Mendelian randomization plots for the relationship between morning diurnal preference and anorexia; Supplementary Figure S36: Mendelian randomization plots for the relationship between morning diurnal preference and bulimia nervosa; Supplementary Figure S37: Mendelian randomization plots for the relationship between morning diurnal preference and schizophrenia; Supplementary Figure S38: Mendelian randomization plots for the relationship between morning diurnal preference and schizotypal disorder; Supplementary Figure S39: Mendelian randomization plots for the relationship between morning diurnal preference and schizoid personality disorder; Supplementary Figure S40: Mendelian randomization plots for the relationship between morning diurnal preference and schizophrenia or delusion; Supplementary Figure S41: Mendelian randomization plots for the relationship between morning diurnal preference and acute and transient psychotic disorders; Supplementary Figure S42: Mendelian randomization plots for the relationship between morning diurnal preference and psychotic depression; Supplementary Figure S43: Mendelian randomization plots for the relationship between morning diurnal preference and persistent delusional disorders; Supplementary Figure S44: Mendelian randomization plots for the relationship between morning diurnal preference and schizoaffective disorder; Supplementary Figure S45: Mendelian randomization plots for the relationship between morning diurnal preference and somatoform disorder; Supplementary Figure S46: Mendelian randomization plots for the relationship between morning diurnal preference and vascular dementia; Supplementary Figure S47: Mendelian randomization plots for the relationship between morning diurnal preference and dementia; Supplementary Figure S48: Mendelian randomization plots for the relationship between morning diurnal preference and dementia in Alzheimer disease; Supplementary Figure S49: Reversed Mendelian randomization plots for the relationship between mood (affective) disorders and morning diurnal preference; Supplementary Figure S50: Reversed Mendelian randomization plots for the relationship between depression and morning diurnal preference; Supplementary Figure S51: Reversed Mendelian randomization plots for the relationship between depression or dysthymia and morning diurnal preference; Supplementary Figure S52: Reversed Mendelian randomization plots for the relationship between anxiety disorders and morning diurnal preference; Supplementary Figure S53: Reversed Mendelian randomization plots for the relationship between intentional self-poisoning (others) and morning diurnal preference; Supplementary Figure S54: Reversed Mendelian randomization plots for the relationship between alcohol dependence and morning diurnal preference; Supplementary Figure S55: Reversed Mendelian randomization plots for the relationship between alcohol use disorder and morning diurnal preference; Supplementary Figure S56: Reversed Mendelian randomization plots for the relationship between acute alcohol intoxication and morning diurnal preference; Supplementary Figure S57: Reversed Mendelian randomization plots for the relationship between dementia and morning diurnal preference; Supplementary Figure S58: Reversed Mendelian randomization plots for the relationship between dementia in Alzheimer disease and morning diurnal preference.
