# Supplementary material for: Exploring the Causal Association between Morning Diurnal Preference and Psychiatric Disorders: A Bidirectional Two-Sample Mendelian Randomization Analysis
Source: Life (Basel). 2024 Sep 25;14(10):1225. doi: 10.3390/life14101225 (PMC11508865; doi:10.3390/life14101225)
Supplement: Supplementary file 1 [file life-14-01225-s001.zip › Supplementary figures.pdf]

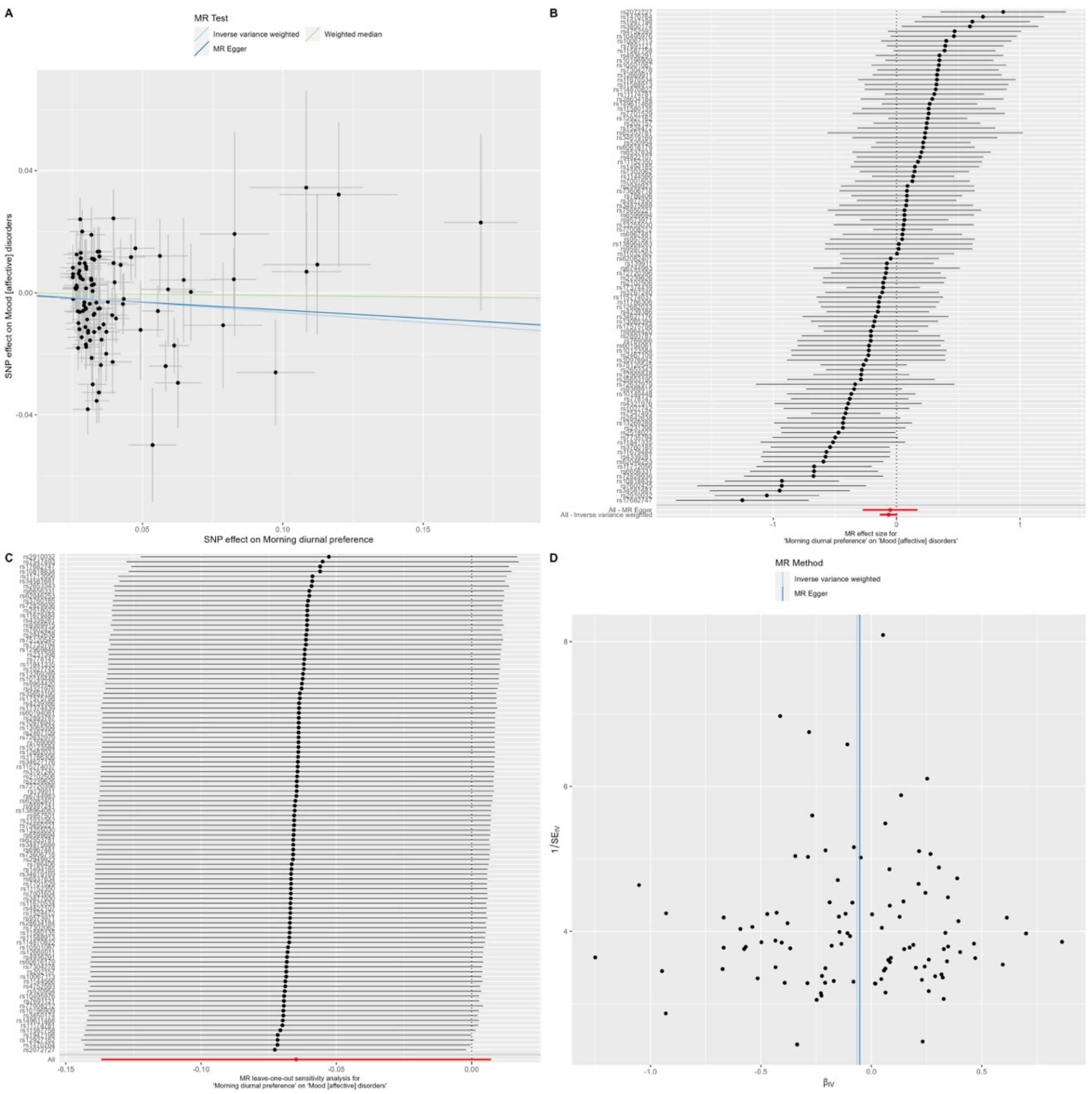

Supplementary Figure1. Mendelian randomization plots for the relationship of morning diurnal preference with mood [affective] disorders

Note: A, Scatterplot of SNP effects on mood [affective] disorders with the slope of each line corresponding to estimated MR effect (IVW, WM, and MR-E methods); B, Forest plot of individual and combined SNP MR-estimated effects sizes for relative mood [affective] disorders; C, The leave-one-out plot visualized how the causal estimates (point with horizontal line) for the effect of morning diurnal preference on mood [affective] disorders were influenced by the removal of single variant; D, Funnel plot assessing heterogeneity. Blue line represents the inverse-variance weighted estimate, and dark blue line represents the MR-Egger estimate.

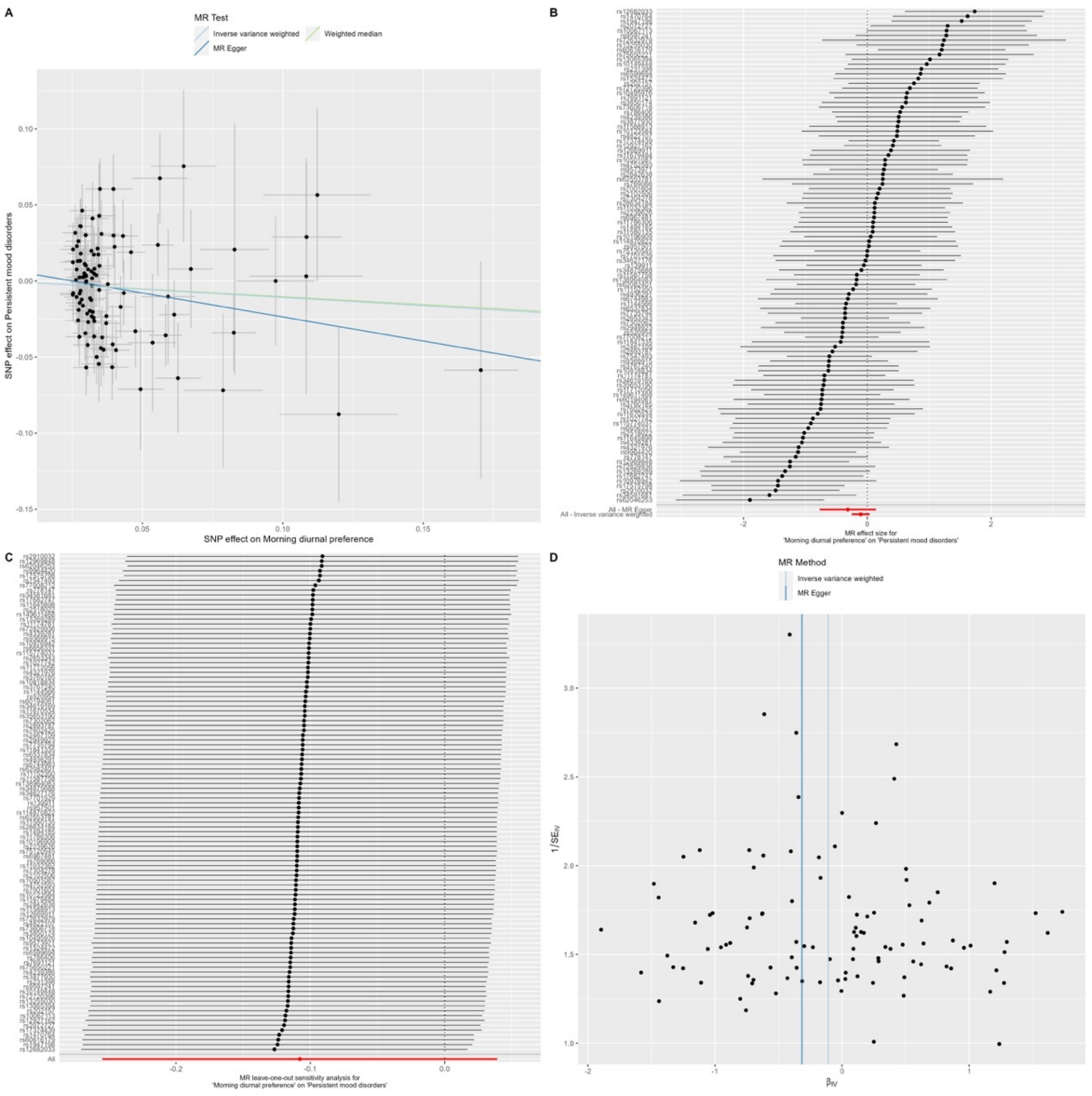

Supplementary Figure2. Mendelian randomization plots for the relationship of morning diurnal preference with persistent mood disorders

Note: A, Scatterplot of SNP effects on persistent mood disorders with the slope of each line corresponding to estimated MR effect (IVW, WM, and MR-E methods); B, Forest plot of individual and combined SNP MR-estimated effects sizes for relative persistent mood disorders; C, The leave-one-out plot visualized how the causal estimates (point with horizontal line) for the effect of morning diurnal preference on persistent mood disorders were influenced by the removal of single variant; D, Funnel plot assessing heterogeneity. Blue line represents the inverse-variance weighted estimate, and dark blue line represents the MR-Egger estimate.

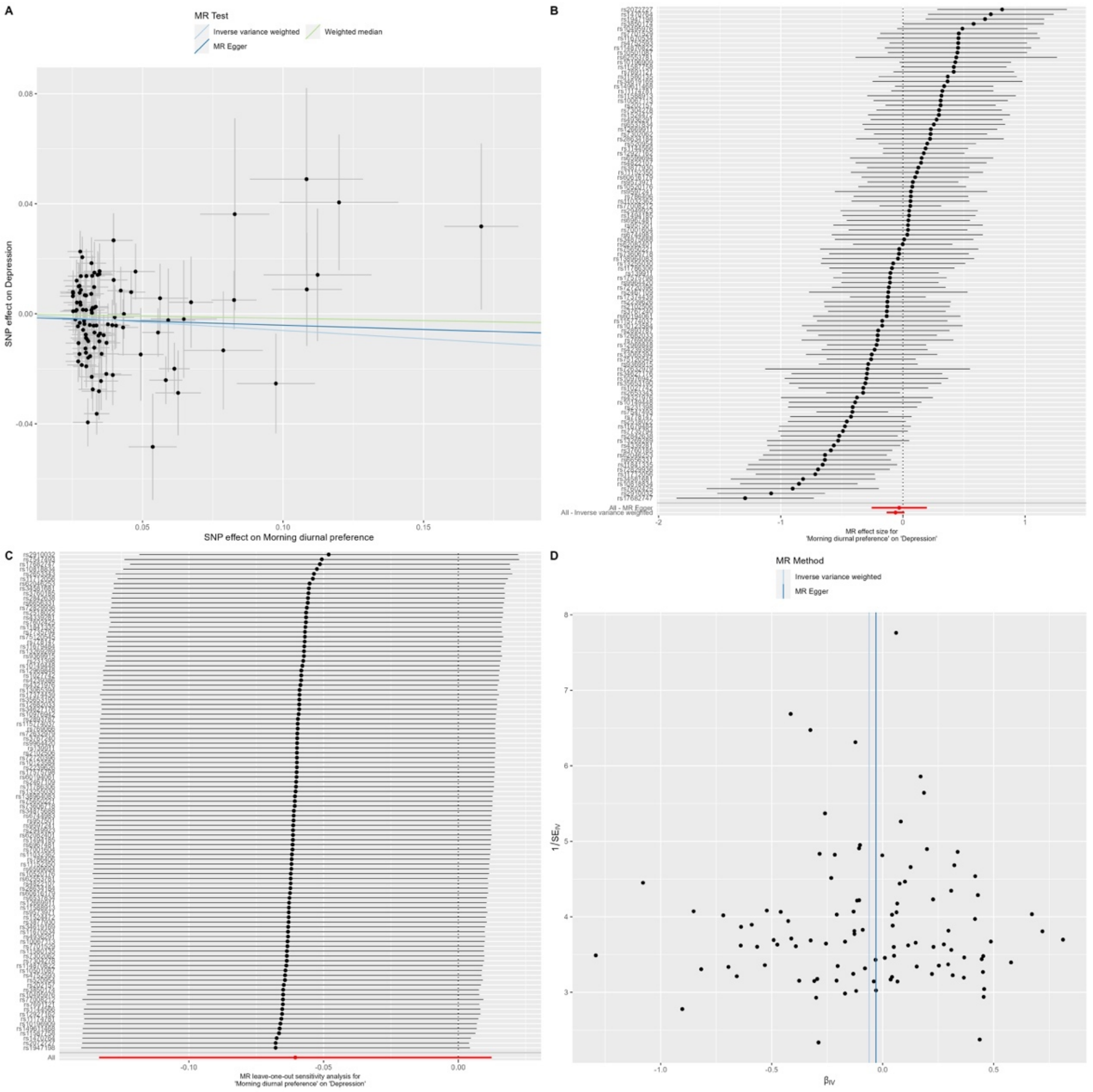

Supplementary Figure3. Mendelian randomization plots for the relationship of morning diurnal preference with depression

Note: A, Scatterplot of SNP effects on depression with the slope of each line corresponding to estimated MR effect (IVW, WM, and MR-E methods); B, Forest plot of individual and combined SNP MR-estimated effects sizes for relative depression; C, The leave-one-out plot visualized how the causal estimates (point with horizontal line) for the effect of morning diurnal preference on depression were influenced by the removal of single variant; D, Funnel plot assessing heterogeneity. Blue line represents the inverse-variance weighted estimate, and dark blue line represents the MR-Egger estimate.

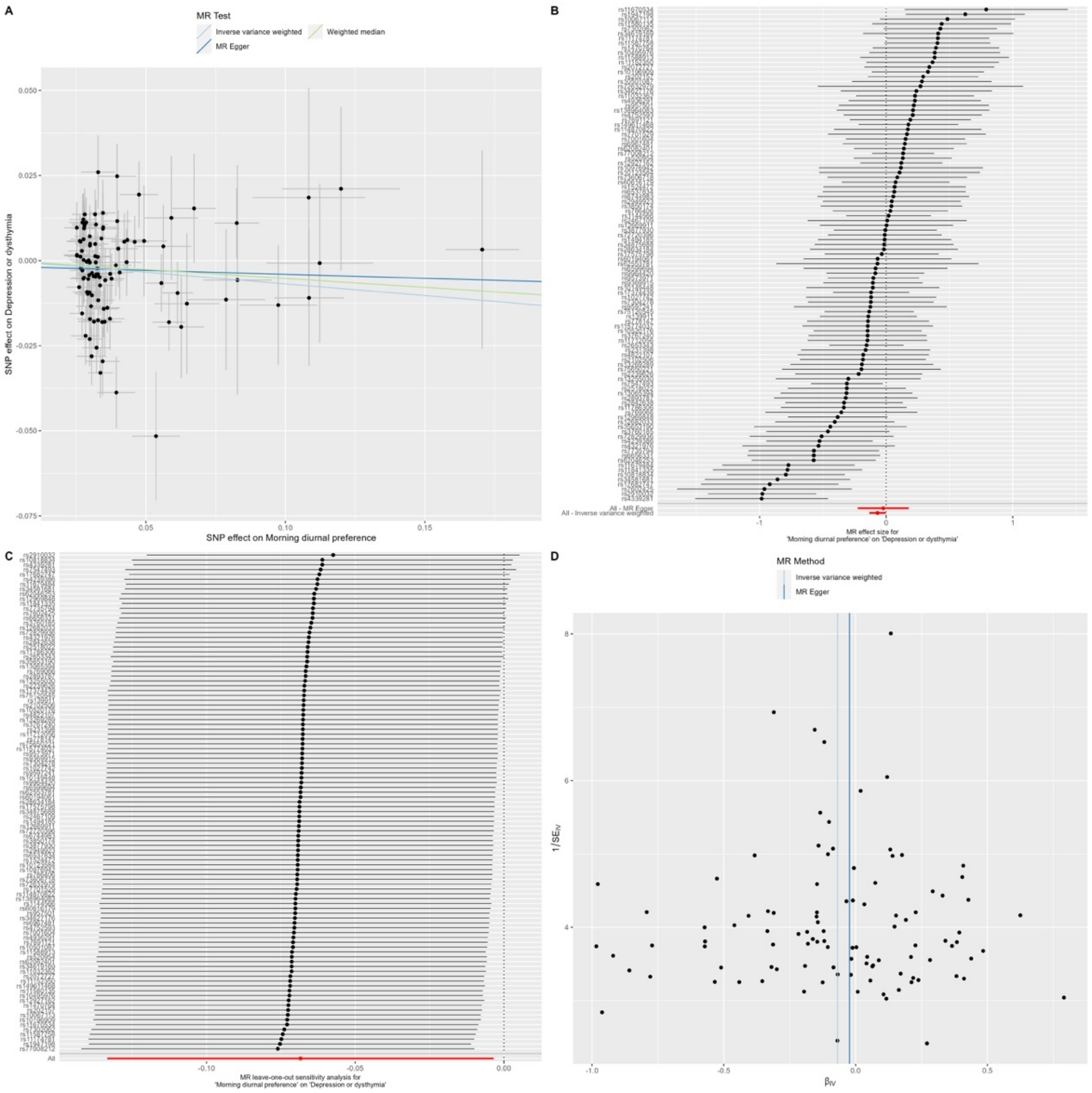

Supplementary Figure4. Mendelian randomization plots for the relationship of morning diurnal preference with depression or dysthymia

Note: A, Scatterplot of SNP effects on depression or dysthymia with the slope of each line corresponding to estimated MR effect (IVW, WM, and MR-E methods); B, Forest plot of individual and combined SNP MR-estimated effects sizes for relative depression or dysthymia; C, The leave-one-out plot visualized how the causal estimates (point with horizontal line) for the effect of morning diurnal preference on depression or dysthymia were influenced by the removal of single variant; D, Funnel plot assessing heterogeneity. Blue line represents the inverse-variance weighted estimate, and dark blue line represents the MR-Egger estimate.

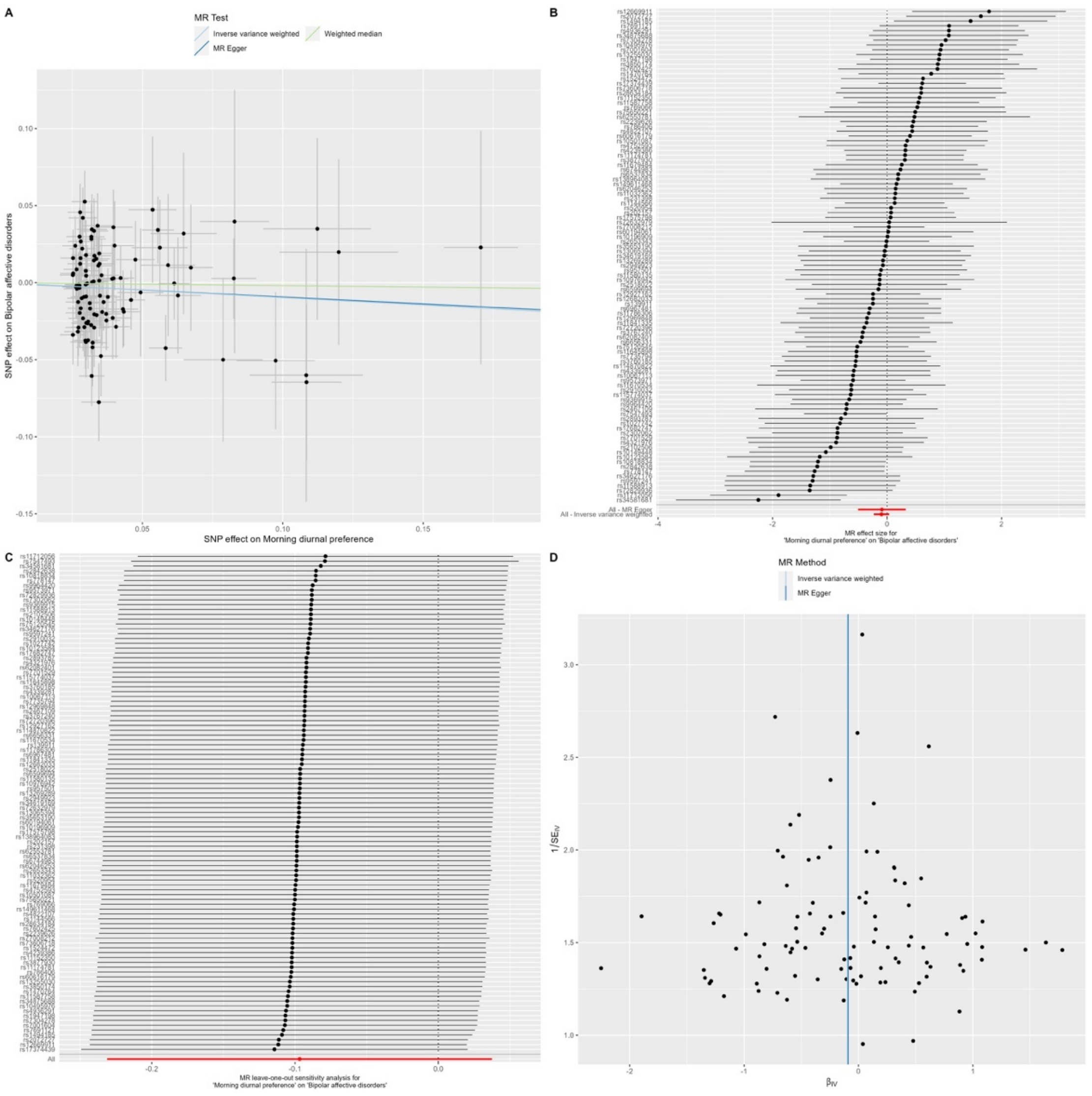

Supplementary Figure5. Mendelian randomization plots for the relationship of morning diurnal preference with bipolar affective disorders

Note: A, Scatterplot of SNP effects on bipolar affective disorders with the slope of each line corresponding to estimated MR effect (IVW, WM, and MR-E methods); B, Forest plot of individual and combined SNP MR-estimated effects sizes for relative bipolar affective disorders; C, The leave-one-out plot visualized how the causal estimates (point with horizontal line) for the effect of morning diurnal preference on bipolar affective disorders were influenced by the removal of single variant; D, Funnel plot assessing heterogeneity. Blue line represents the inverse-variance weighted estimate, and dark blue line represents the MR-Egger estimate.

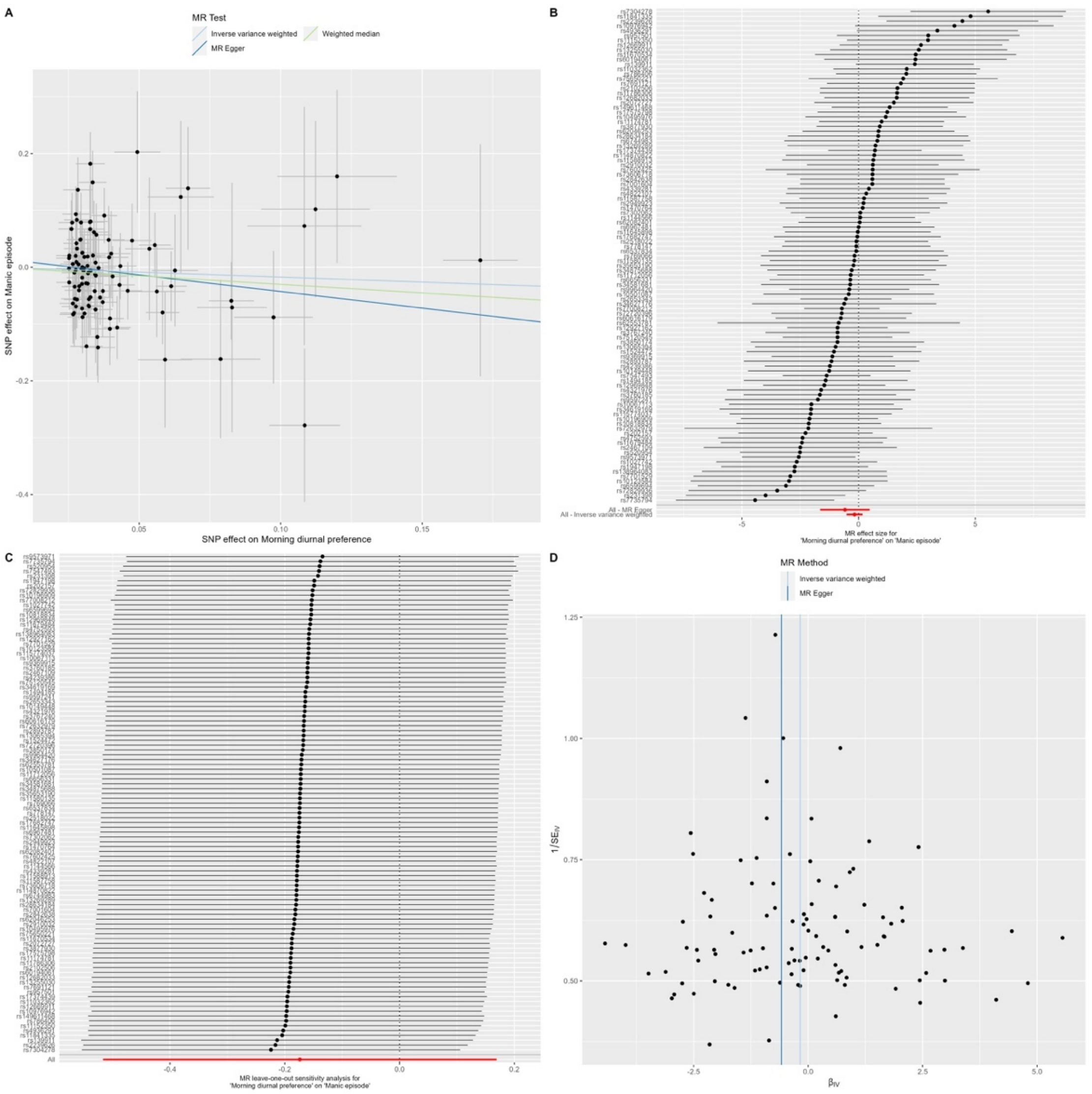

Supplementary Figure6. Mendelian randomization plots for the relationship of morning diurnal preference with manic episode

Note: A, Scatterplot of SNP effects on manic episode with the slope of each line corresponding to estimated MR effect (IVW, WM, and MR-E methods); B, Forest plot of individual and combined SNP MR-estimated effects sizes for relative manic episode; C, The leave-one-out plot visualized how the causal estimates (point with horizontal line) for the effect of morning diurnal preference on manic episode were influenced by the removal of single variant; D, Funnel plot assessing heterogeneity. Blue line represents the inverse-variance weighted estimate, and dark blue line represents the MR-Egger estimate.

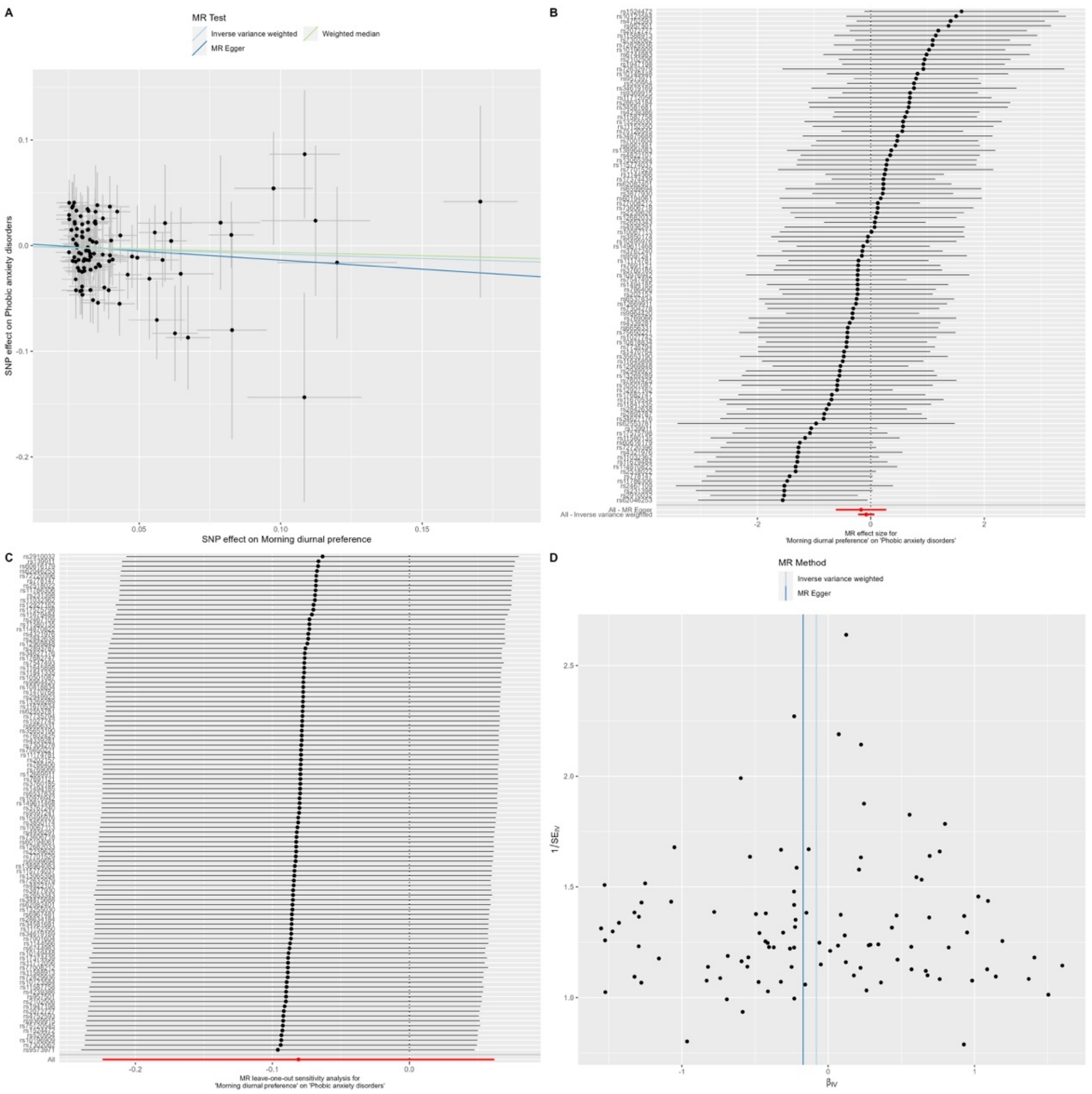

Supplementary Figure7. Mendelian randomization plots for the relationship of morning diurnal preference with phobic anxiety disorders

Note: A, Scatterplot of SNP effects on phobic anxiety disorders with the slope of each line corresponding to estimated MR effect (IVW, WM, and MR-E methods); B, Forest plot of individual and combined SNP MR-estimated effects sizes for relative phobic anxiety disorders; C, The leave-one-out plot visualized how the causal estimates (point with horizontal line) for the effect of morning diurnal preference on phobic anxiety disorders were influenced by the removal of single variant; D, Funnel plot assessing heterogeneity. Blue line represents the inverse-variance weighted estimate, and dark blue line represents the MR-Egger estimate.

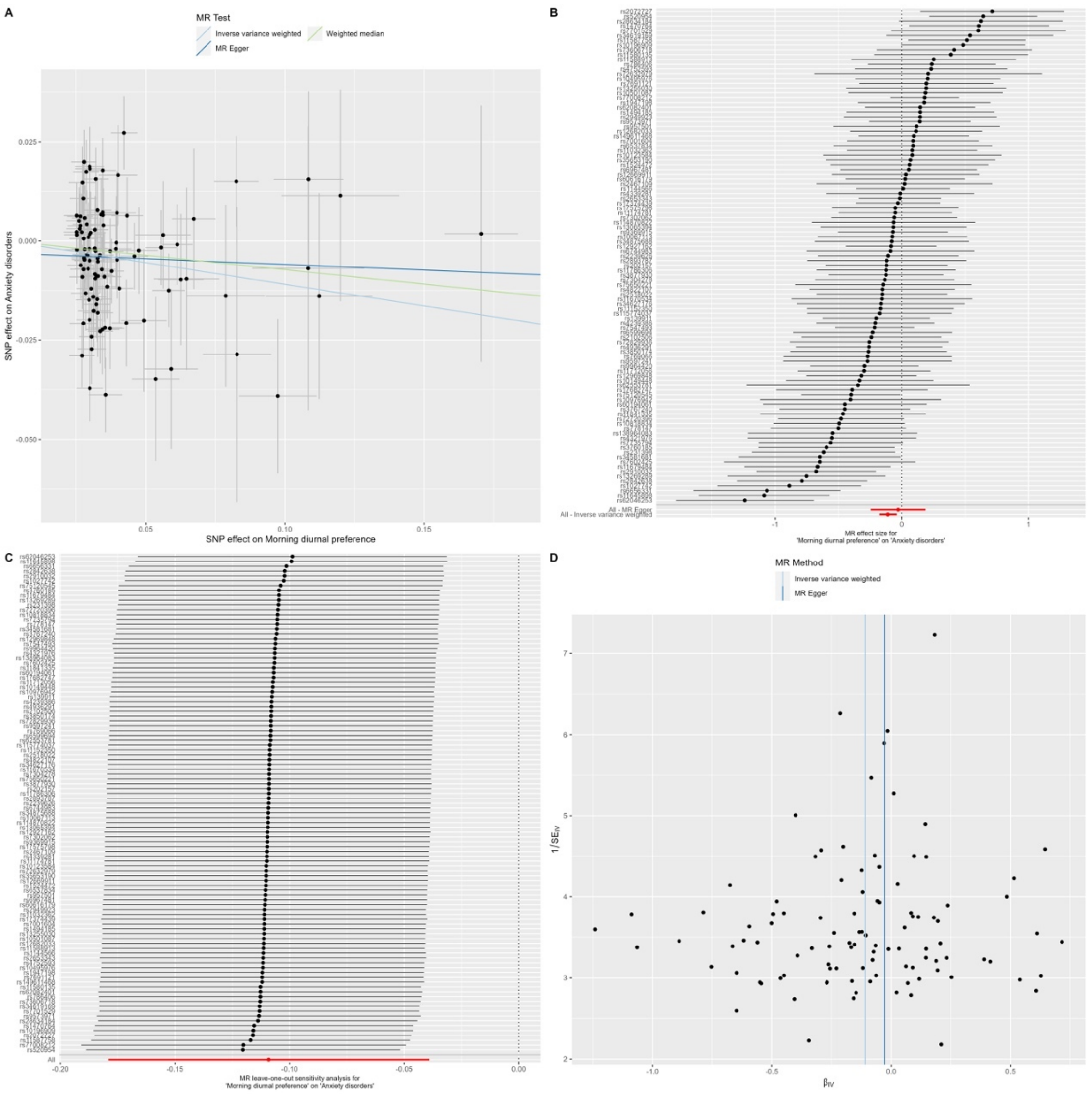

Supplementary Figure8. Mendelian randomization plots for the relationship of morning diurnal preference with anxiety disorders

Note: A, Scatterplot of SNP effects on anxiety disorders with the slope of each line corresponding to estimated MR effect (IVW, WM, and MR-E methods); B, Forest plot of individual and combined SNP MR-estimated effects sizes for relative anxiety disorders; C, The leave-one-out plot visualized how the causal estimates (point with horizontal line) for the effect of morning diurnal preference on anxiety disorders were influenced by the removal of single variant; D, Funnel plot assessing heterogeneity. Blue line represents the inverse-variance weighted estimate, and dark blue line represents the MR-Egger estimate.

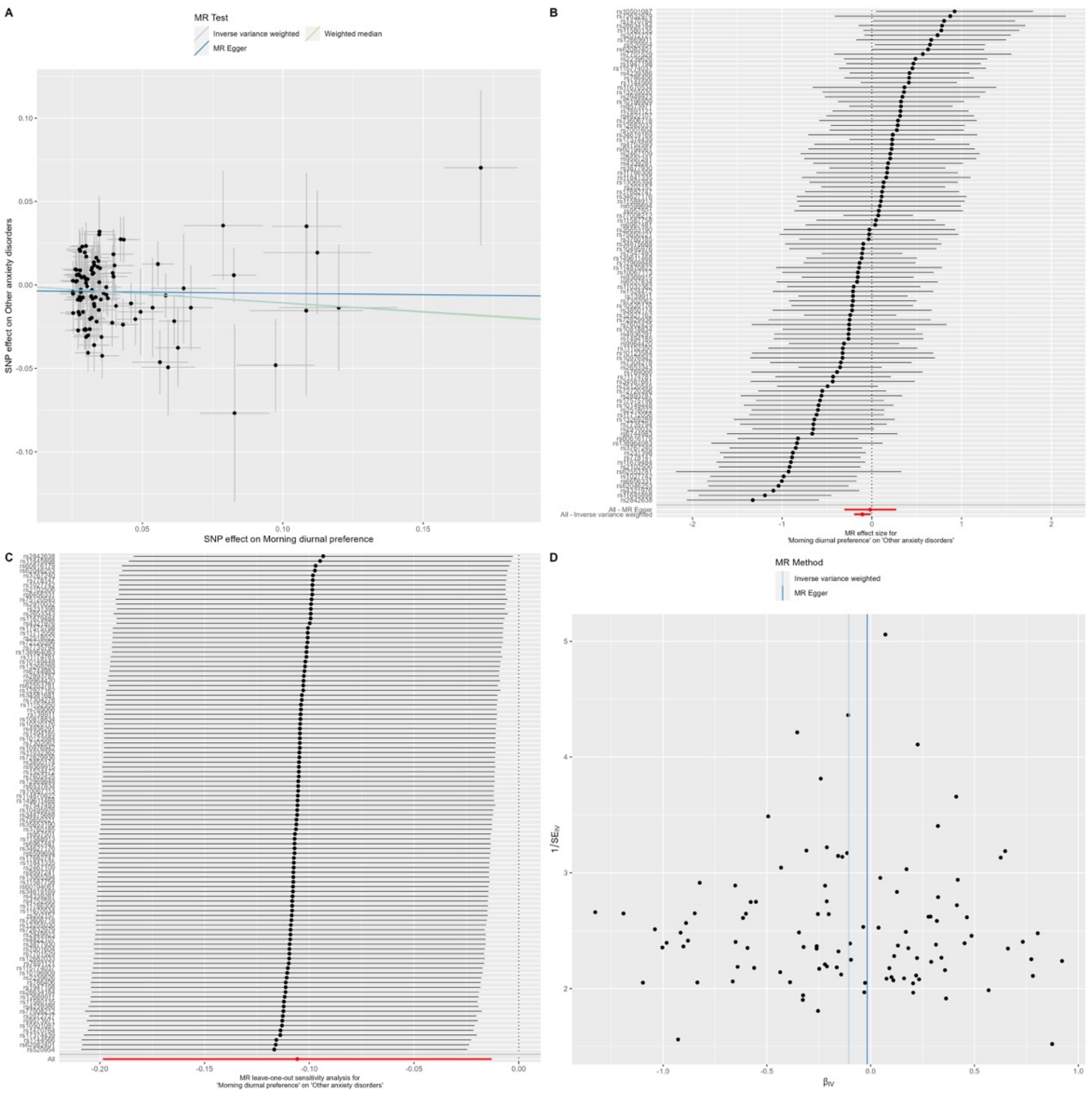

Supplementary Figure9. Mendelian randomization plots for the relationship of morning diurnal preference with other anxiety disorders

Note: A, Scatterplot of SNP effects on other anxiety disorders with the slope of each line corresponding to estimated MR effect (IVW, WM, and MR-E methods); B, Forest plot of individual and combined SNP MR-estimated effects sizes for relative other anxiety disorders; C, The leave-one-out plot visualized how the causal estimates (point with horizontal line) for the effect of morning diurnal preference on other anxiety disorders were influenced by the removal of single variant; D, Funnel plot assessing heterogeneity. Blue line represents the inverse-variance weighted estimate, and dark blue line represents the MR-Egger estimate.

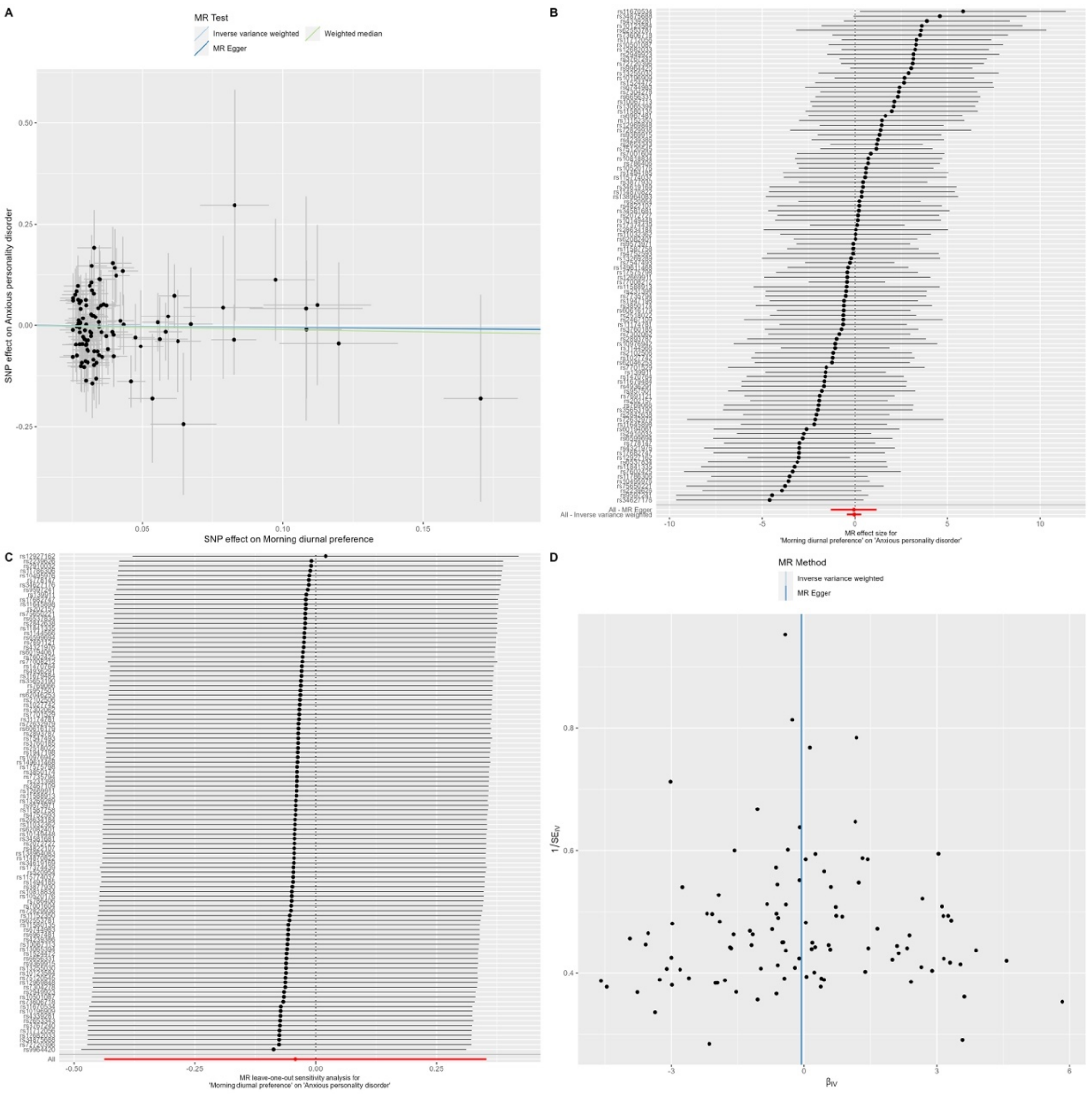

Supplementary Figure10. Mendelian randomization plots for the relationship of morning diurnal preference with anxious personality disorder

Note: A, Scatterplot of SNP effects on anxious personality disorder with the slope of each line corresponding to estimated MR effect (IVW, WM, and MR-E methods); B, Forest plot of individual and combined SNP MR-estimated effects sizes for relative anxious personality disorder; C, The leave-one-out plot visualized how the causal estimates (point with horizontal line) for the effect of morning diurnal preference on anxious personality disorder were influenced by the removal of single variant; D, Funnel plot assessing heterogeneity. Blue line represents the inverse-variance weighted estimate, and dark blue line represents the MR-Egger estimate.

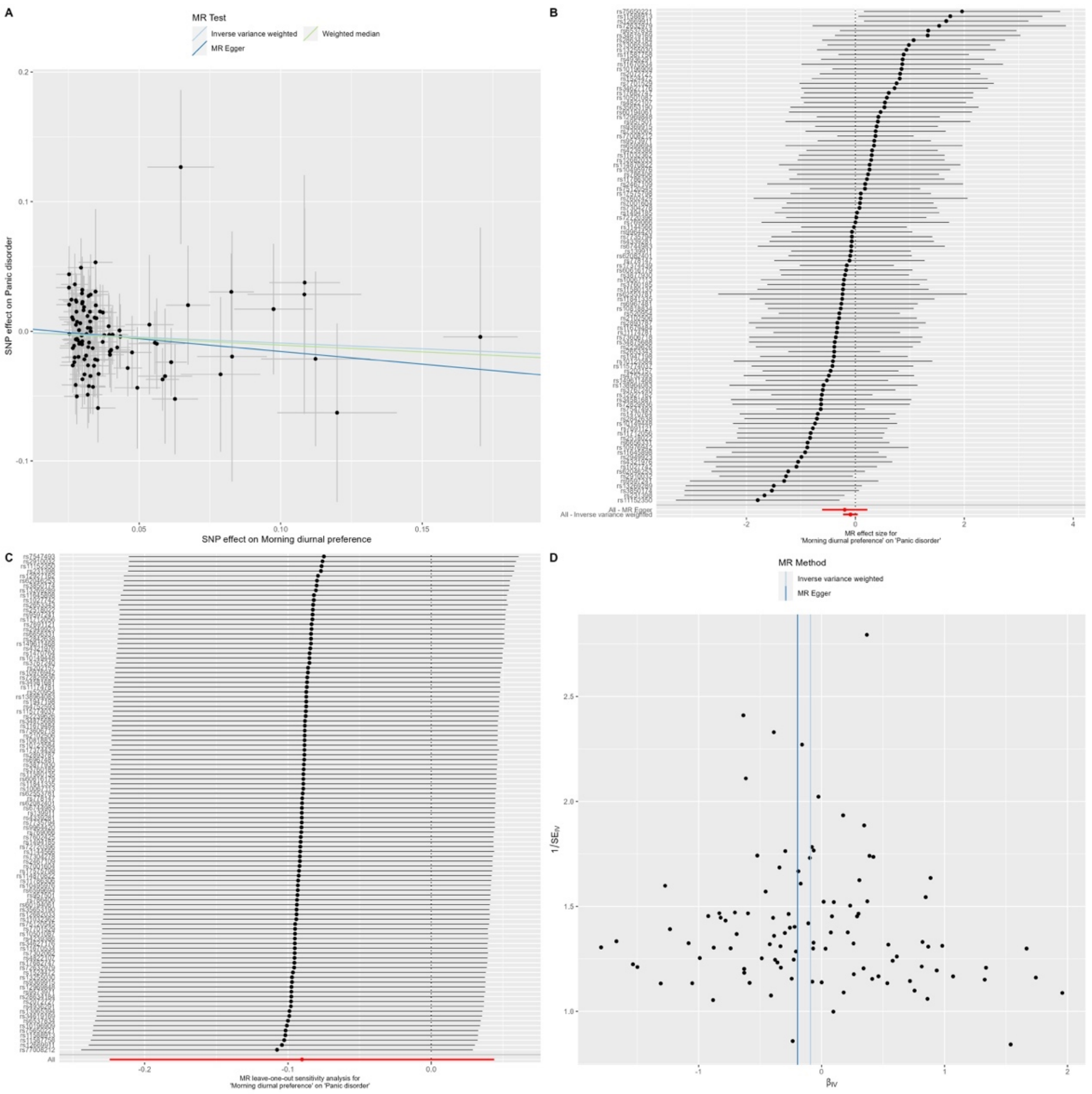

Supplementary Figure11. Mendelian randomization plots for the relationship of morning diurnal preference with panic disorder

Note: A, Scatterplot of SNP effects on panic disorder with the slope of each line corresponding to estimated MR effect (IVW, WM, and MR-E methods); B, Forest plot of individual and combined SNP MR-estimated effects sizes for relative panic disorder; C, The leave-one-out plot visualized how the causal estimates (point with horizontal line) for the effect of morning diurnal preference on panic disorder were influenced by the removal of single variant; D, Funnel plot assessing heterogeneity. Blue line represents the inverse-variance weighted estimate, and dark blue line represents the MR-Egger estimate.

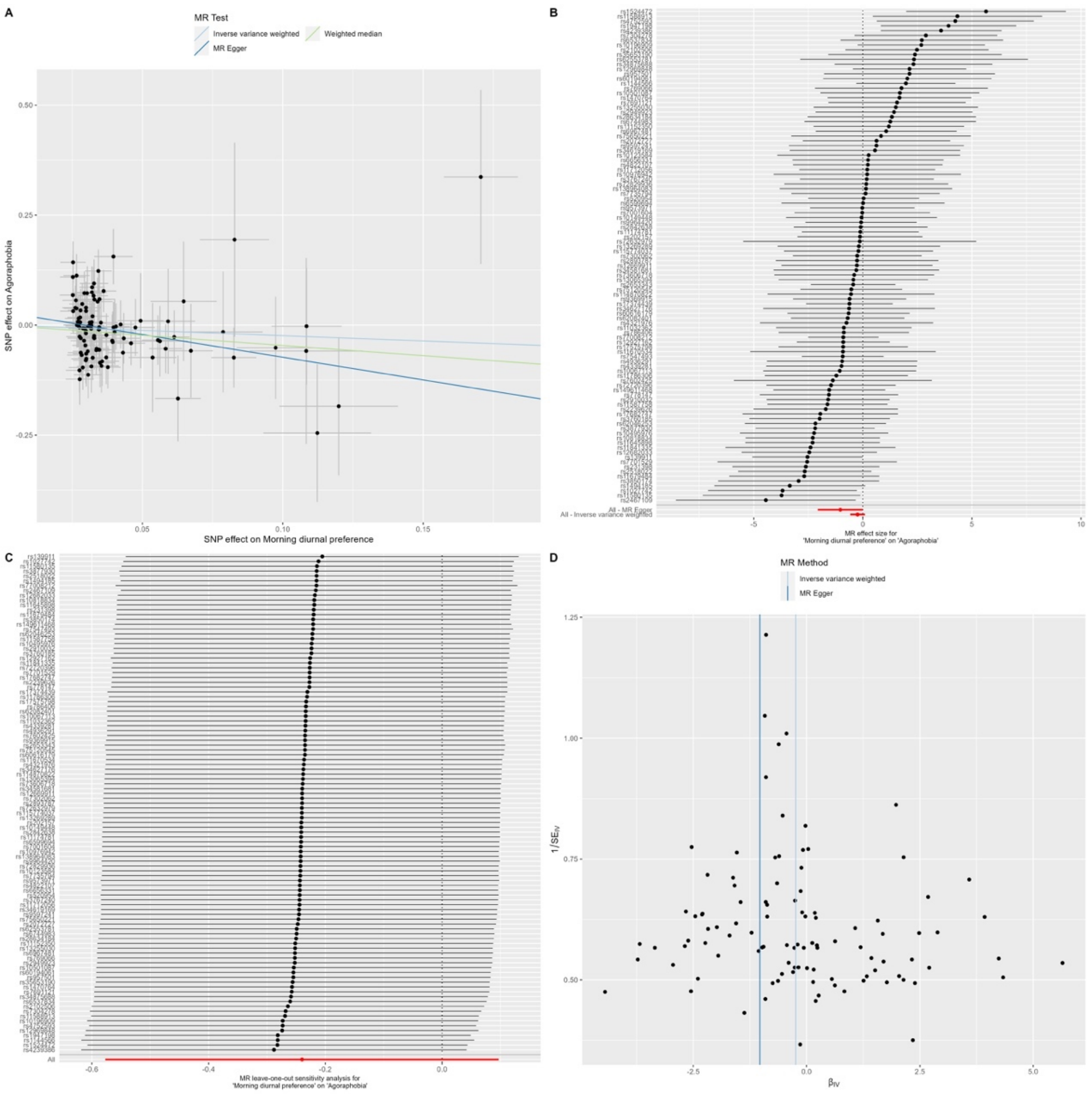

Supplementary Figure12. Mendelian randomization plots for the relationship of morning diurnal preference with agoraphobia

Note: A, Scatterplot of SNP effects on agoraphobia with the slope of each line corresponding to estimated MR effect (IVW, WM, and MR-E methods); B, Forest plot of individual and combined SNP MR-estimated effects sizes for relative agoraphobia; C, The leave-one-out plot visualized how the causal estimates (point with horizontal line) for the effect of morning diurnal preference on agoraphobia were influenced by the removal of single variant; D, Funnel plot assessing heterogeneity. Blue line represents the inverse-variance weighted estimate, and dark blue line represents the MR-Egger estimate.

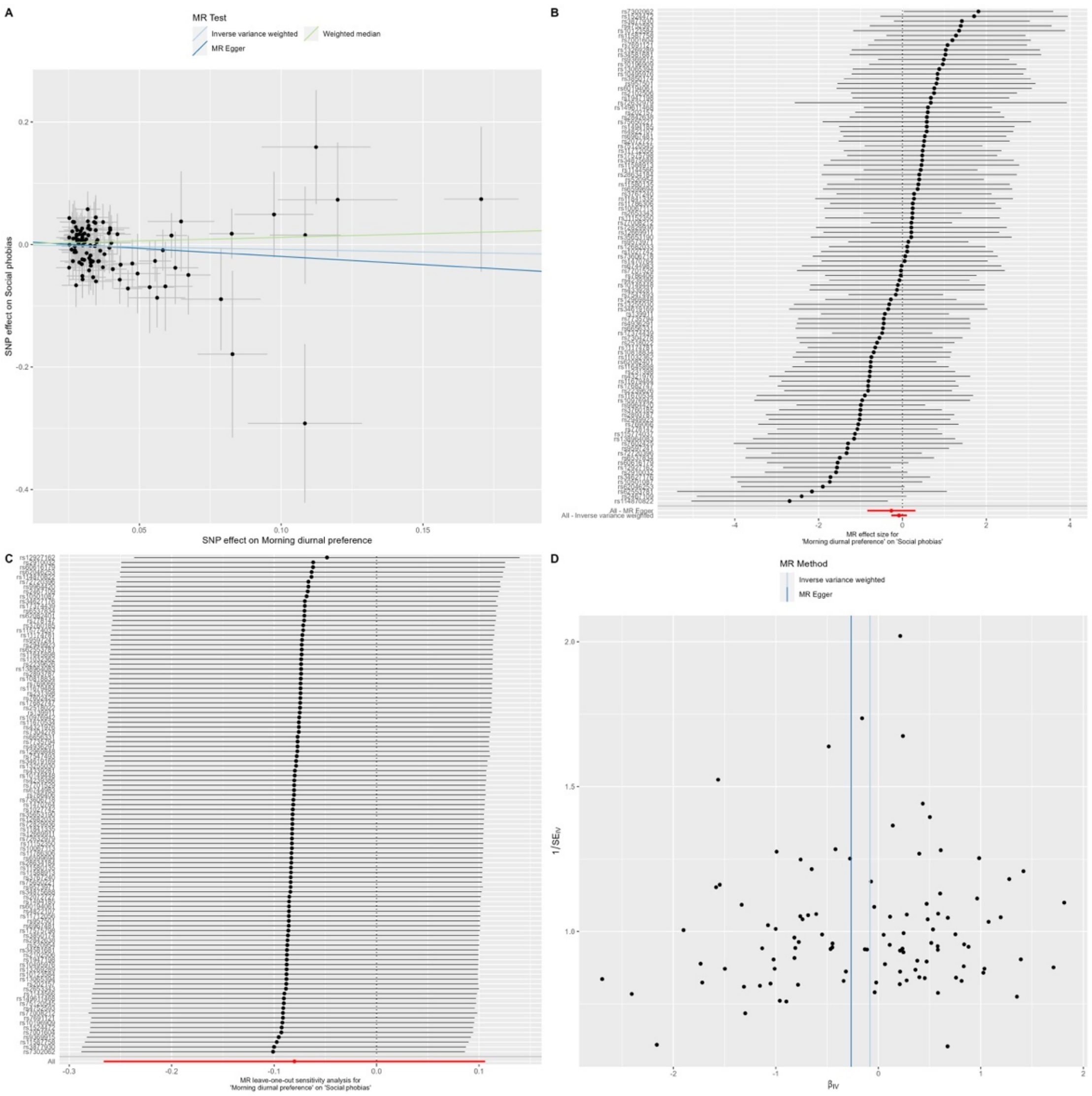

Supplementary Figure13. Mendelian randomization plots for the relationship of morning diurnal preference with social phobias

Note: A, Scatterplot of SNP effects on social phobias with the slope of each line corresponding to estimated MR effect (IVW, WM, and MR-E methods); B, Forest plot of individual and combined SNP MR-estimated effects sizes for relative social phobias; C, The leave-one-out plot visualized how the causal estimates (point with horizontal line) for the effect of morning diurnal preference on social phobias were influenced by the removal of single variant; D, Funnel plot assessing heterogeneity. Blue line represents the inverse-variance weighted estimate, and dark blue line represents the MR-Egger estimate.

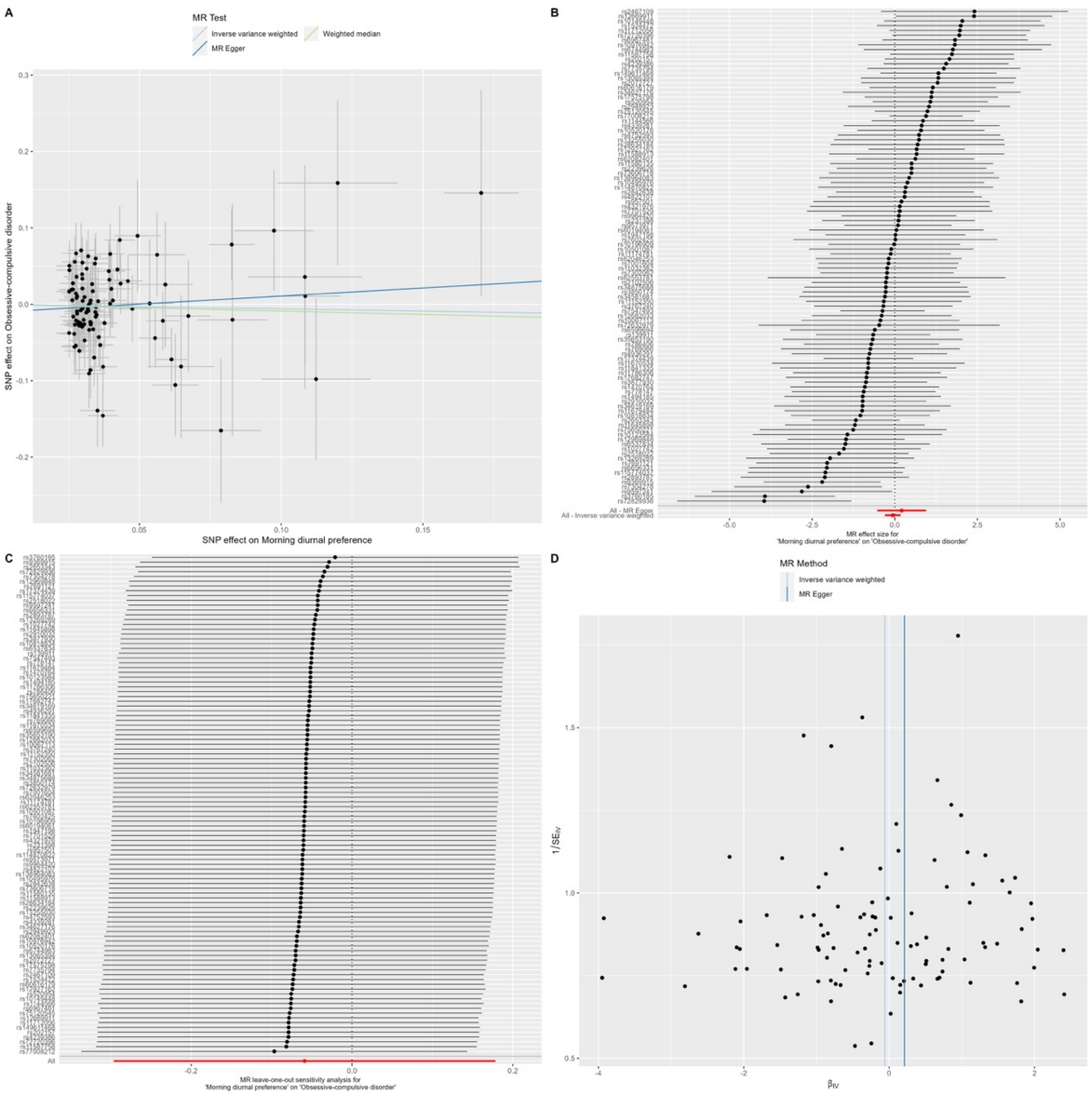

Supplementary Figure14. Mendelian randomization plots for the relationship of morning diurnal preference with obsessive-compulsive disorder

Note: A, Scatterplot of SNP effects on obsessive-compulsive disorder with the slope of each line corresponding to estimated MR effect (IVW, WM, and MR-E methods); B, Forest plot of individual and combined SNP MR-estimated effects sizes for relative obsessive-compulsive disorder; C, The leave-one-out plot visualized how the causal estimates (point with horizontal line) for the effect of morning diurnal preference on obsessive-compulsive disorder were influenced by the removal of single variant; D, Funnel plot assessing heterogeneity. Blue line represents the inverse-variance weighted estimate, and dark blue line represents the MR-Egger estimate.

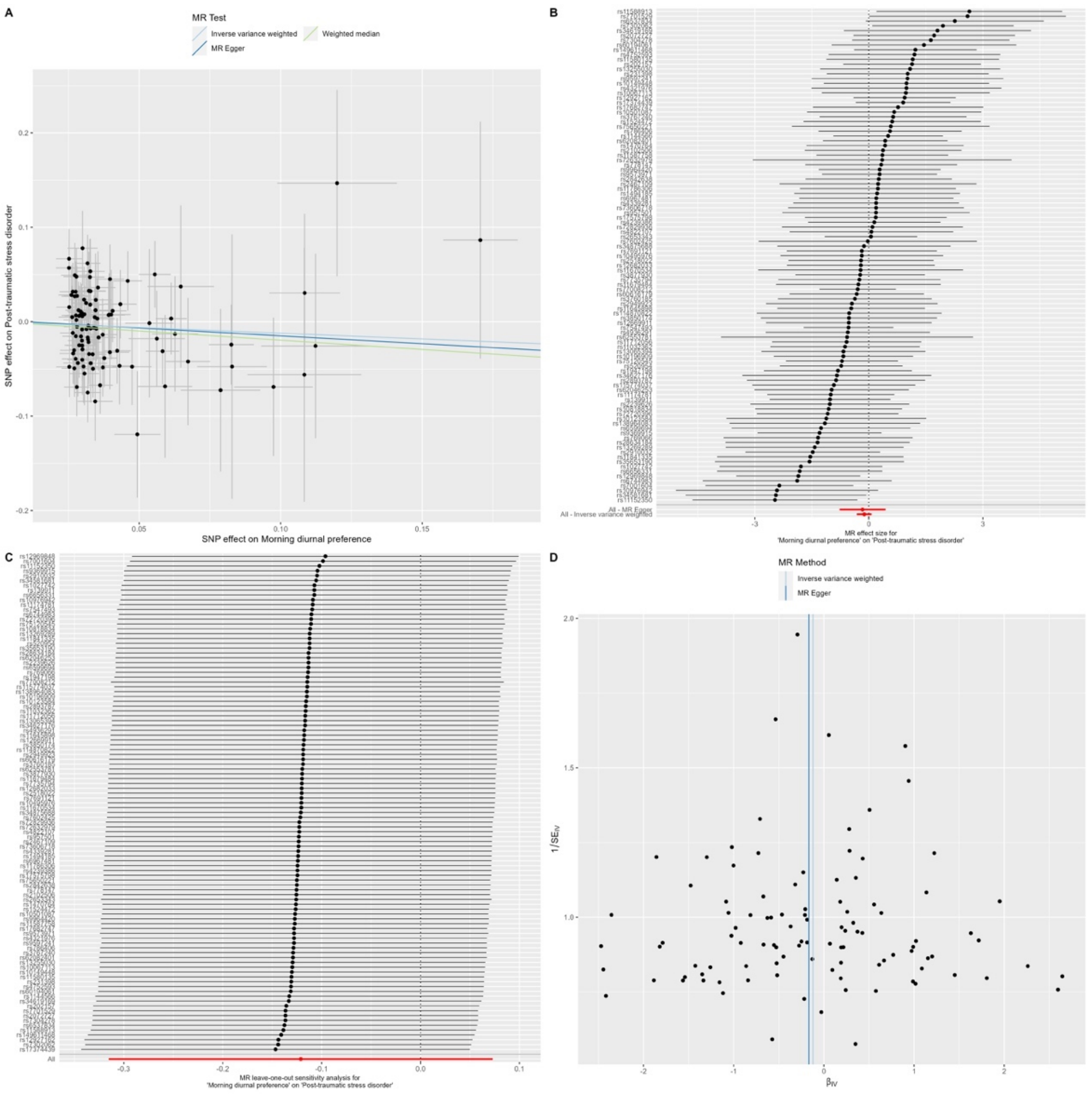

Supplementary Figure15. Mendelian randomization plots for the relationship of morning diurnal preference with post-traumatic stress disorder

Note: A, Scatterplot of SNP effects on post-traumatic stress disorder with the slope of each line corresponding to estimated MR effect (IVW, WM, and MR-E methods); B, Forest plot of individual and combined SNP MR-estimated effects sizes for relative post-traumatic stress disorder; C, The leave-one-out plot visualized how the causal estimates (point with horizontal line) for the effect of morning diurnal preference on post-traumatic stress disorder were influenced by the removal of single variant; D, Funnel plot assessing heterogeneity. Blue line represents the inverse-variance weighted estimate, and dark blue line represents the MR-Egger estimate.

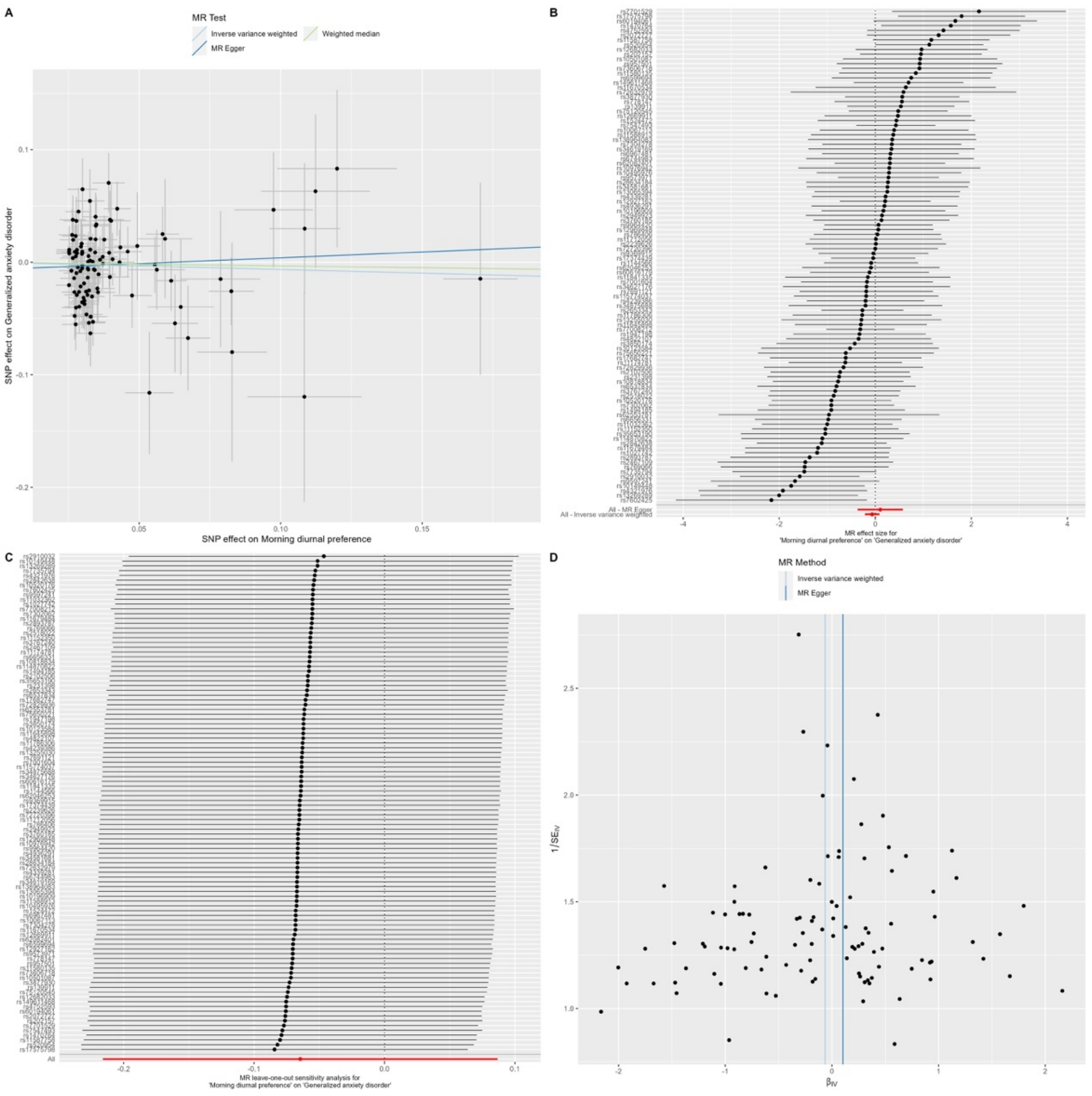

Supplementary Figure16. Mendelian randomization plots for the relationship of morning diurnal preference with generalized anxiety disorder

Note: A, Scatterplot of SNP effects on generalized anxiety disorder with the slope of each line corresponding to estimated MR effect (IVW, WM, and MR-E methods); B, Forest plot of individual and combined SNP MR-estimated effects sizes for relative generalized anxiety disorder; C, The leave-one-out plot visualized how the causal estimates (point with horizontal line) for the effect of morning diurnal preference on generalized anxiety disorder were influenced by the removal of single variant; D, Funnel plot assessing heterogeneity. Blue line represents the inverse-variance weighted estimate, and dark blue line represents the MR-Egger estimate.

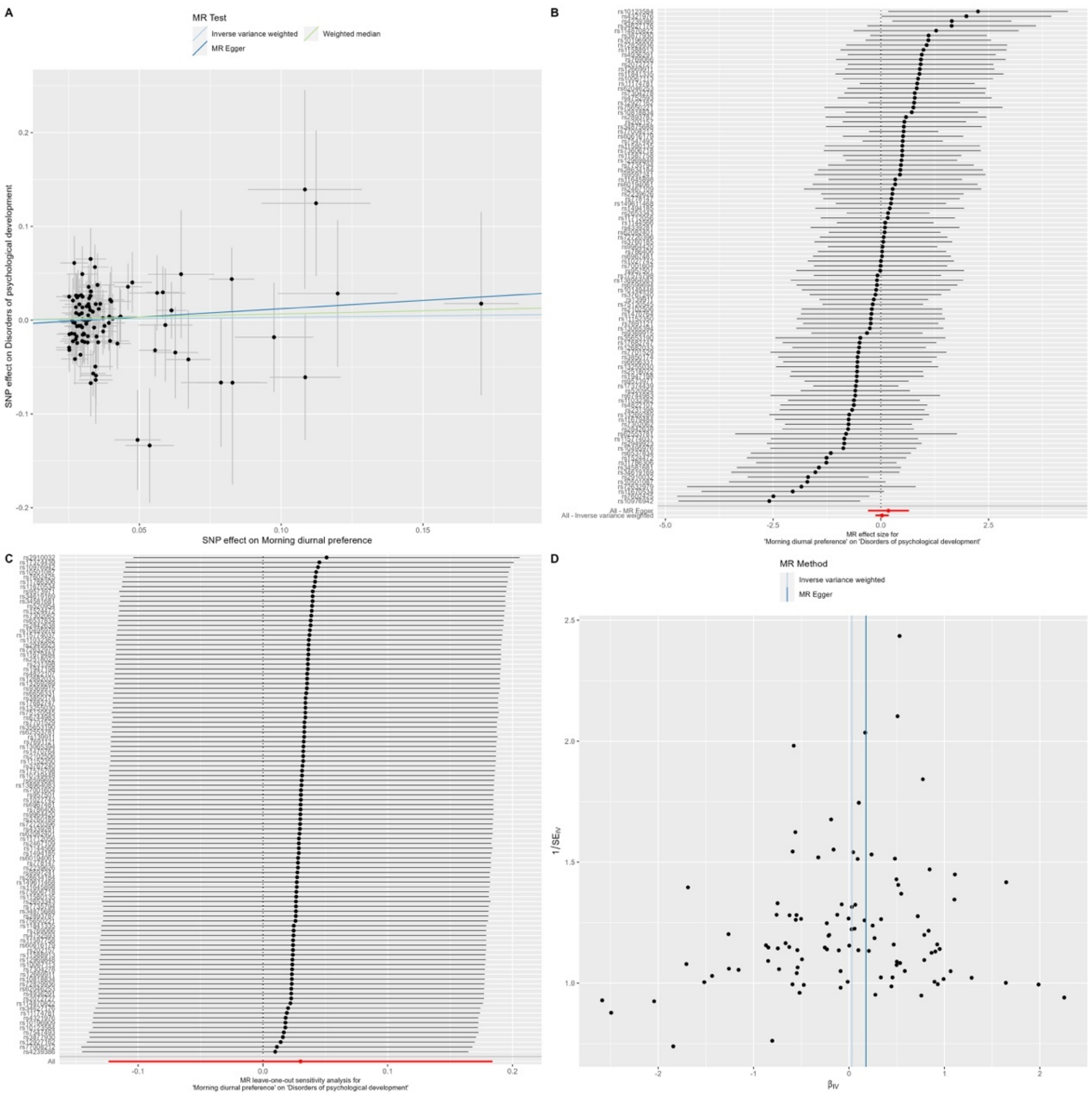

Supplementary Figure17. Mendelian randomization plots for the relationship of morning diurnal preference with disorders of psychological development

Note: A, Scatterplot of SNP effects on disorders of psychological development with the slope of each line corresponding to estimated MR effect (IVW, WM, and MR-E methods); B, Forest plot of individual and combined SNP MR-estimated effects sizes for relative disorders of psychological development; C, The leave-one-out plot visualized how the causal estimates (point with horizontal line) for the effect of morning diurnal preference on disorders of psychological development were influenced by the removal of single variant; D, Funnel plot assessing heterogeneity. Blue line represents the inverse-variance weighted estimate, and dark blue line represents the MR-Egger estimate.

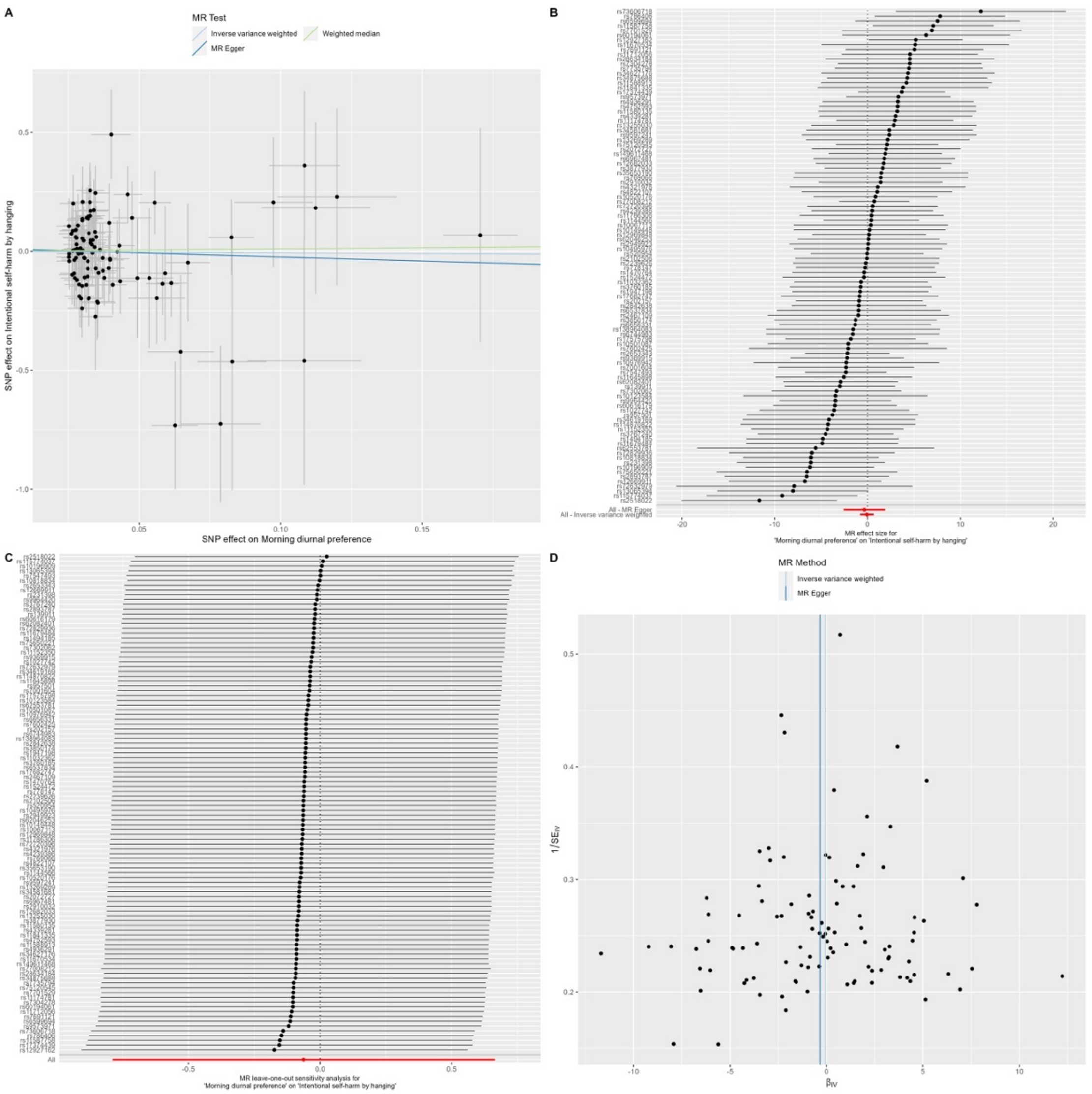

Supplementary Figure18. Mendelian randomization plots for the relationship of morning diurnal preference with intentional self-harm by hanging

Note: A, Scatterplot of SNP effects on intentional self-harm by hanging with the slope of each line corresponding to estimated MR effect (IVW, WM, and MR-E methods); B, Forest plot of individual and combined SNP MR-estimated effects sizes for relative intentional self-harm by hanging; C, The leave-one-out plot visualized how the causal estimates (point with horizontal line) for the effect of morning diurnal preference on intentional self-harm by hanging were influenced by the removal of single variant; D, Funnel plot assessing heterogeneity. Blue line represents the inverse-variance weighted estimate, and dark blue line represents the MR-Egger estimate.

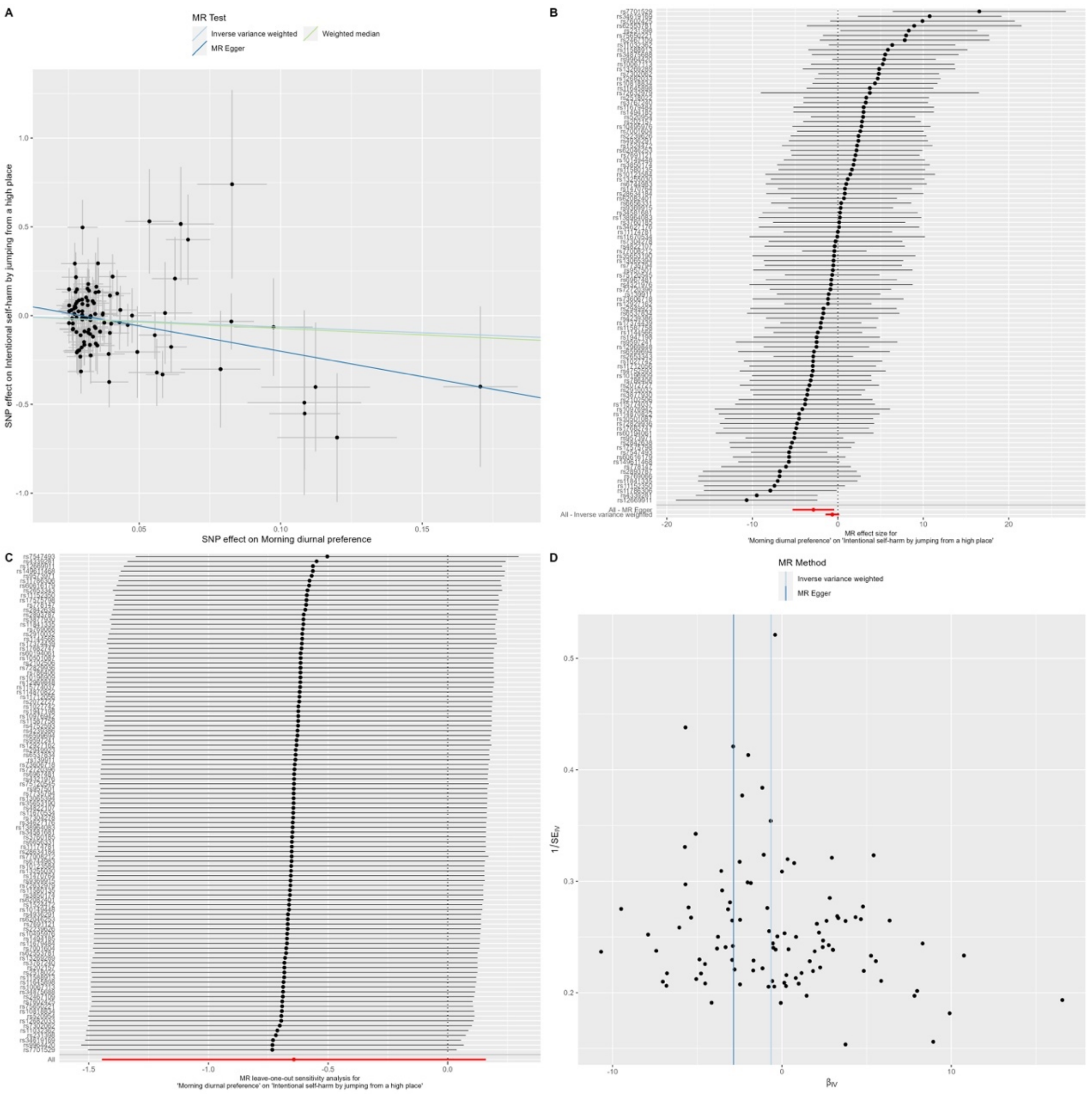

Supplementary Figure19. Mendelian randomization plots for the relationship of morning diurnal preference with intentional self-harm by jumping from a high place

Note: A, Scatterplot of SNP effects on intentional self-harm by jumping from a high place with the slope of each line corresponding to estimated MR effect (IVW, WM, and MR-E methods); B, Forest plot of individual and combined SNP MR-estimated effects sizes for relative intentional self-harm by jumping from a high place; C, The leave-one-out plot visualized how the causal estimates (point with horizontal line) for the effect of morning diurnal preference on intentional self-harm by jumping from a high place were influenced by the removal of single variant; D, Funnel plot assessing heterogeneity. Blue line represents the inverse-variance weighted estimate, and dark blue line represents the MR-Egger estimate.

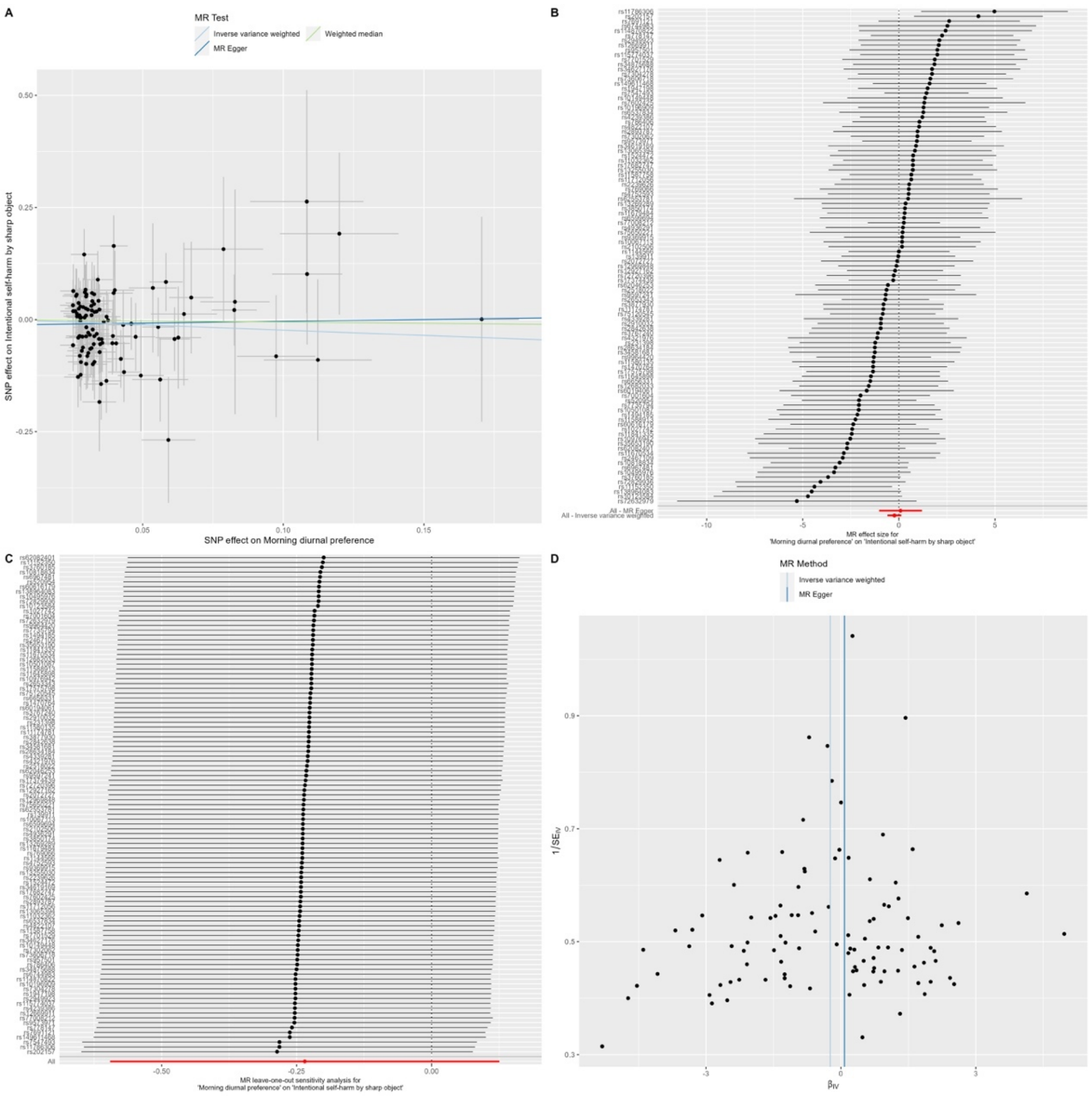

Supplementary Figure20. Mendelian randomization plots for the relationship of morning diurnal preference with intentional self-harm by sharp object

Note: A, Scatterplot of SNP effects on intentional self-harm by sharp object with the slope of each line corresponding to estimated MR effect (IVW, WM, and MR-E methods); B, Forest plot of individual and combined SNP MR-estimated effects sizes for relative intentional self-harm by sharp object; C, The leave-one-out plot visualized how the causal estimates (point with horizontal line) for the effect of morning diurnal preference on intentional self-harm by sharp object were influenced by the removal of single variant; D, Funnel plot assessing heterogeneity. Blue line represents the inverse-variance weighted estimate, and dark blue line represents the MR-Egger estimate.

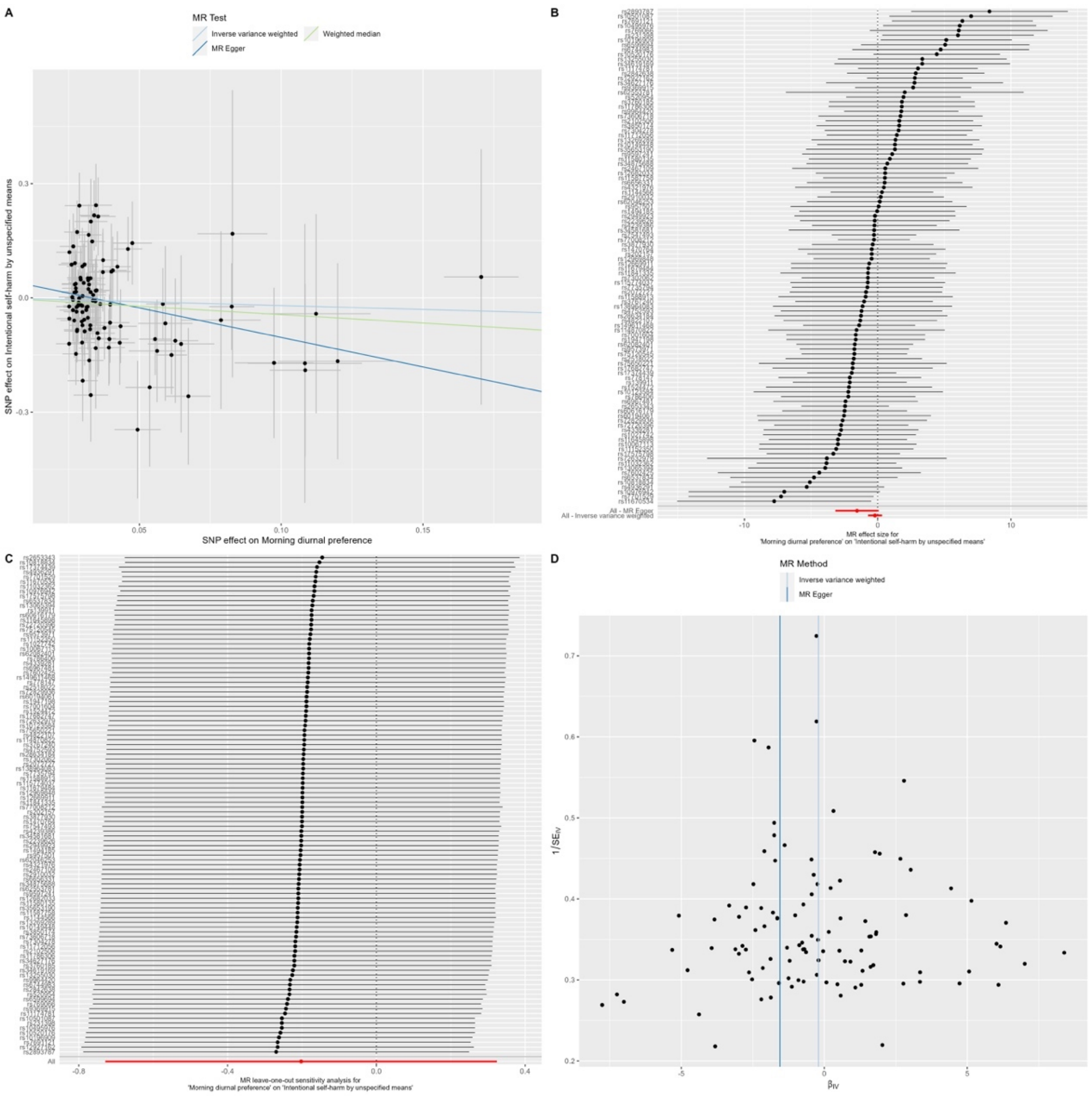

Supplementary Figure21. Mendelian randomization plots for the relationship of morning diurnal preference with intentional self-harm by unspecified means

Note: A, Scatterplot of SNP effects on intentional self-harm by unspecified means with the slope of each line corresponding to estimated MR effect (IVW, WM, and MR-E methods); B, Forest plot of individual and combined SNP MR-estimated effects sizes for relative intentional self-harm by unspecified means; C, The leave-one-out plot visualized how the causal estimates (point with horizontal line) for the effect of morning diurnal preference on intentional self-harm by unspecified means were influenced by the removal of single variant; D, Funnel plot assessing heterogeneity. Blue line represents the inverse-variance weighted estimate, and dark blue line represents the MR-Egger estimate.

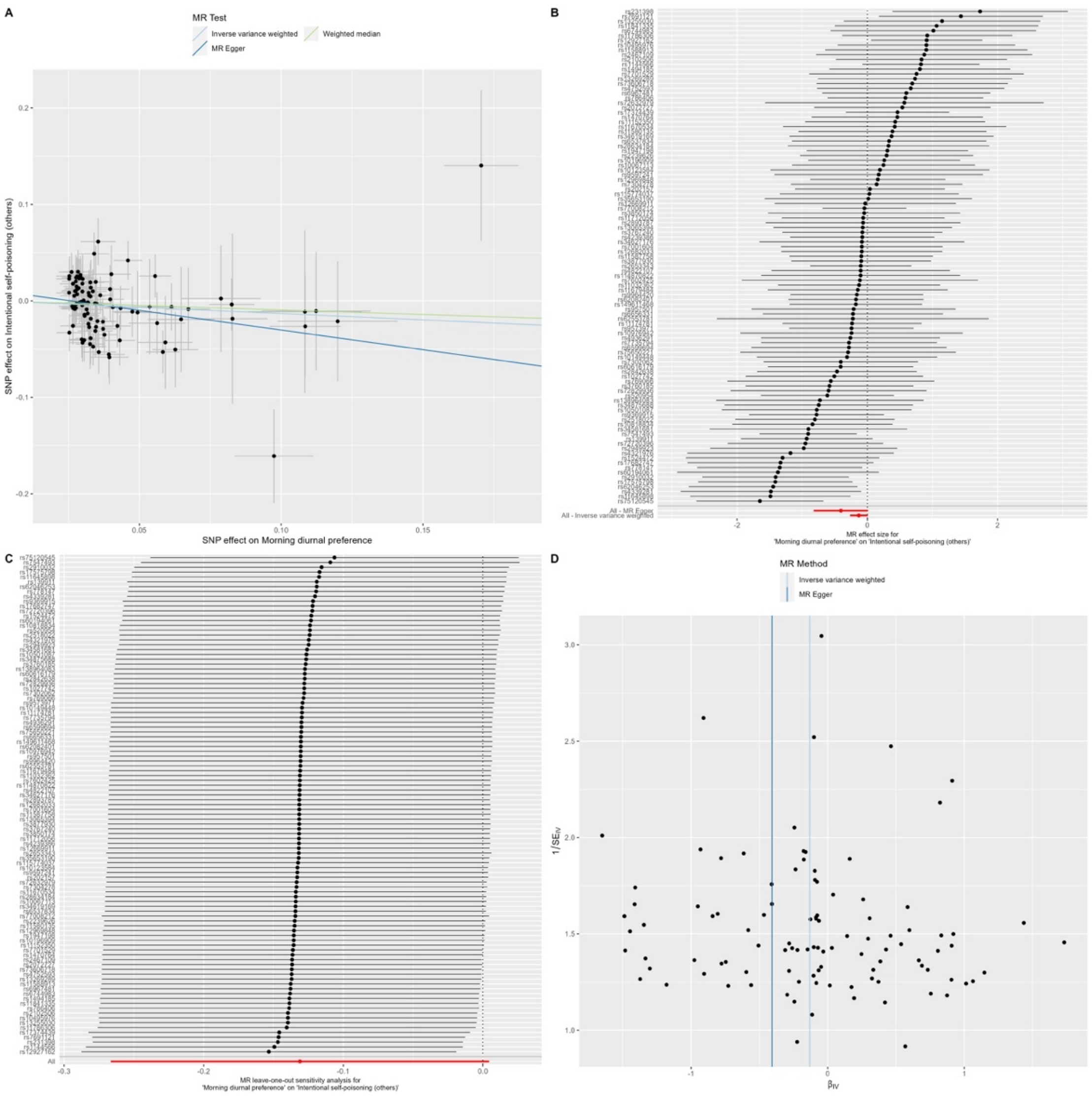

Supplementary Figure22. Mendelian randomization plots for the relationship of morning diurnal preference with intentional self-poisoning (others)

Note: A, Scatterplot of SNP effects on intentional self-poisoning (others) with the slope of each line corresponding to estimated MR effect (IVW, WM, and MR-E methods); B, Forest plot of individual and combined SNP MR-estimated effects sizes for relative intentional self-poisoning (others); C, The leave-one-out plot visualized how the causal estimates (point with horizontal line) for the effect of morning diurnal preference on intentional self-poisoning (others) were influenced by the removal of single variant; D, Funnel plot assessing heterogeneity. Blue line represents the inverse-variance weighted estimate, and dark blue line represents the MR-Egger estimate.

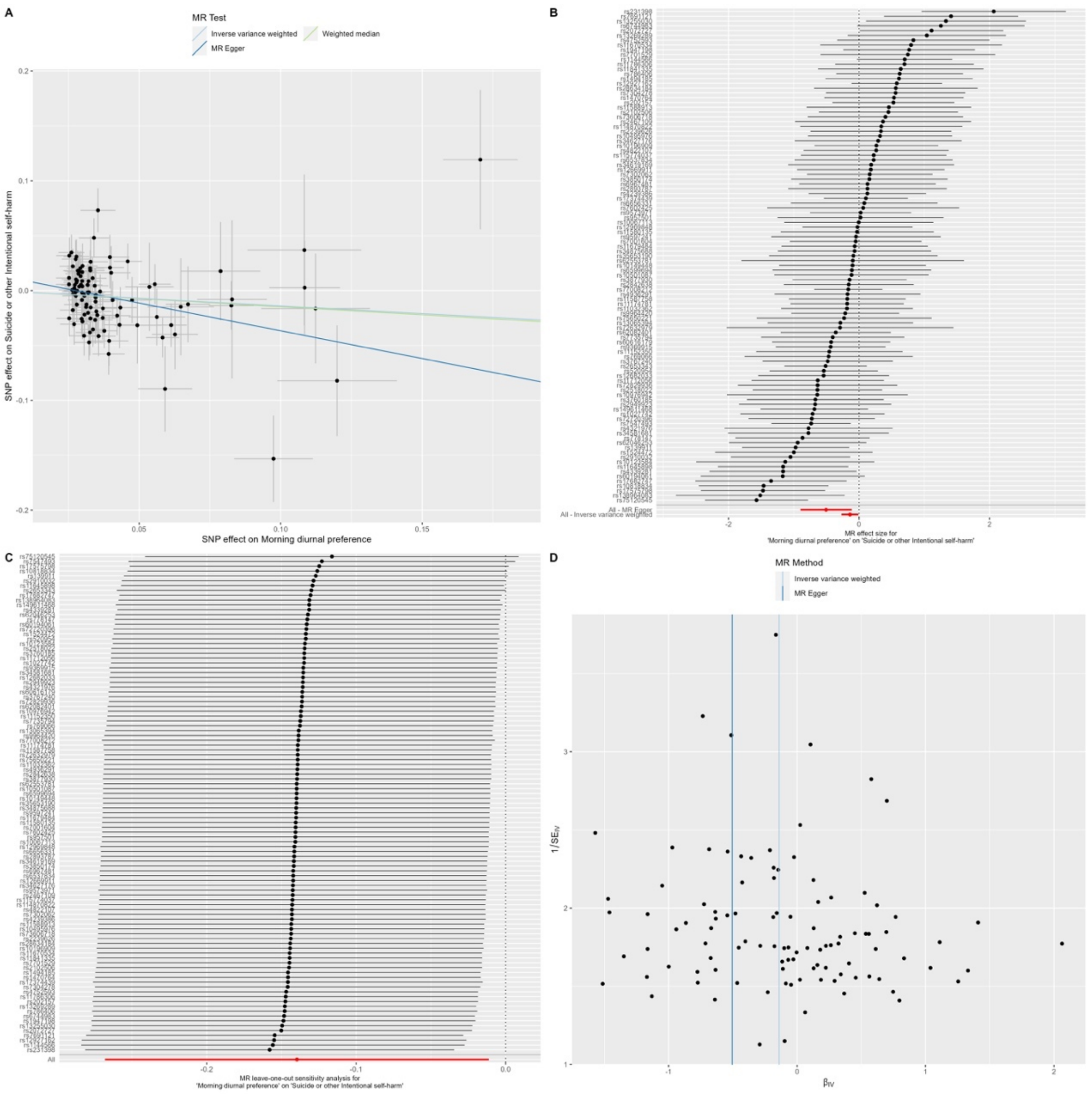

Supplementary Figure23. Mendelian randomization plots for the relationship of morning diurnal preference with suicide or other Intentional self-harm

Note: A, Scatterplot of SNP effects on suicide or other Intentional self-harm with the slope of each line corresponding to estimated MR effect (IVW, WM, and MR-E methods); B, Forest plot of individual and combined SNP MR-estimated effects sizes for relative suicide or other Intentional self-harm; C, The leave-one-out plot visualized how the causal estimates (point with horizontal line) for the effect of morning diurnal preference on suicide or other Intentional self-harm were influenced by the removal of single variant; D, Funnel plot assessing heterogeneity. Blue line represents the inverse-variance weighted estimate, and dark blue line represents the MR-Egger estimate.

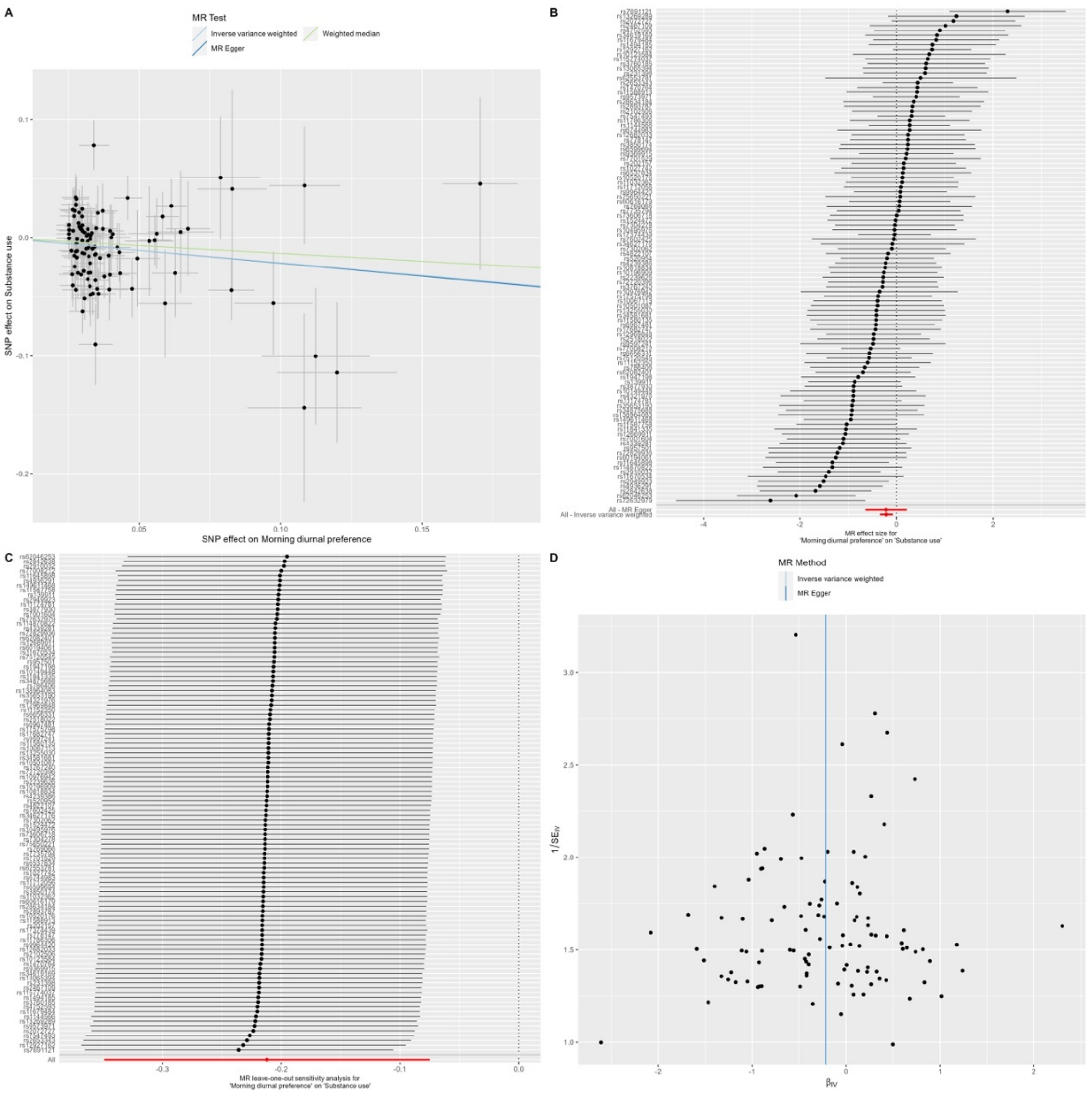

Supplementary Figure24. Mendelian randomization plots for the relationship of morning diurnal preference with substance use

Note: A, Scatterplot of SNP effects on substance use with the slope of each line corresponding to estimated MR effect (IVW, WM, and MR-E methods); B, Forest plot of individual and combined SNP MR-estimated effects sizes for relative substance use; C, The leave-one-out plot visualized how the causal estimates (point with horizontal line) for the effect of morning diurnal preference on substance use were influenced by the removal of single variant; D, Funnel plot assessing heterogeneity. Blue line represents the inverse-variance weighted estimate, and dark blue line represents the MR-Egger estimate.

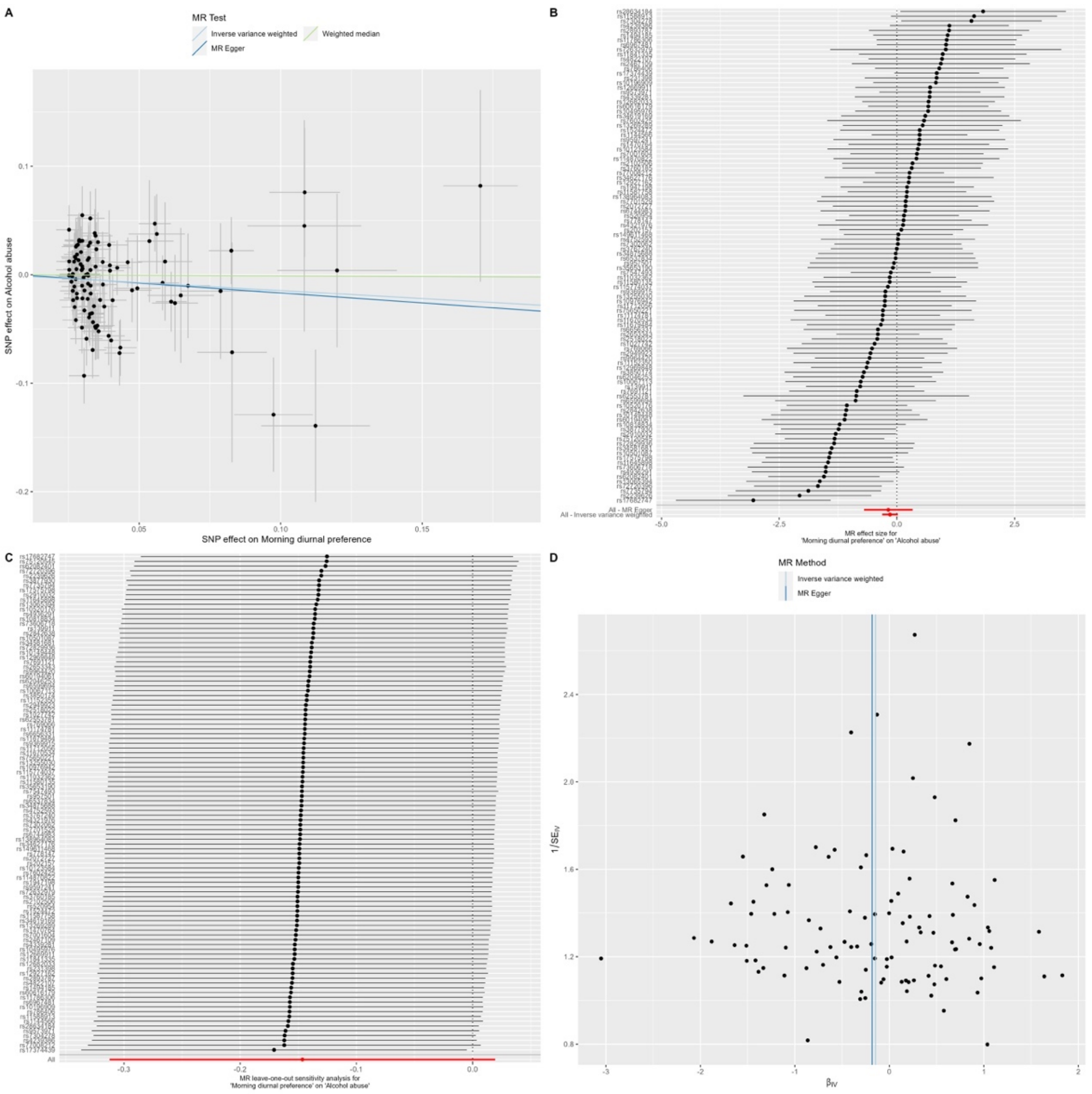

Supplementary Figure25. Mendelian randomization plots for the relationship of morning diurnal preference with alcohol abuse

Note: A, Scatterplot of SNP effects on alcohol abuse with the slope of each line corresponding to estimated MR effect (IVW, WM, and MR-E methods); B, Forest plot of individual and combined SNP MR-estimated effects sizes for relative alcohol abuse; C, The leave-one-out plot visualized how the causal estimates (point with horizontal line) for the effect of morning diurnal preference on alcohol abuse were influenced by the removal of single variant; D, Funnel plot assessing heterogeneity. Blue line represents the inverse-variance weighted estimate, and dark blue line represents the MR-Egger estimate.

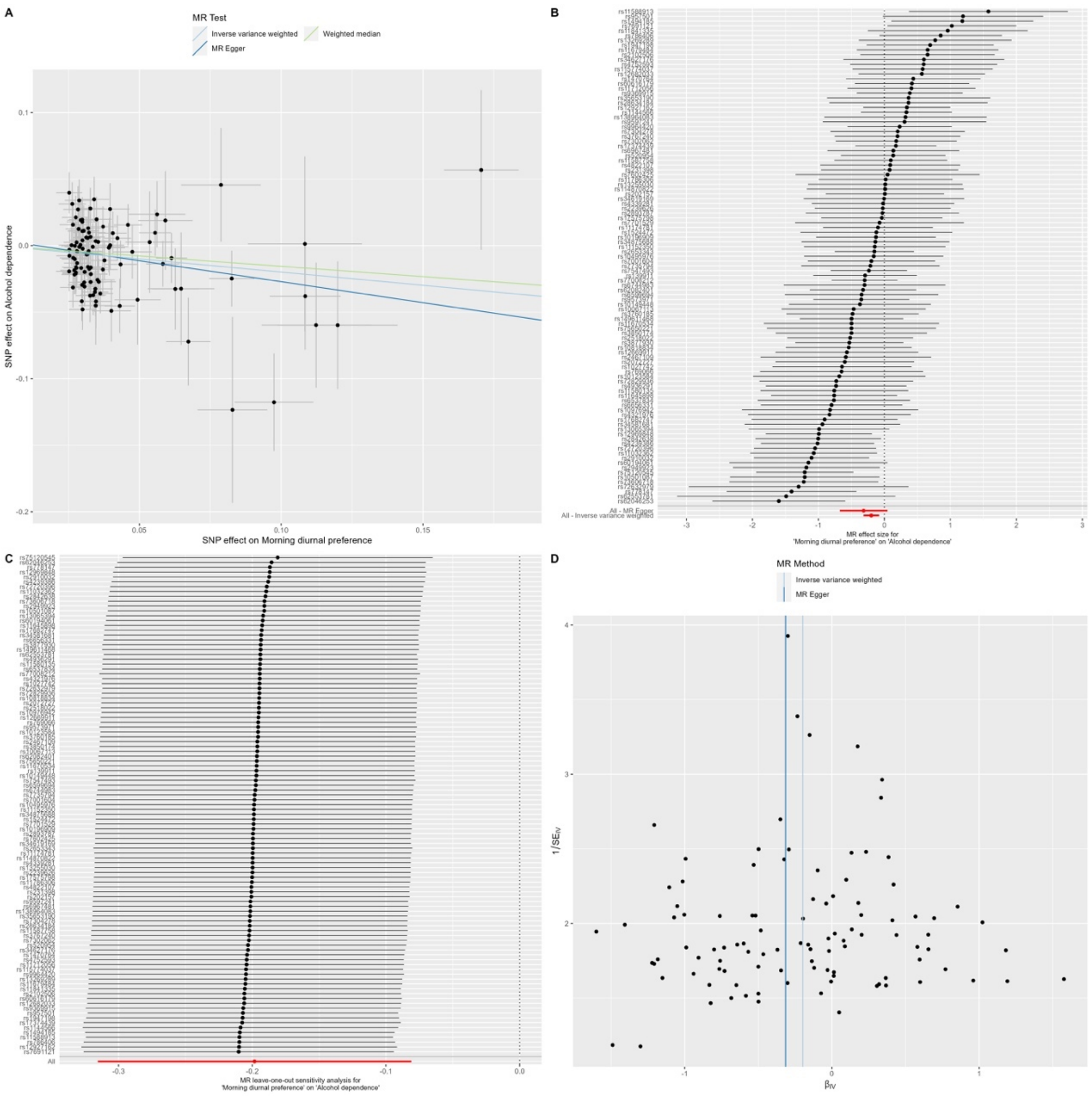

Supplementary Figure26. Mendelian randomization plots for the relationship of morning diurnal preference with alcohol dependence

Note: A, Scatterplot of SNP effects on alcohol dependence with the slope of each line corresponding to estimated MR effect (IVW, WM, and MR-E methods); B, Forest plot of individual and combined SNP MR-estimated effects sizes for relative alcohol dependence; C, The leave-one-out plot visualized how the causal estimates (point with horizontal line) for the effect of morning diurnal preference on alcohol dependence were influenced by the removal of single variant; D, Funnel plot assessing heterogeneity. Blue line represents the inverse-variance weighted estimate, and dark blue line represents the MR-Egger estimate.

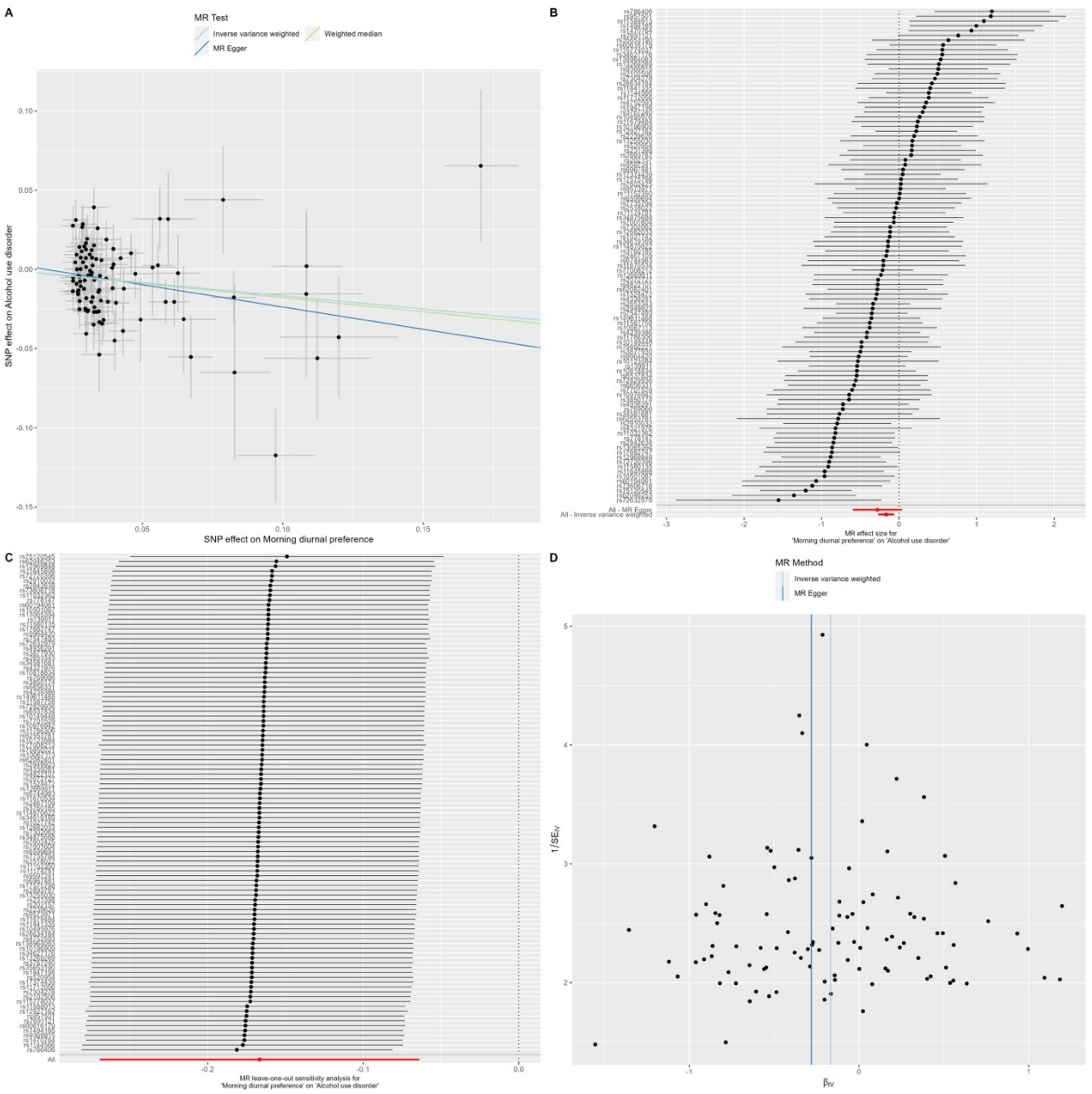

Supplementary Figure27. Mendelian randomization plots for the relationship of morning diurnal preference with alcohol use disorder

Note: A, Scatterplot of SNP effects on alcohol use disorder with the slope of each line corresponding to estimated MR effect (IVW, WM, and MR-E methods); B, Forest plot of individual and combined SNP MR-estimated effects sizes for relative alcohol use disorder; C, The leave-one-out plot visualized how the causal estimates (point with horizontal line) for the effect of morning diurnal preference on alcohol use disorder were influenced by the removal of single variant; D, Funnel plot assessing heterogeneity. Blue line represents the inverse-variance weighted estimate, and dark blue line represents the MR-Egger estimate.

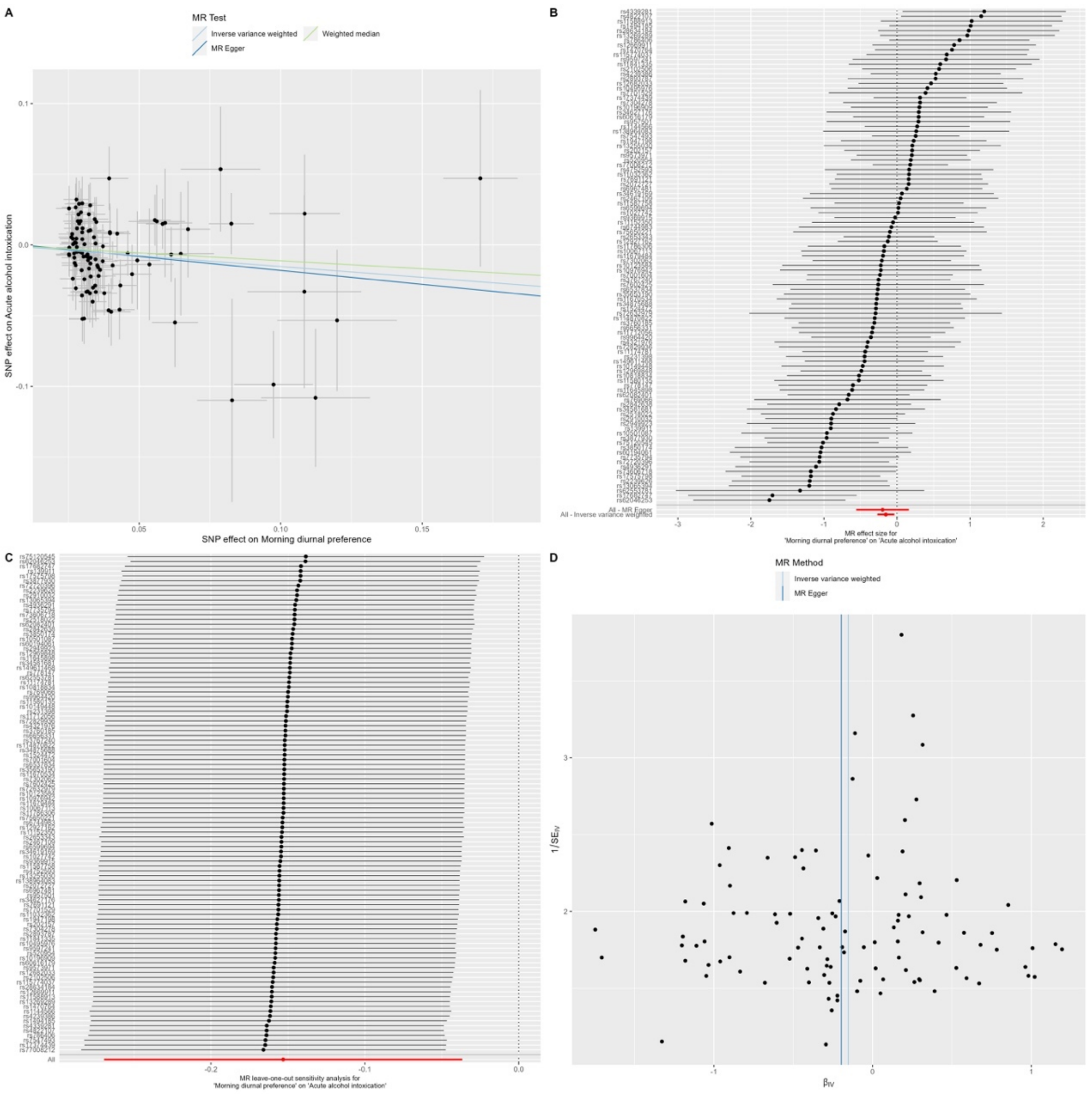

Supplementary Figure28. Mendelian randomization plots for the relationship of morning diurnal preference with acute alcohol intoxication

Note: A, Scatterplot of SNP effects on acute alcohol intoxication with the slope of each line corresponding to estimated MR effect (IVW, WM, and MR-E methods); B, Forest plot of individual and combined SNP MR-estimated effects sizes for relative acute alcohol intoxication; C, The leave-one-out plot visualized how the causal estimates (point with horizontal line) for the effect of morning diurnal preference on acute alcohol intoxication were influenced by the removal of single variant; D, Funnel plot assessing heterogeneity. Blue line represents the inverse-variance weighted estimate, and dark blue line represents the MR-Egger estimate.

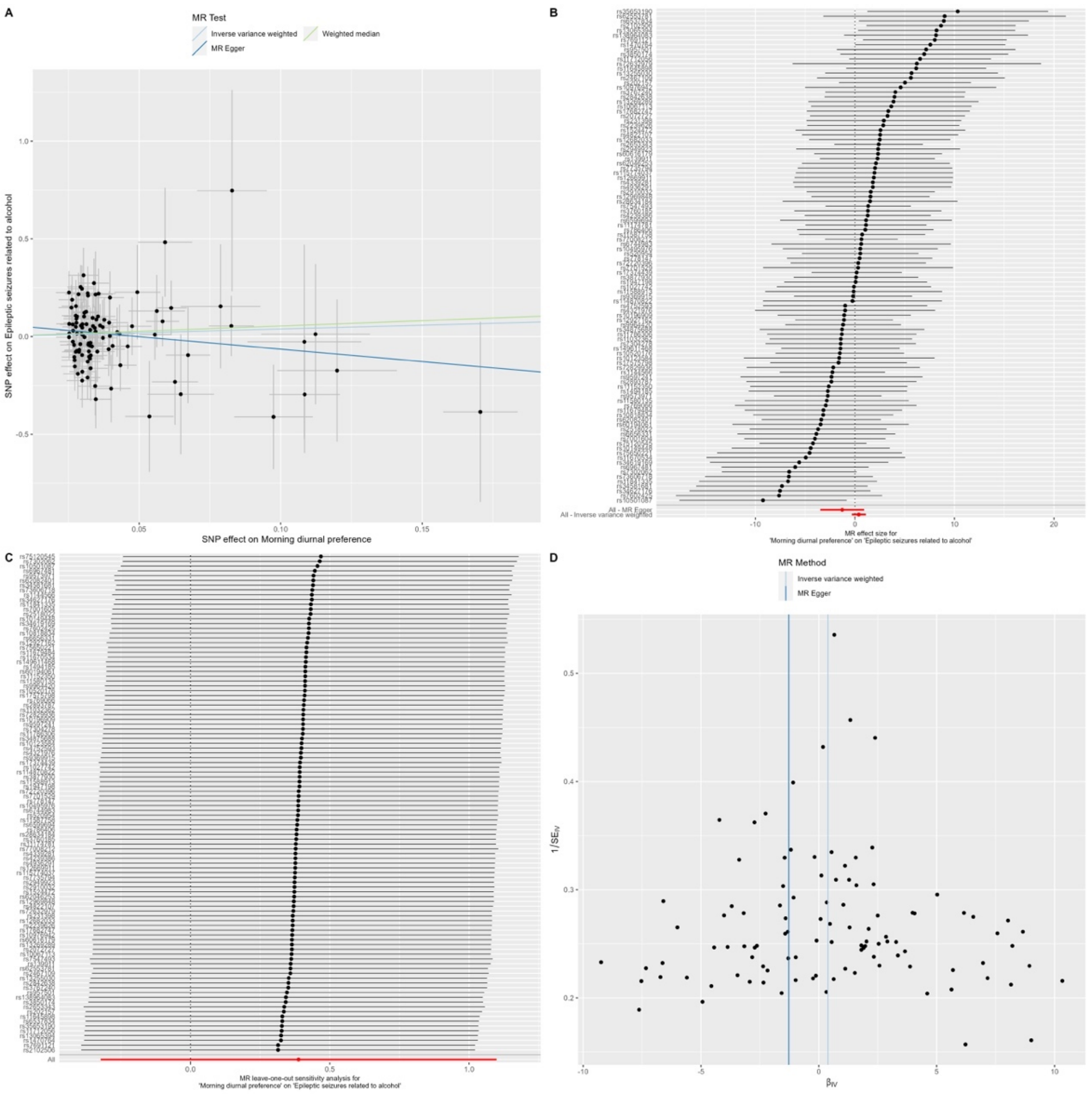

Supplementary Figure29. Mendelian randomization plots for the relationship of morning diurnal preference with epileptic seizures related to alcohol

Note: A, Scatterplot of SNP effects on epileptic seizures related to alcohol with the slope of each line corresponding to estimated MR effect (IVW, WM, and MR-E methods); B, Forest plot of individual and combined SNP MR-estimated effects sizes for relative epileptic seizures related to alcohol; C, The leave-one-out plot visualized how the causal estimates (point with horizontal line) for the effect of morning diurnal preference on epileptic seizures related to alcohol were influenced by the removal of single variant; D, Funnel plot assessing heterogeneity. Blue line represents the inverse-variance weighted estimate, and dark blue line represents the MR-Egger estimate.

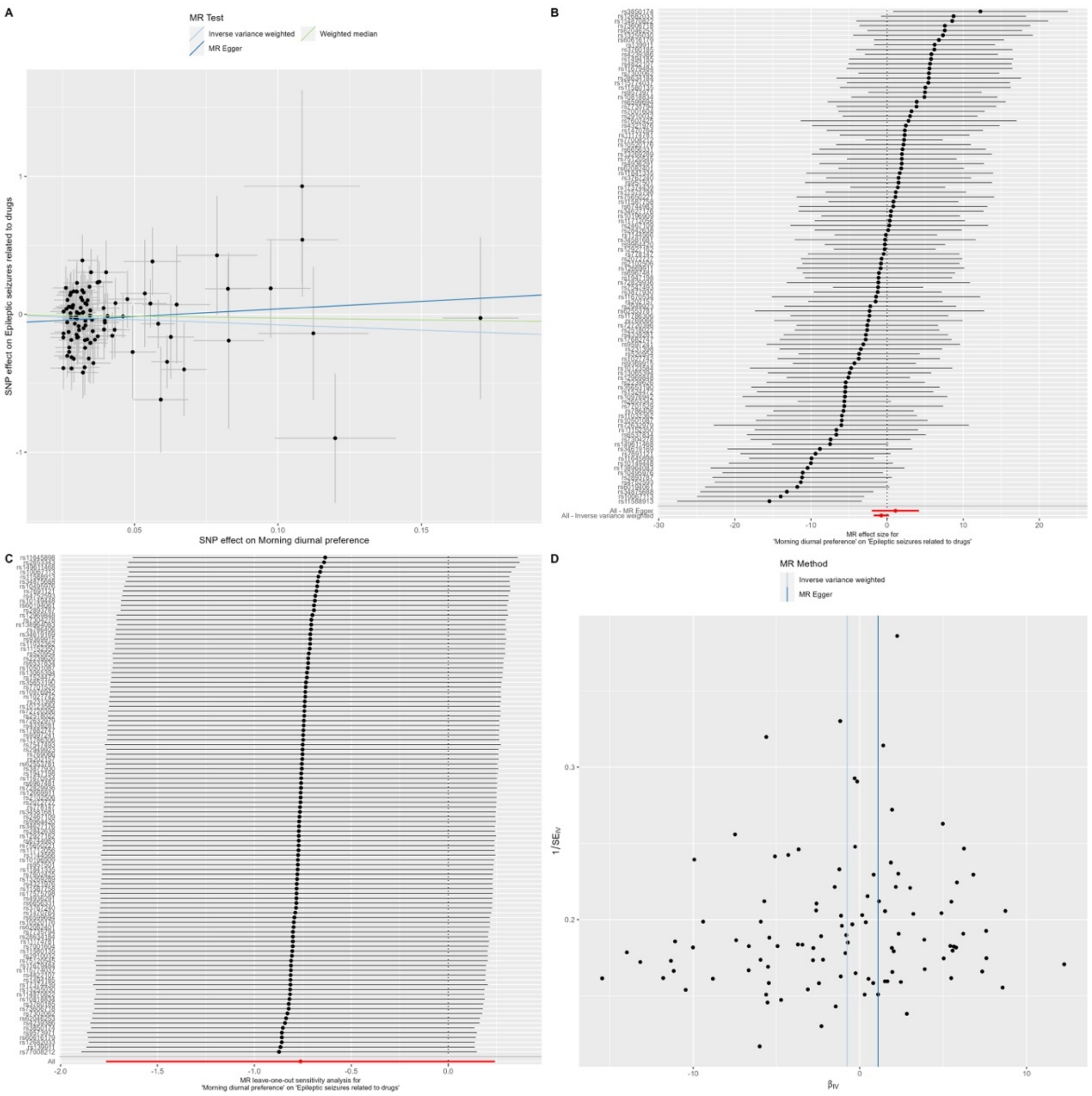

Supplementary Figure30. Mendelian randomization plots for the relationship of morning diurnal preference with epileptic seizures related to drugs

Note: A, Scatterplot of SNP effects on epileptic seizures related to drugs with the slope of each line corresponding to estimated MR effect (IVW, WM, and MR-E methods); B, Forest plot of individual and combined SNP MR-estimated effects sizes for relative epileptic seizures related to drugs; C, The leave-one-out plot visualized how the causal estimates (point with horizontal line) for the effect of morning diurnal preference on epileptic seizures related to drugs were influenced by the removal of single variant; D, Funnel plot assessing heterogeneity. Blue line represents the inverse-variance weighted estimate, and dark blue line represents the MR-Egger estimate.

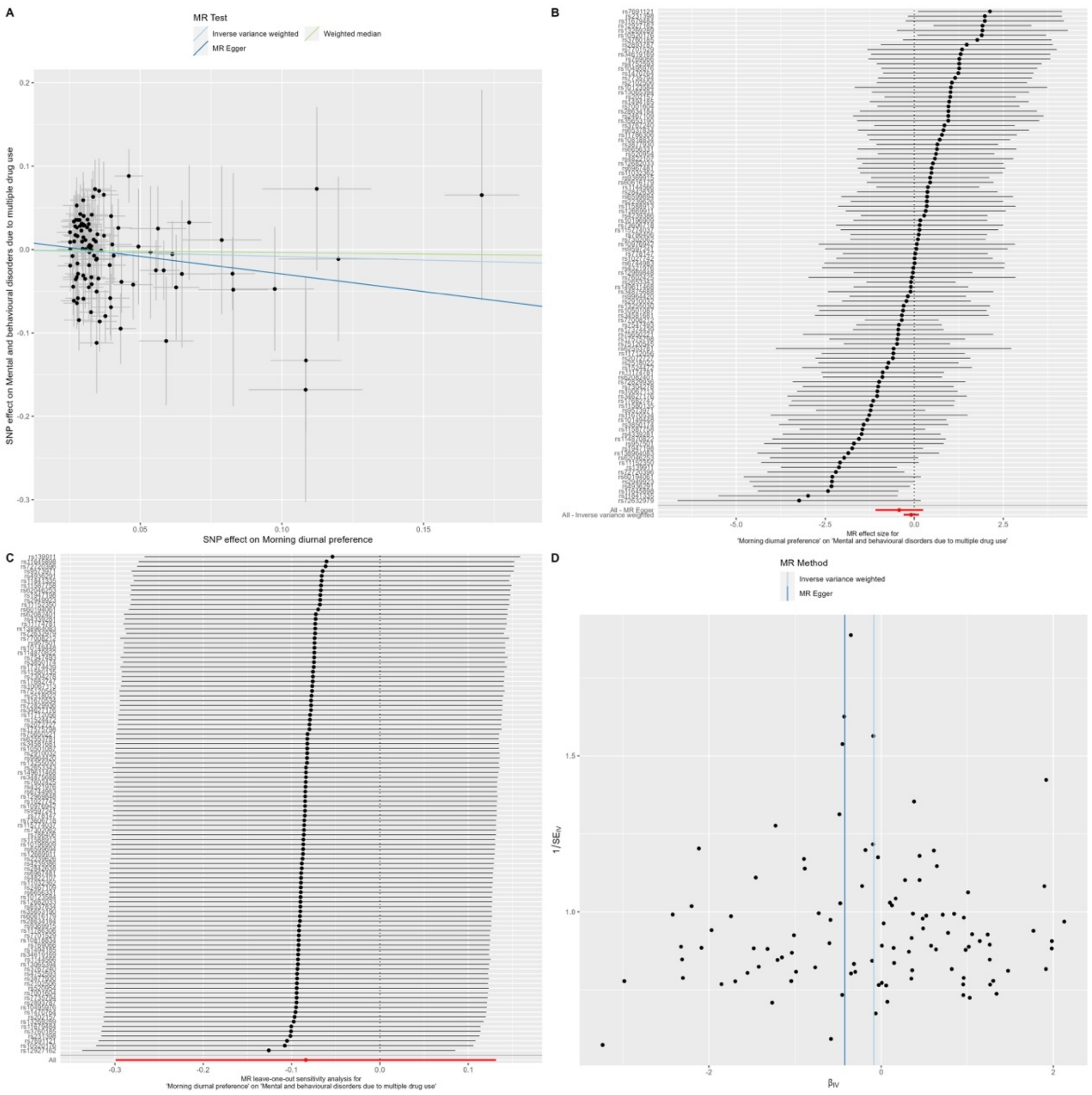

Supplementary Figure31. Mendelian randomization plots for the relationship of morning diurnal preference with mental and behavioural disorders due to multiple drug use

Note: A, Scatterplot of SNP effects on mental and behavioural disorders due to multiple drug use with the slope of each line corresponding to estimated MR effect (IVW, WM, and MR-E methods); B, Forest plot of individual and combined SNP MR-estimated effects sizes for relative mental and behavioural disorders due to multiple drug use; C, The leave-one-out plot visualized how the causal estimates (point with horizontal line) for the effect of morning diurnal preference on mental and behavioural disorders due to multiple drug use were influenced by the removal of single variant; D, Funnel plot assessing heterogeneity. Blue line represents the inverse-variance weighted estimate, and dark blue line represents the MR-Egger estimate.

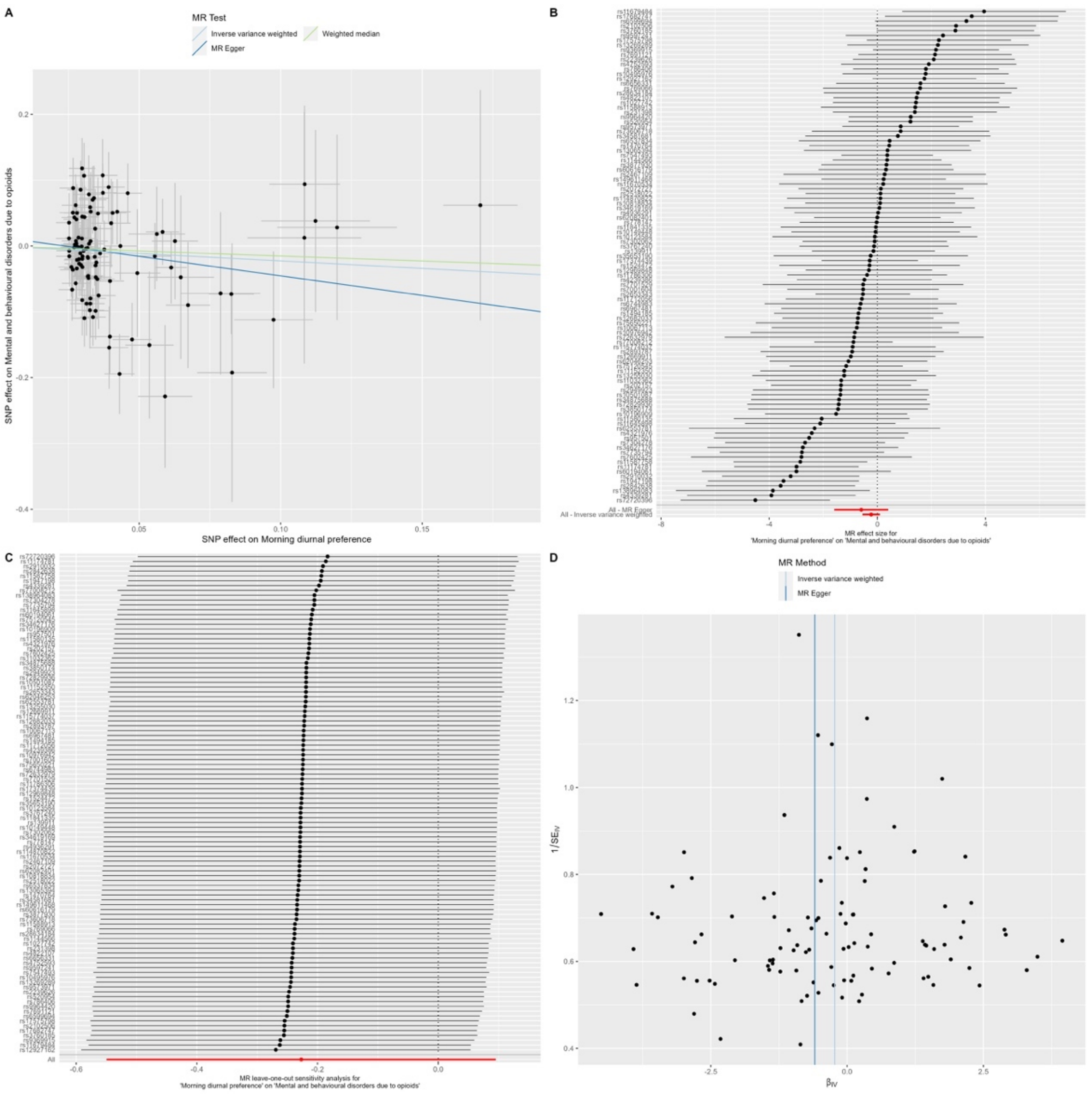

Supplementary Figure32. Mendelian randomization plots for the relationship of morning diurnal preference with mental and behavioural disorders due to opioids

Note: A, Scatterplot of SNP effects on mental and behavioural disorders due to opioids with the slope of each line corresponding to estimated MR effect (IVW, WM, and MR-E methods); B, Forest plot of individual and combined SNP MR-estimated effects sizes for relative mental and behavioural disorders due to opioids; C, The leave-one-out plot visualized how the causal estimates (point with horizontal line) for the effect of morning diurnal preference on mental and behavioural disorders due to opioids were influenced by the removal of single variant; D, Funnel plot assessing heterogeneity. Blue line represents the inverse-variance weighted estimate, and dark blue line represents the MR-Egger estimate.

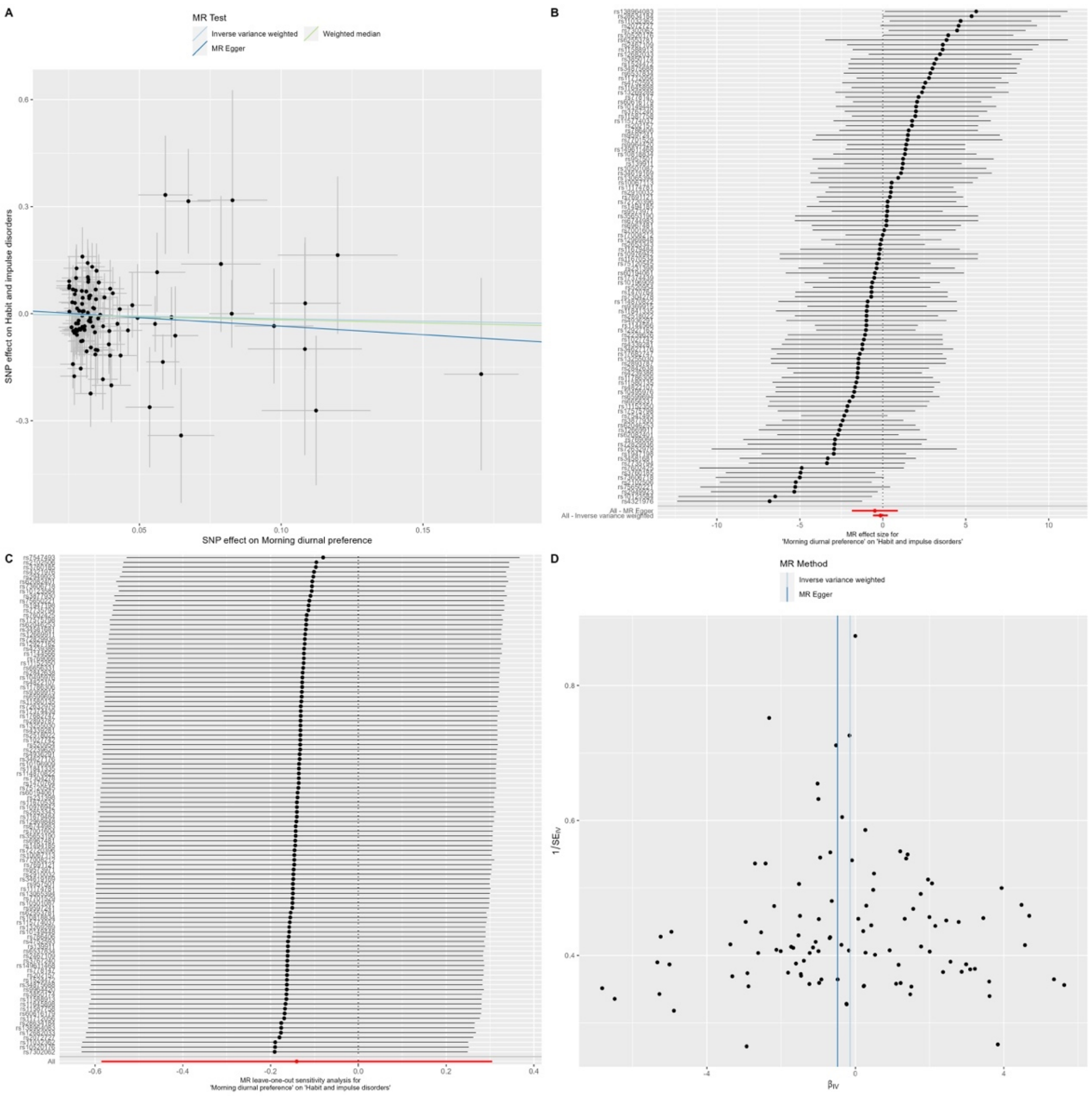

Supplementary Figure33. Mendelian randomization plots for the relationship of morning diurnal preference with habit and impulse disorders

Note: A, Scatterplot of SNP effects on habit and impulse disorders with the slope of each line corresponding to estimated MR effect (IVW, WM, and MR-E methods); B, Forest plot of individual and combined SNP MR-estimated effects sizes for relative habit and impulse disorders; C, The leave-one-out plot visualized how the causal estimates (point with horizontal line) for the effect of morning diurnal preference on habit and impulse disorders were influenced by the removal of single variant; D, Funnel plot assessing heterogeneity. Blue line represents the inverse-variance weighted estimate, and dark blue line represents the MR-Egger estimate.

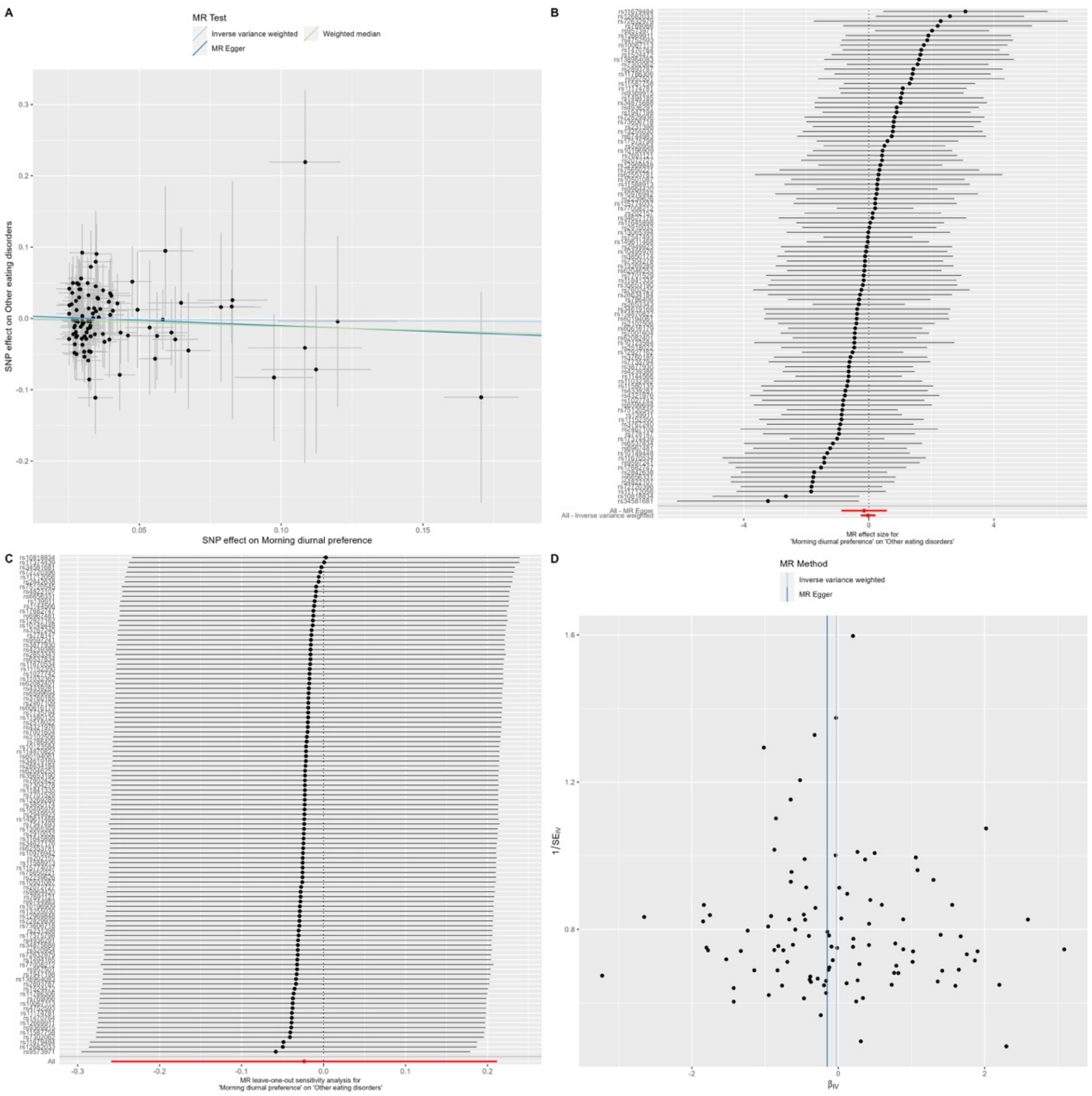

Supplementary Figure34. Mendelian randomization plots for the relationship of morning diurnal preference with other eating disorders

Note: A, Scatterplot of SNP effects on other eating disorders with the slope of each line corresponding to estimated MR effect (IVW, WM, and MR-E methods); B, Forest plot of individual and combined SNP MR-estimated effects sizes for relative other eating disorders; C, The leave-one-out plot visualized how the causal estimates (point with horizontal line) for the effect of morning diurnal preference on other eating disorders were influenced by the removal of single variant; D, Funnel plot assessing heterogeneity. Blue line represents the inverse-variance weighted estimate, and dark blue line represents the MR-Egger estimate.

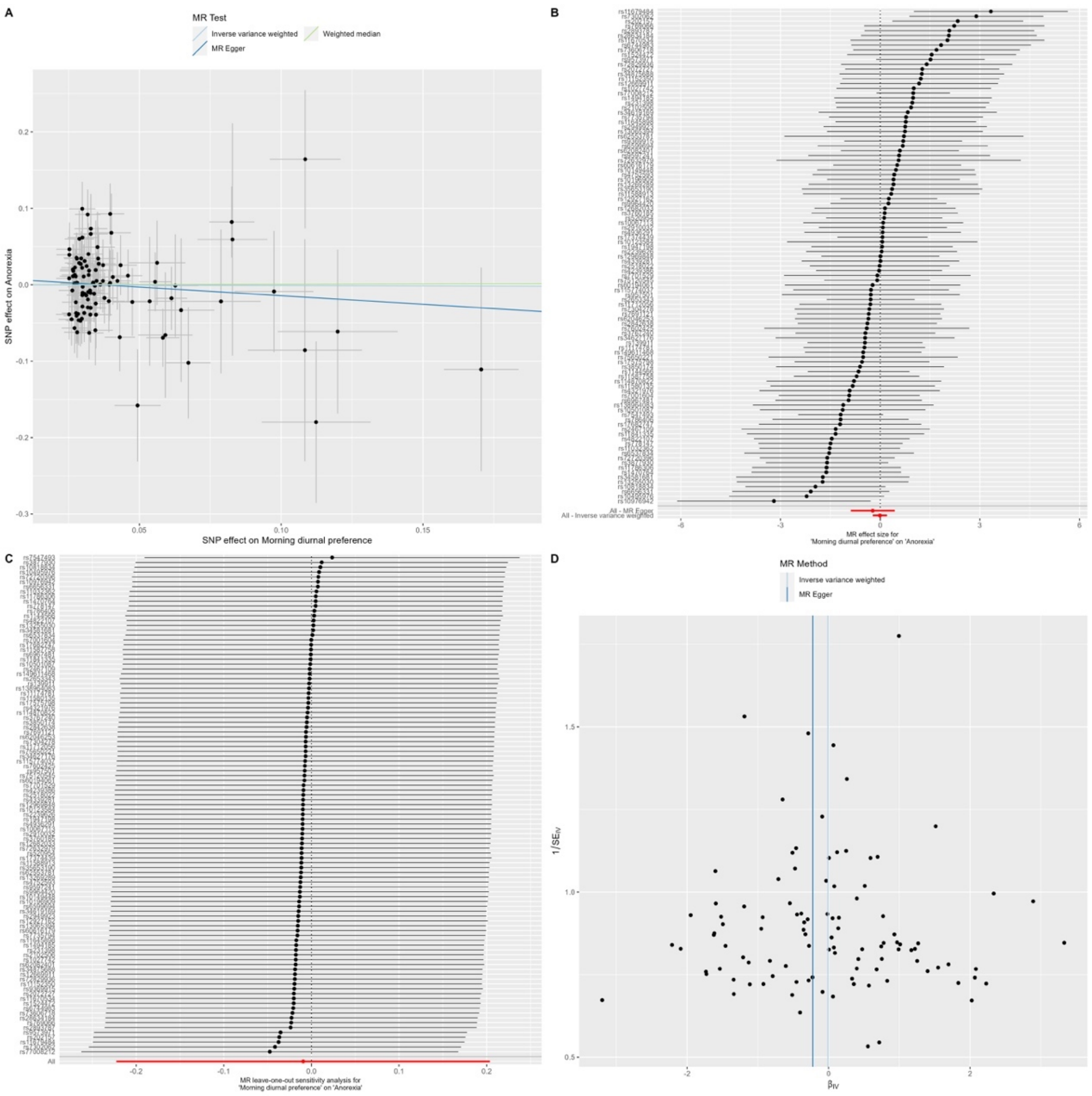

Supplementary Figure35. Mendelian randomization plots for the relationship of morning diurnal preference with anorexia

Note: A, Scatterplot of SNP effects on anorexia with the slope of each line corresponding to estimated MR effect (IVW, WM, and MR-E methods); B, Forest plot of individual and combined SNP MR-estimated effects sizes for relative anorexia; C, The leave-one-out plot visualized how the causal estimates (point with horizontal line) for the effect of morning diurnal preference on anorexia were influenced by the removal of single variant; D, Funnel plot assessing heterogeneity. Blue line represents the inverse-variance weighted estimate, and dark blue line represents the MR-Egger estimate.

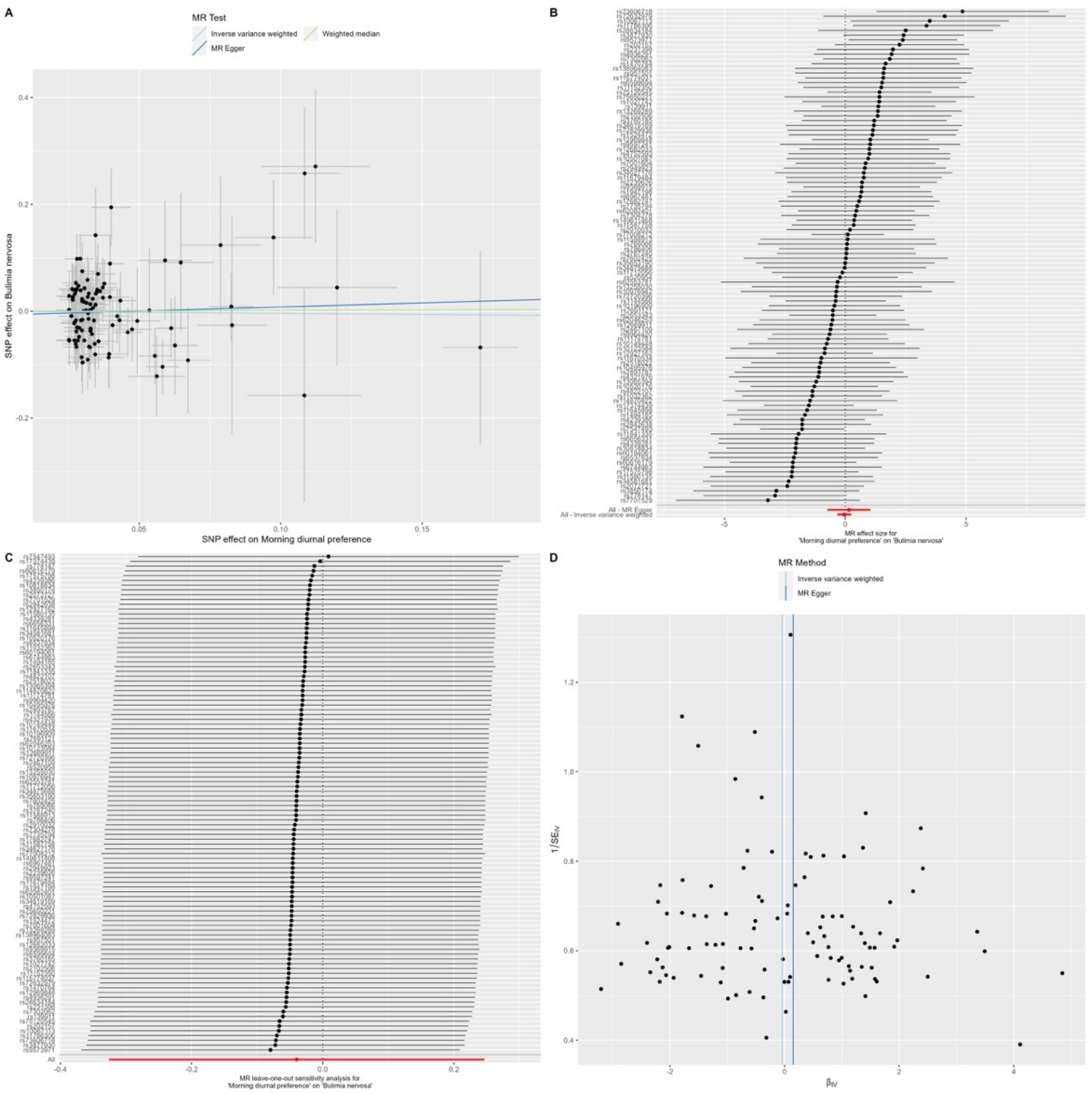

Supplementary Figure36. Mendelian randomization plots for the relationship of morning diurnal preference with bulimia nervosa

Note: A, Scatterplot of SNP effects on bulimia nervosa with the slope of each line corresponding to estimated MR effect (IVW, WM, and MR-E methods); B, Forest plot of individual and combined SNP MR-estimated effects sizes for relative bulimia nervosa; C, The leave-one-out plot visualized how the causal estimates (point with horizontal line) for the effect of morning diurnal preference on bulimia nervosa were influenced by the removal of single variant; D, Funnel plot assessing heterogeneity. Blue line represents the inverse-variance weighted estimate, and dark blue line represents the MR-Egger estimate.

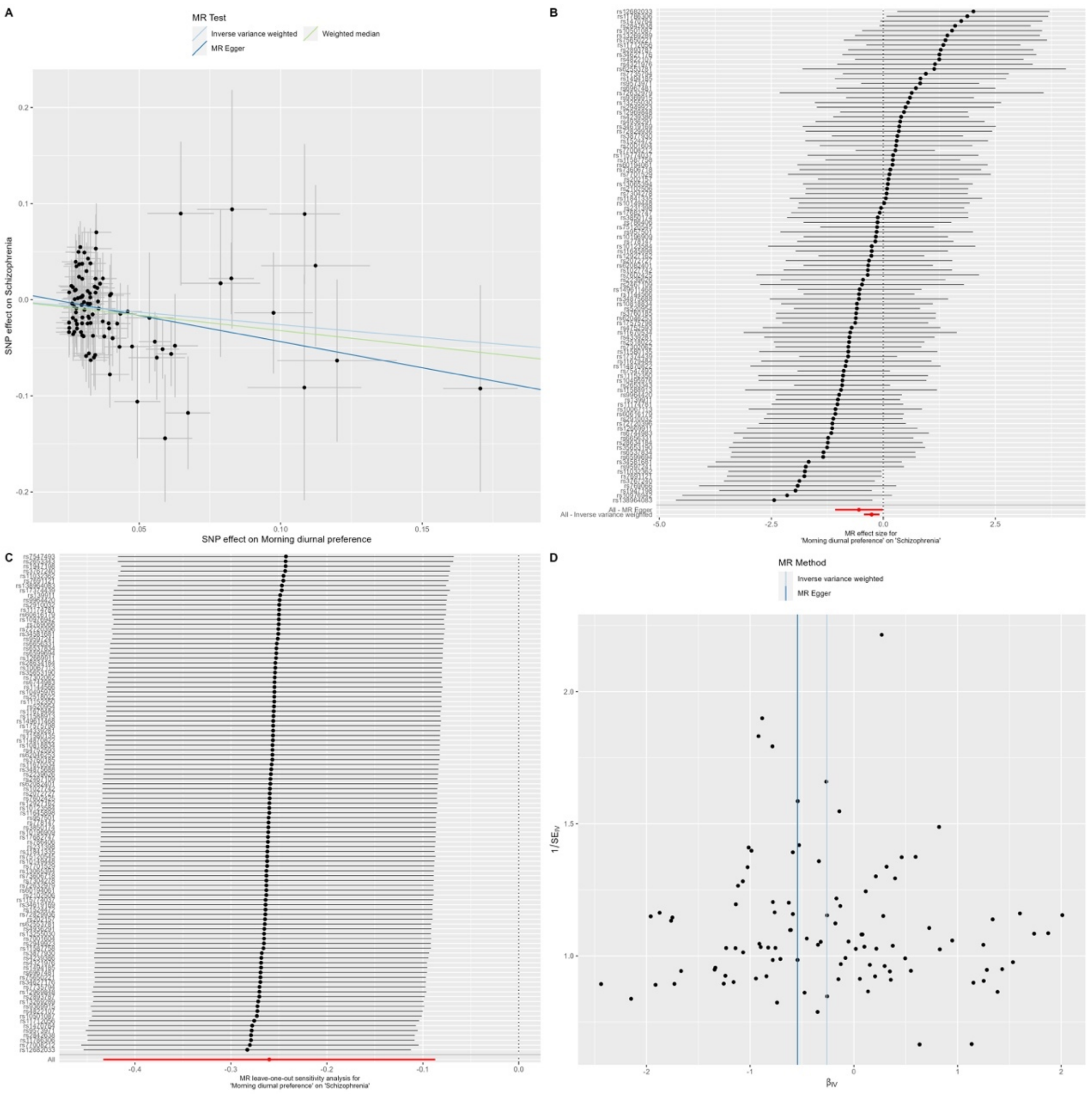

Supplementary Figure37. Mendelian randomization plots for the relationship of morning diurnal preference with schizophrenia

Note: A, Scatterplot of SNP effects on schizophrenia with the slope of each line corresponding to estimated MR effect (IVW, WM, and MR-E methods); B, Forest plot of individual and combined SNP MR-estimated effects sizes for relative schizophrenia; C, The leave-one-out plot visualized how the causal estimates (point with horizontal line) for the effect of morning diurnal preference on schizophrenia were influenced by the removal of single variant; D, Funnel plot assessing heterogeneity. Blue line represents the inverse-variance weighted estimate, and dark blue line represents the MR-Egger estimate.

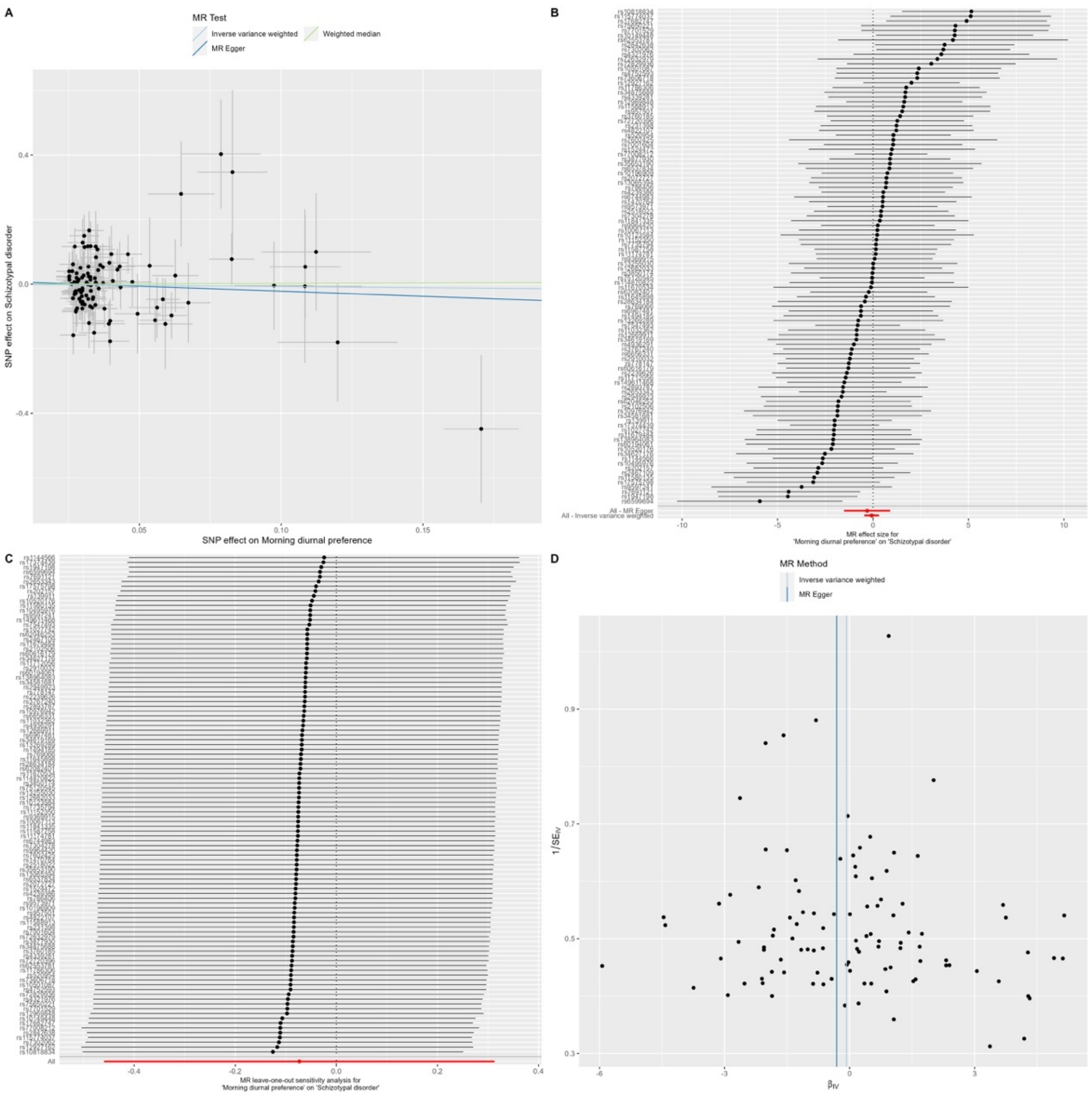

Supplementary Figure38. Mendelian randomization plots for the relationship of morning diurnal preference with schizotypal disorder

Note: A, Scatterplot of SNP effects on schizotypal disorder with the slope of each line corresponding to estimated MR effect (IVW, WM, and MR-E methods); B, Forest plot of individual and combined SNP MR-estimated effects sizes for relative schizotypal disorder; C, The leave-one-out plot visualized how the causal estimates (point with horizontal line) for the effect of morning diurnal preference on schizotypal disorder were influenced by the removal of single variant; D, Funnel plot assessing heterogeneity. Blue line represents the inverse-variance weighted estimate, and dark blue line represents the MR-Egger estimate.

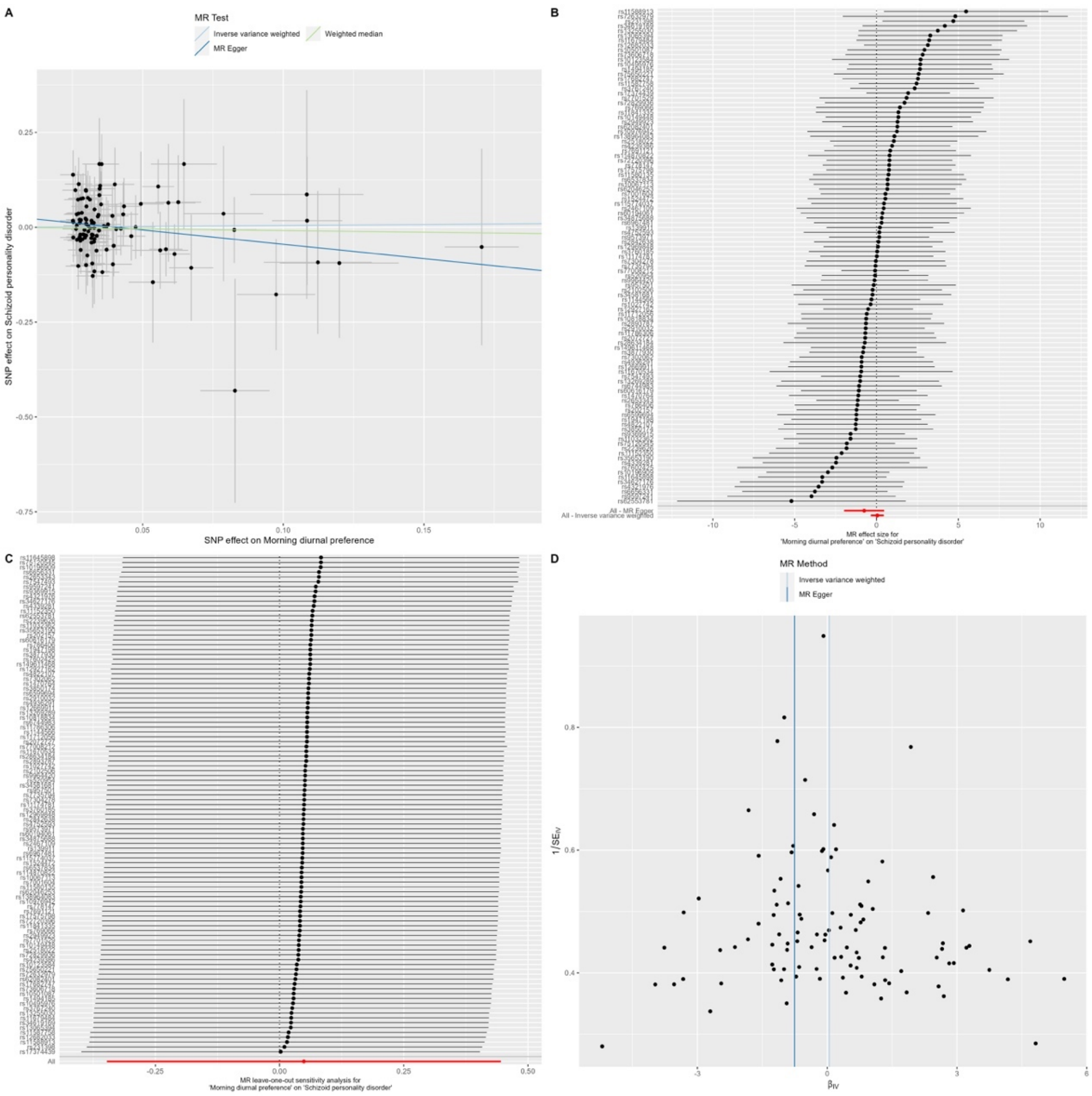

Supplementary Figure39. Mendelian randomization plots for the relationship of morning diurnal preference with schizoid personality disorder

Note: A, Scatterplot of SNP effects on schizoid personality disorder with the slope of each line corresponding to estimated MR effect (IVW, WM, and MR-E methods); B, Forest plot of individual and combined SNP MR-estimated effects sizes for relative schizoid personality disorder; C, The leave-one-out plot visualized how the causal estimates (point with horizontal line) for the effect of morning diurnal preference on schizoid personality disorder were influenced by the removal of single variant; D, Funnel plot assessing heterogeneity. Blue line represents the inverse-variance weighted estimate, and dark blue line represents the MR-Egger estimate.

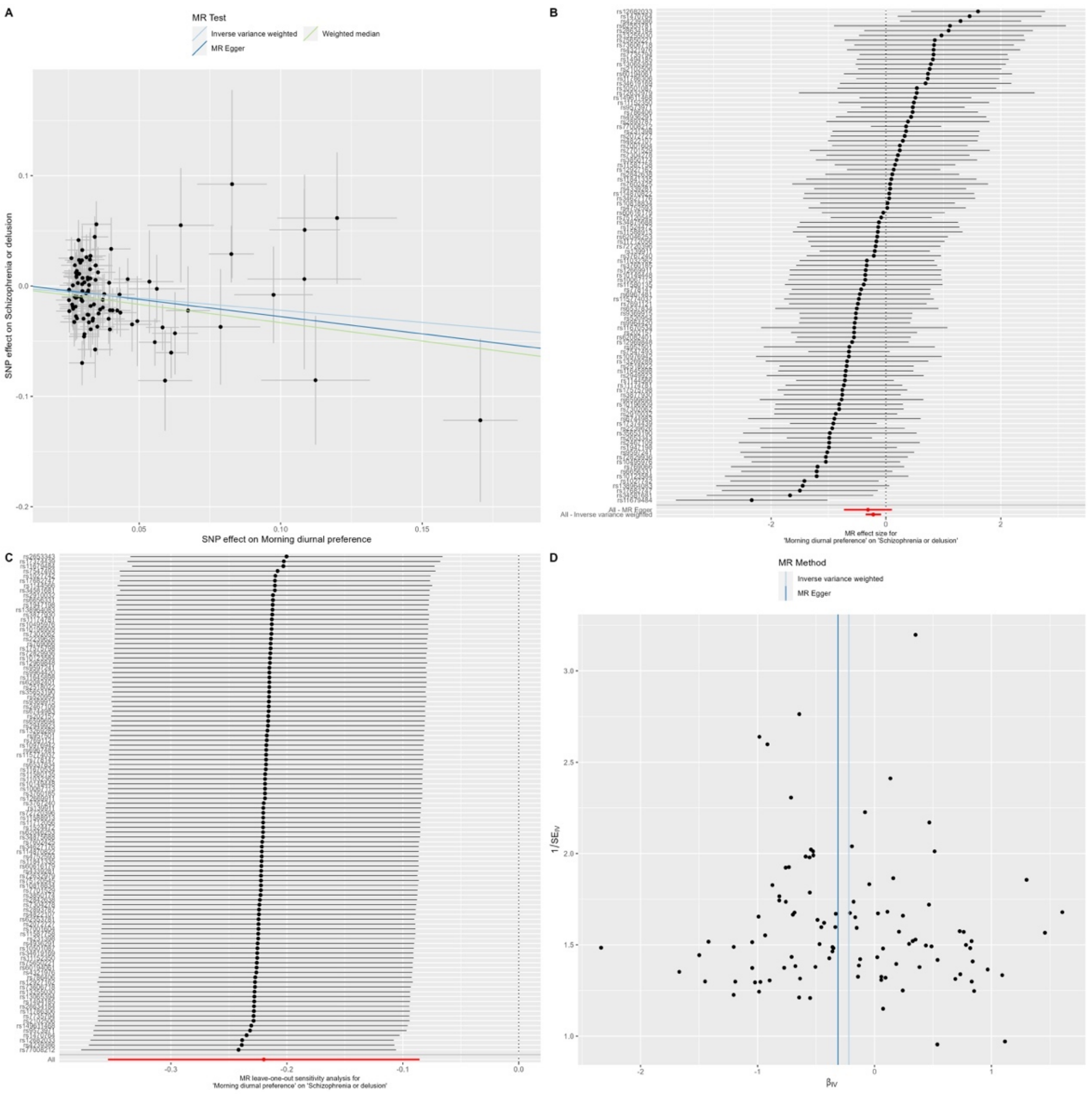

Supplementary Figure40. Mendelian randomization plots for the relationship of morning diurnal preference with schizophrenia or delusion

Note: A, Scatterplot of SNP effects on schizophrenia or delusion with the slope of each line corresponding to estimated MR effect (IVW, WM, and MR-E methods); B, Forest plot of individual and combined SNP MR-estimated effects sizes for relative schizophrenia or delusion; C, The leave-one-out plot visualized how the causal estimates (point with horizontal line) for the effect of morning diurnal preference on schizophrenia or delusion were influenced by the removal of single variant; D, Funnel plot assessing heterogeneity. Blue line represents the inverse-variance weighted estimate, and dark blue line represents the MR-Egger estimate.

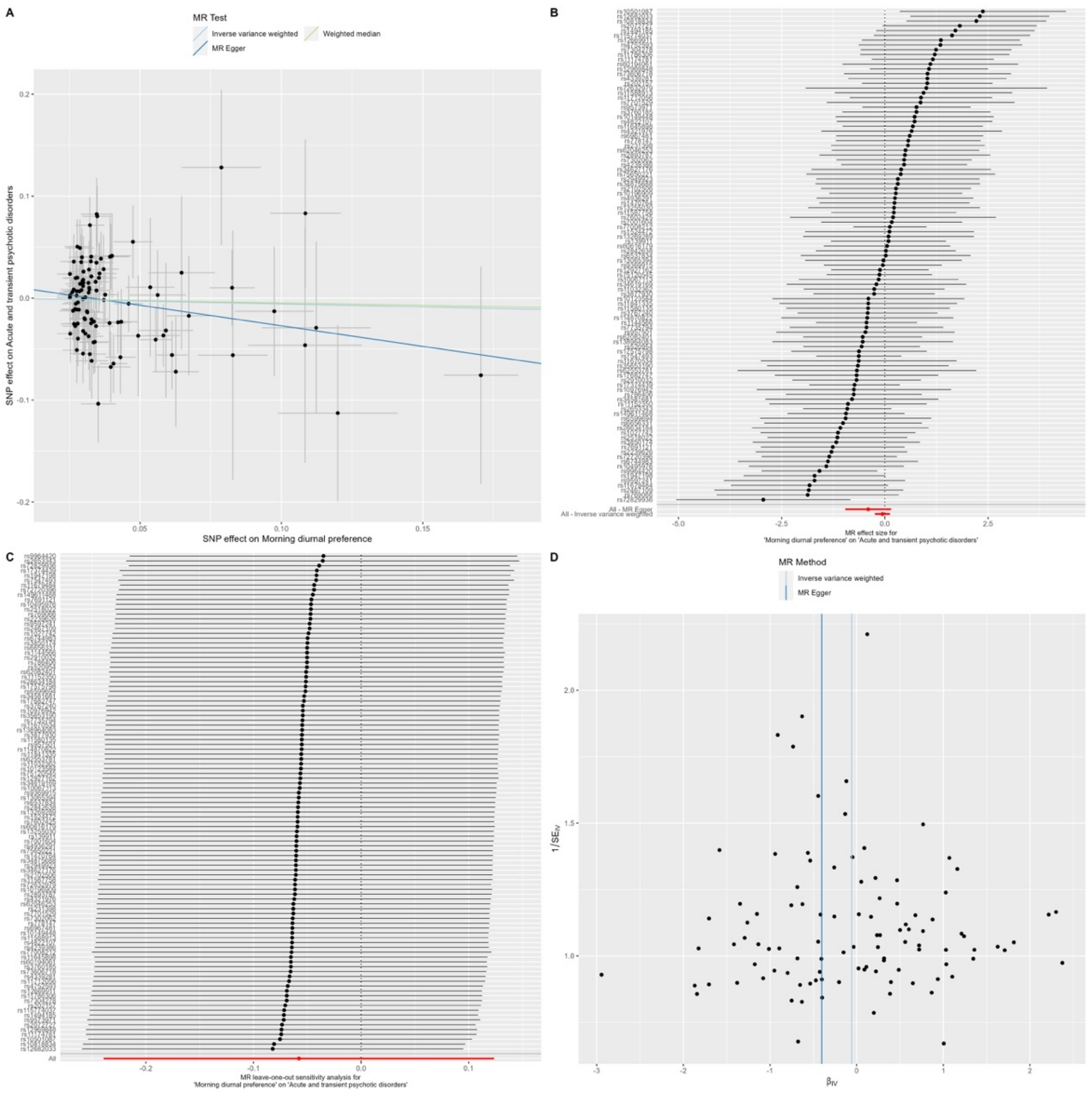

Supplementary Figure41. Mendelian randomization plots for the relationship of morning diurnal preference with acute and transient psychotic disorders

Note: A, Scatterplot of SNP effects on acute and transient psychotic disorders with the slope of each line corresponding to estimated MR effect (IVW, WM, and MR-E methods); B, Forest plot of individual and combined SNP MR-estimated effects sizes for relative acute and transient psychotic disorders; C, The leave-one-out plot visualized how the causal estimates (point with horizontal line) for the effect of morning diurnal preference on acute and transient psychotic disorders were influenced by the removal of single variant; D, Funnel plot assessing heterogeneity. Blue line represents the inverse-variance weighted estimate, and dark blue line represents the MR-Egger estimate.

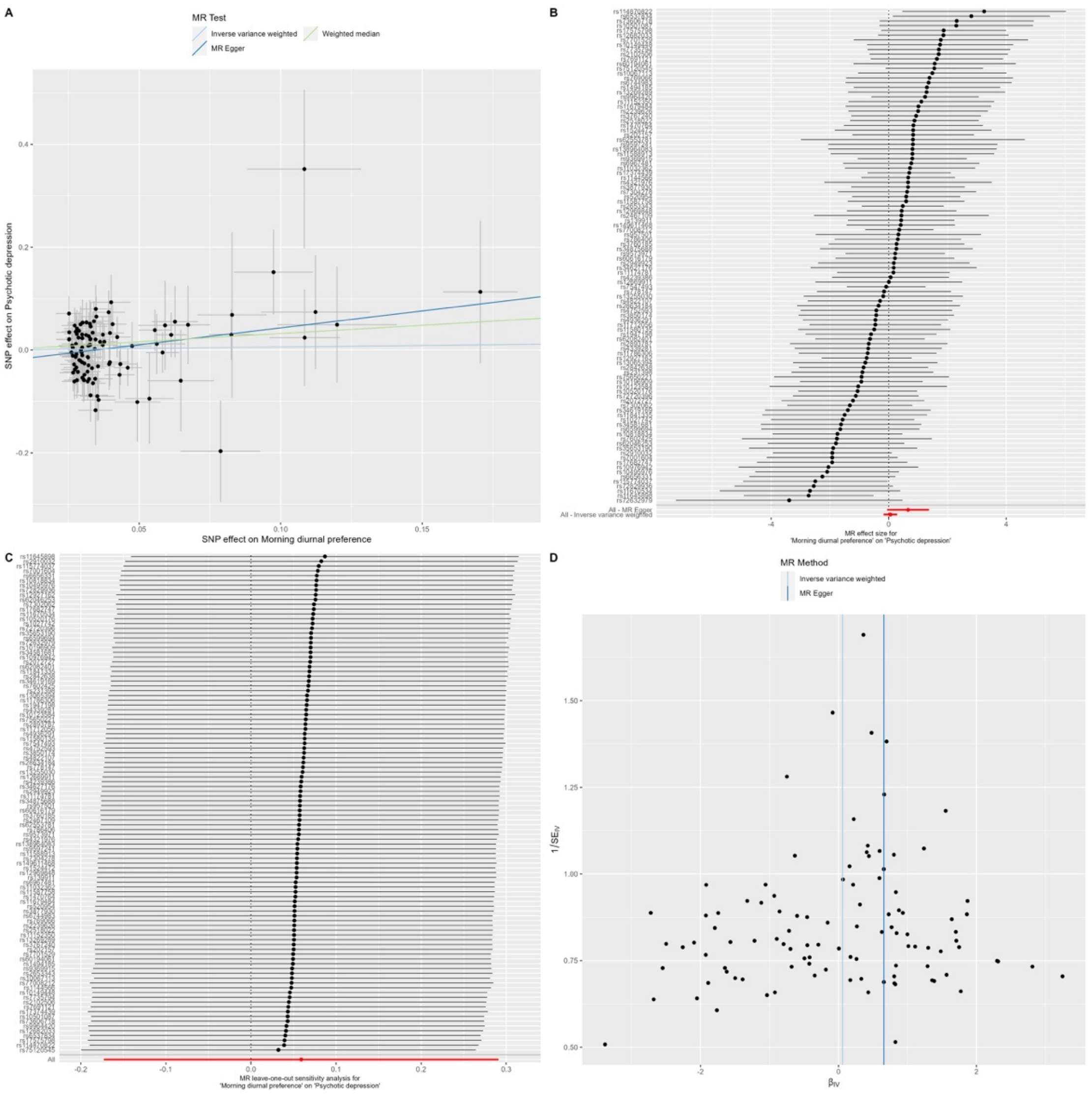

Supplementary Figure42. Mendelian randomization plots for the relationship of morning diurnal preference with psychotic depression

Note: A, Scatterplot of SNP effects on psychotic depression with the slope of each line corresponding to estimated MR effect (IVW, WM, and MR-E methods); B, Forest plot of individual and combined SNP MR-estimated effects sizes for relative psychotic depression; C, The leave-one-out plot visualized how the causal estimates (point with horizontal line) for the effect of morning diurnal preference on psychotic depression were influenced by the removal of single variant; D, Funnel plot assessing heterogeneity. Blue line represents the inverse-variance weighted estimate, and dark blue line represents the MR-Egger estimate.

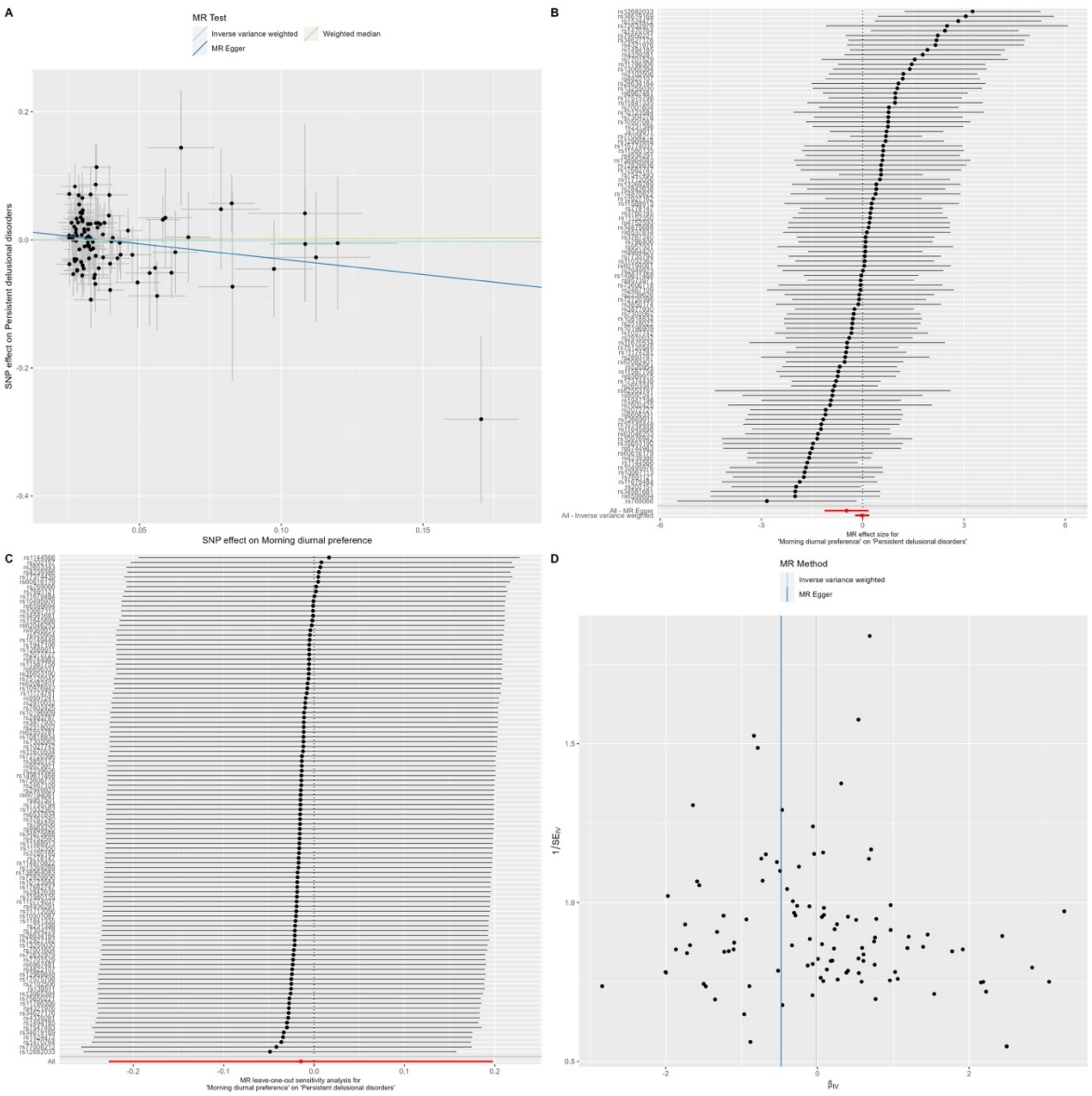

Supplementary Figure43. Mendelian randomization plots for the relationship of morning diurnal preference with persistent delusional disorders

Note: A, Scatterplot of SNP effects on persistent delusional disorders with the slope of each line corresponding to estimated MR effect (IVW, WM, and MR-E methods); B, Forest plot of individual and combined SNP MR-estimated effects sizes for relative persistent delusional disorders; C, The leave-one-out plot visualized how the causal estimates (point with horizontal line) for the effect of morning diurnal preference on persistent delusional disorders were influenced by the removal of single variant; D, Funnel plot assessing heterogeneity. Blue line represents the inverse-variance weighted estimate, and dark blue line represents the MR-Egger estimate.

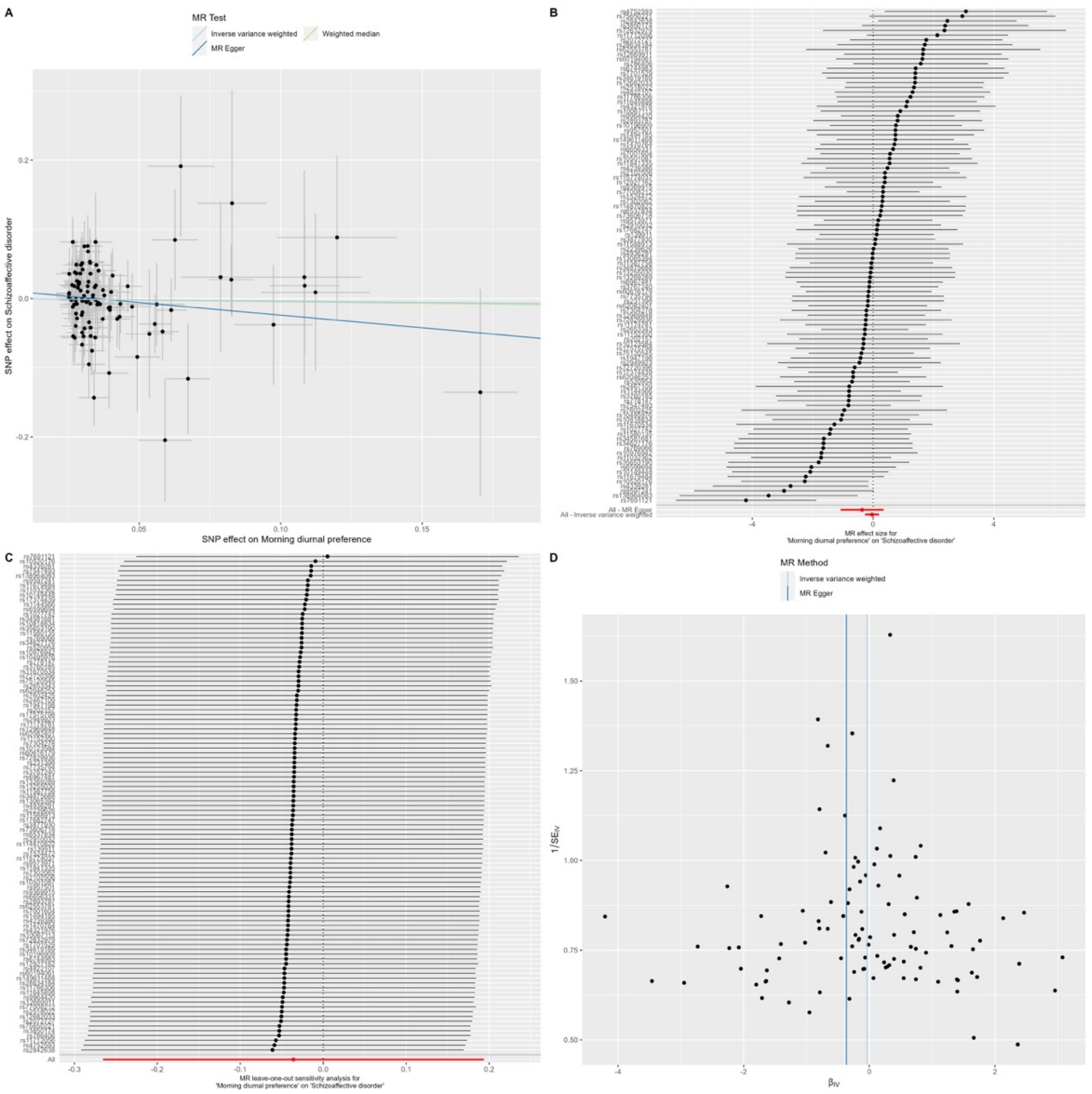

Supplementary Figure44. Mendelian randomization plots for the relationship of morning diurnal preference with schizoaffective disorder

Note: A, Scatterplot of SNP effects on schizoaffective disorder with the slope of each line corresponding to estimated MR effect (IVW, WM, and MR-E methods); B, Forest plot of individual and combined SNP MR-estimated effects sizes for relative schizoaffective disorder; C, The leave-one-out plot visualized how the causal estimates (point with horizontal line) for the effect of morning diurnal preference on schizoaffective disorder were influenced by the removal of single variant; D, Funnel plot assessing heterogeneity. Blue line represents the inverse-variance weighted estimate, and dark blue line represents the MR-Egger estimate.

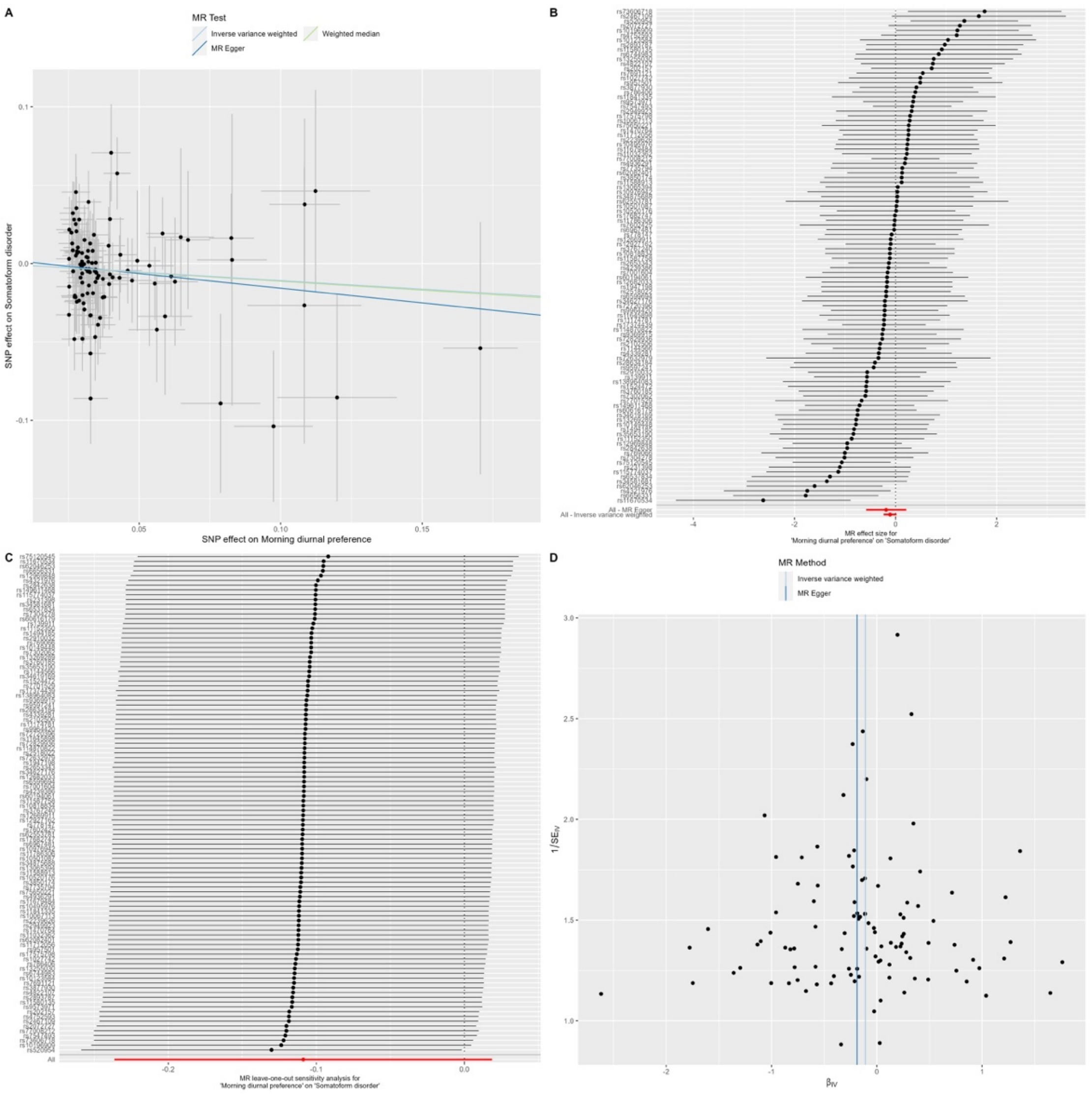

Supplementary Figure45. Mendelian randomization plots for the relationship of morning diurnal preference with somatoform disorder

Note: A, Scatterplot of SNP effects on somatoform disorder with the slope of each line corresponding to estimated MR effect (IVW, WM, and MR-E methods); B, Forest plot of individual and combined SNP MR-estimated effects sizes for relative somatoform disorder; C, The leave-one-out plot visualized how the causal estimates (point with horizontal line) for the effect of morning diurnal preference on somatoform disorder were influenced by the removal of single variant; D, Funnel plot assessing heterogeneity. Blue line represents the inverse-variance weighted estimate, and dark blue line represents the MR-Egger estimate.

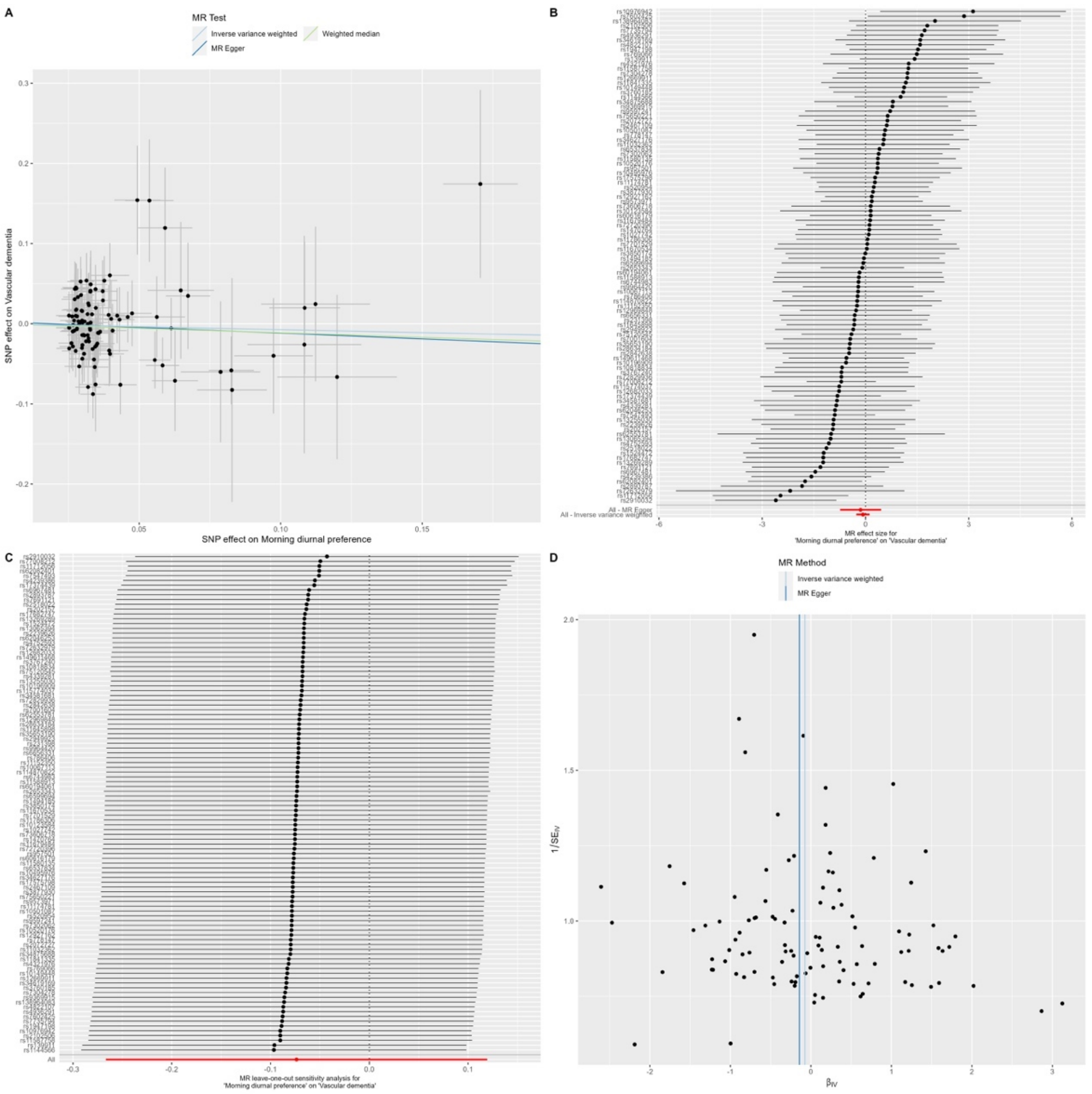

Supplementary Figure46. Mendelian randomization plots for the relationship of morning diurnal preference with vascular dementia

Note: A, Scatterplot of SNP effects on vascular dementia with the slope of each line corresponding to estimated MR effect (IVW, WM, and MR-E methods); B, Forest plot of individual and combined SNP MR-estimated effects sizes for relative vascular dementia; C, The leave-one-out plot visualized how the causal estimates (point with horizontal line) for the effect of morning diurnal preference on vascular dementia were influenced by the removal of single variant; D, Funnel plot assessing heterogeneity. Blue line represents the inverse-variance weighted estimate, and dark blue line represents the MR-Egger estimate.

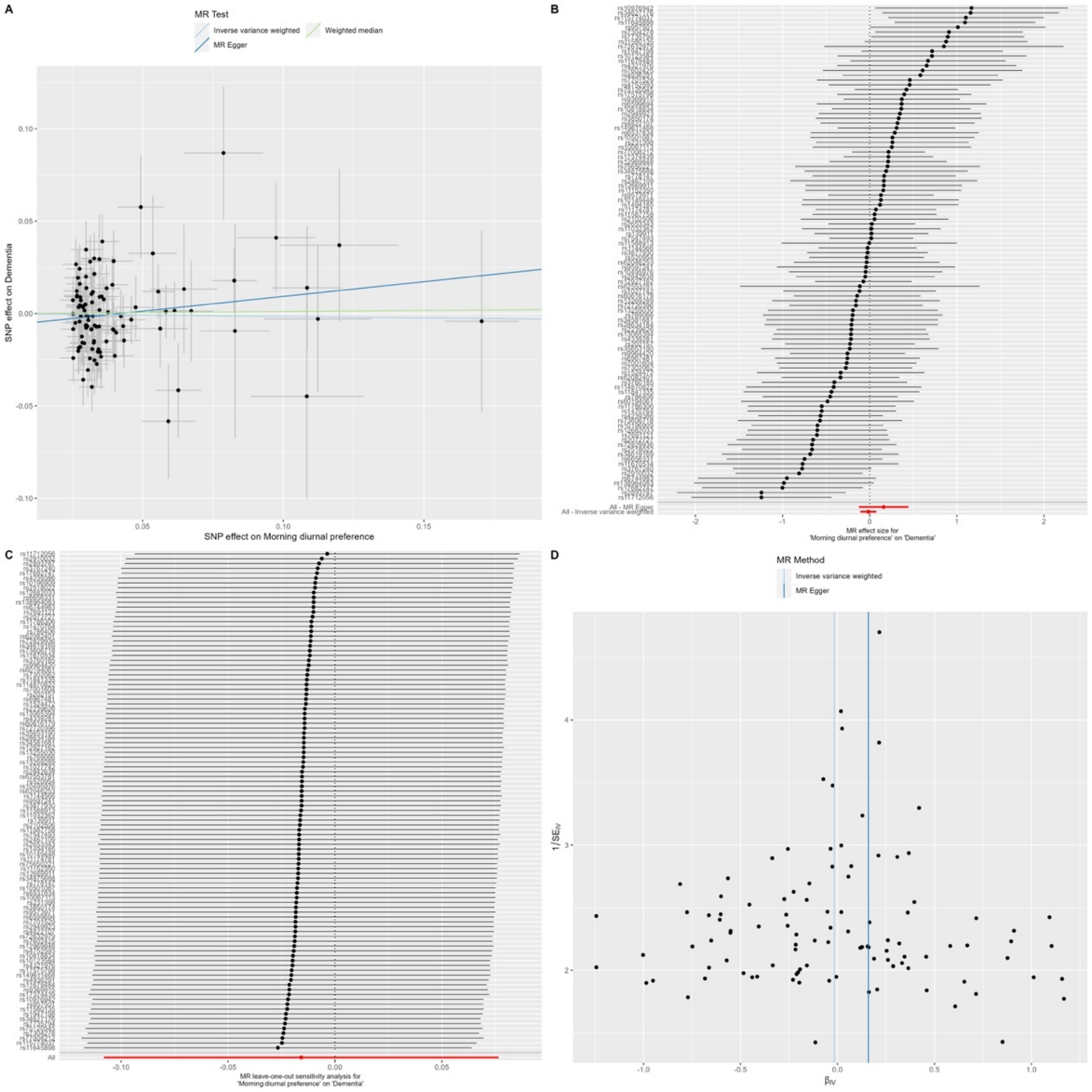

Supplementary Figure47. Mendelian randomization plots for the relationship of morning diurnal preference with dementia

Note: A, Scatterplot of SNP effects on dementia with the slope of each line corresponding to estimated MR effect (IVW, WM, and MR-E methods); B, Forest plot of individual and combined SNP MR-estimated effects sizes for relative dementia; C, The leave-one-out plot visualized how the causal estimates (point with horizontal line) for the effect of morning diurnal preference on dementia were influenced by the removal of single variant; D, Funnel plot assessing heterogeneity. Blue line represents the inverse-variance weighted estimate, and dark blue line represents the MR-Egger estimate.

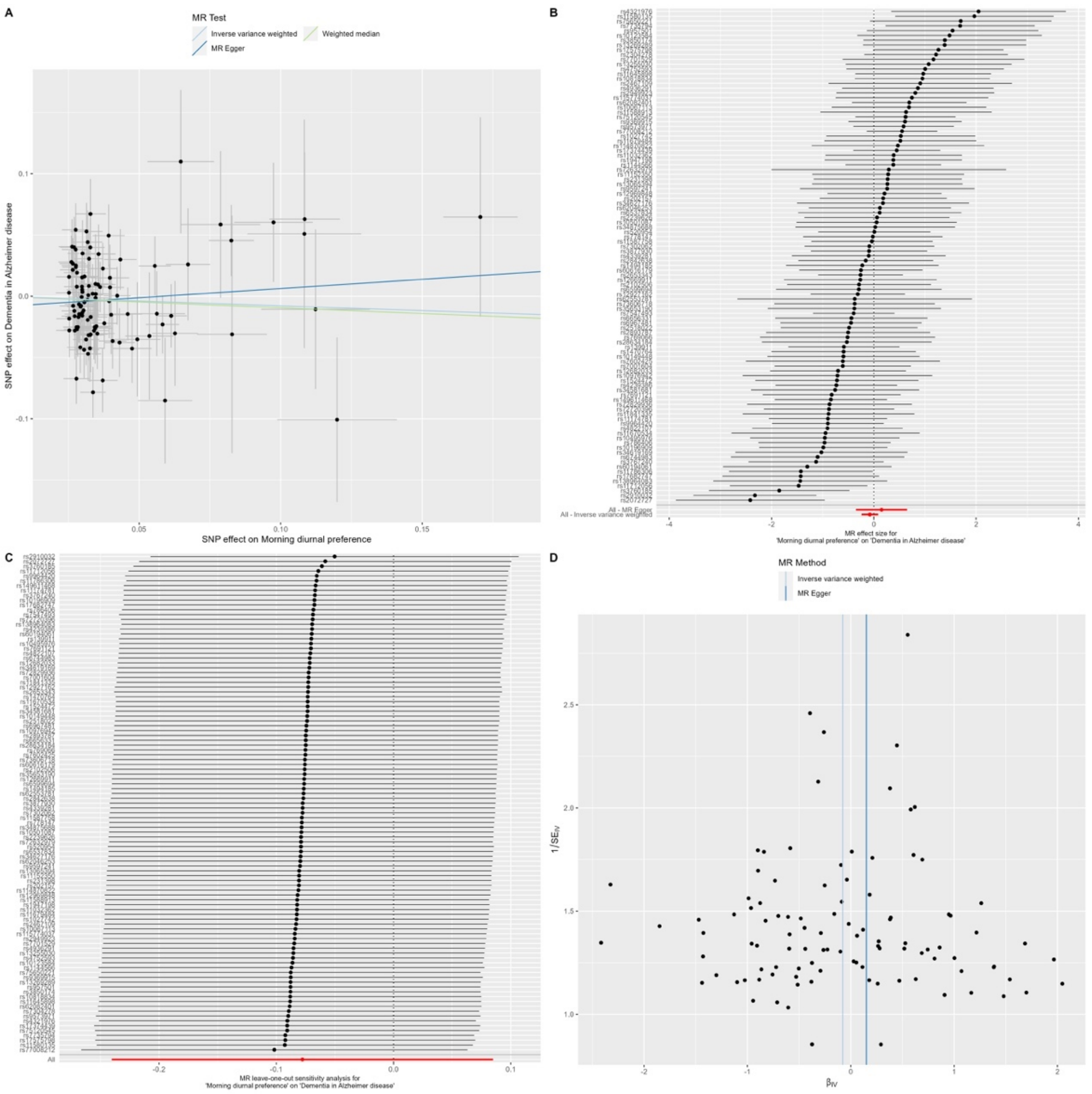

Supplementary Figure48. Mendelian randomization plots for the relationship of morning diurnal preference with dementia in Alzheimer disease

Note: A, Scatterplot of SNP effects on dementia in Alzheimer disease with the slope of each line corresponding to estimated MR effect (IVW, WM, and MR-E methods); B, Forest plot of individual and combined SNP MR-estimated effects sizes for relative dementia in Alzheimer disease; C, The leave-one-out plot visualized how the causal estimates (point with horizontal line) for the effect of morning diurnal preference on dementia in Alzheimer disease were influenced by the removal of single variant; D, Funnel plot assessing heterogeneity. Blue line represents the inverse-variance weighted estimate, and dark blue line represents the MR-Egger estimate.

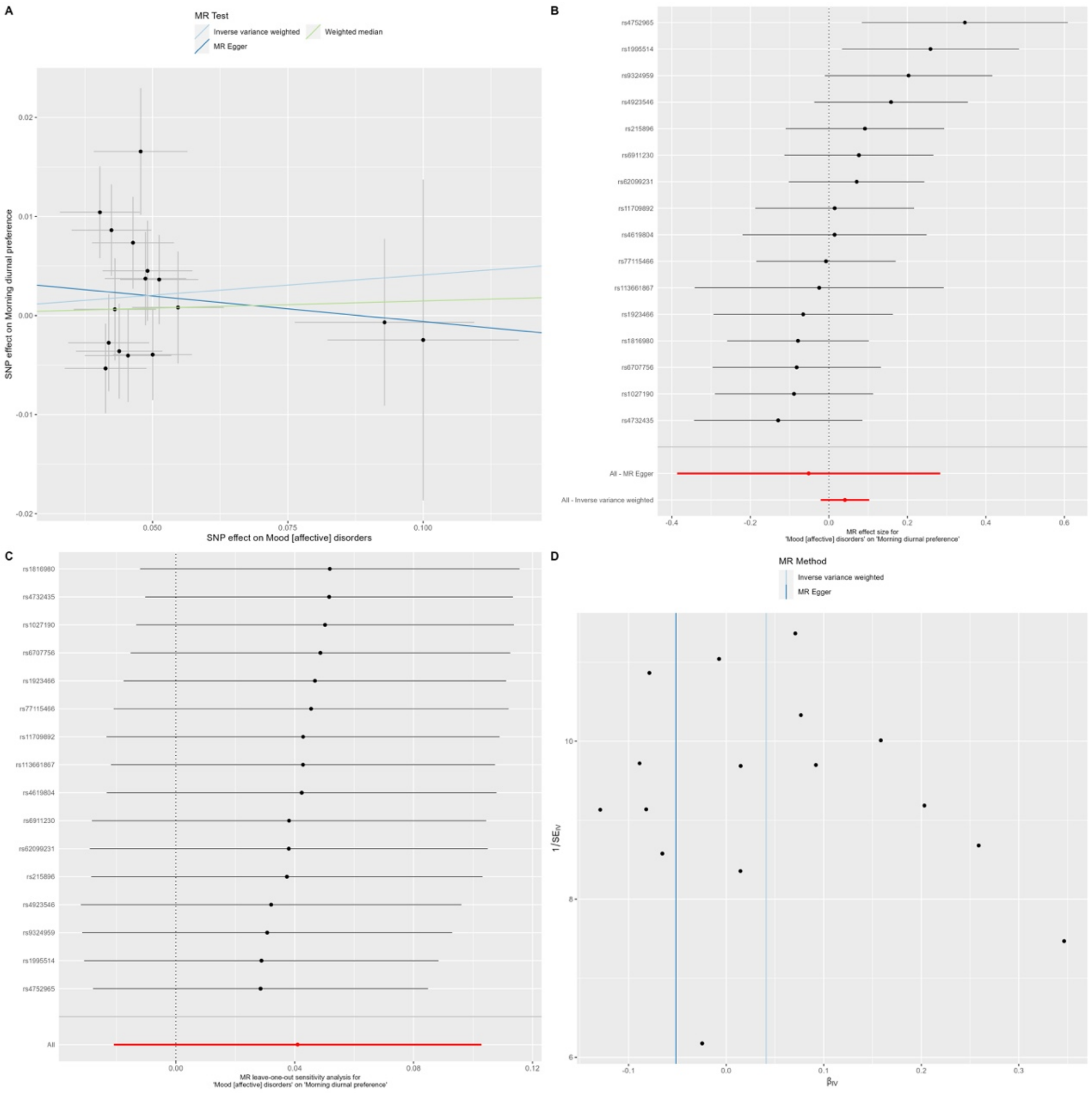

Supplementary Figure49. Reversed mendelian randomization plots for the relationship of mood [affective] disorders with morning diurnal preference

Note: A, Scatterplot of SNP effects on morning diurnal preference with the slope of each line corresponding to estimated MR effect (IVW, WM, and MR-E methods); B, Forest plot of individual and combined SNP MR-estimated effects sizes for relative morning diurnal preference; C, The leave-one-out plot visualized how the causal estimates (point with horizontal line) for the effect of mood [affective] disorders on morning diurnal preference were influenced by the removal of single variant; D, Funnel plot assessing heterogeneity. Blue line represents the inverse-variance weighted estimate, and dark blue line represents the MR-Egger estimate.

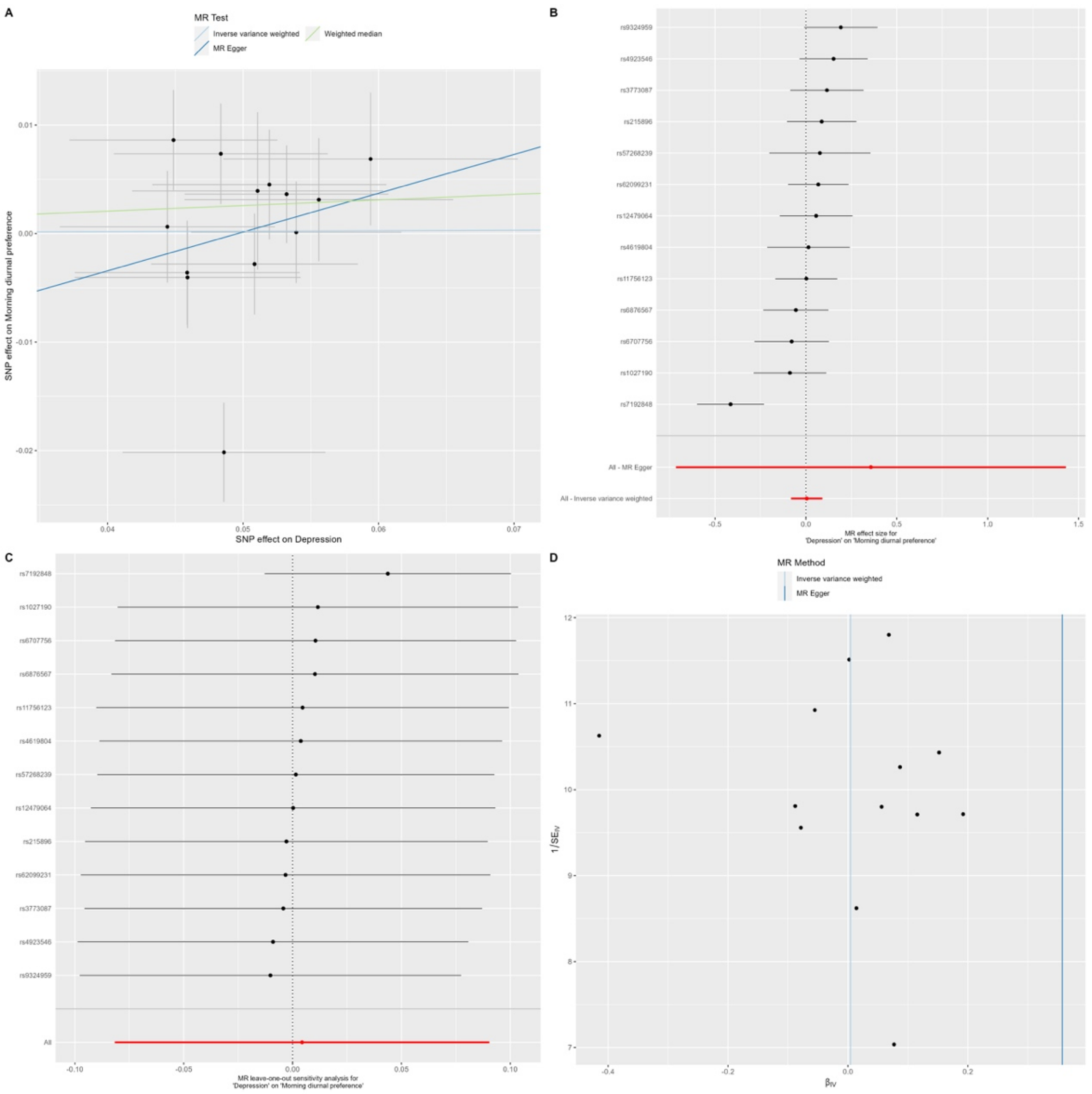

Supplementary Figure50. Reversed mendelian randomization plots for the relationship of depression with morning diurnal preference

Note: A, Scatterplot of SNP effects on morning diurnal preference with the slope of each line corresponding to estimated MR effect (IVW, WM, and MR-E methods); B, Forest plot of individual and combined SNP MR-estimated effects sizes for relative morning diurnal preference; C, The leave-one-out plot visualized how the causal estimates (point with horizontal line) for the effect of depression on morning diurnal preference were influenced by the removal of single variant; D, Funnel plot assessing heterogeneity. Blue line represents the inverse-variance weighted estimate, and dark blue line represents the MR-Egger estimate.

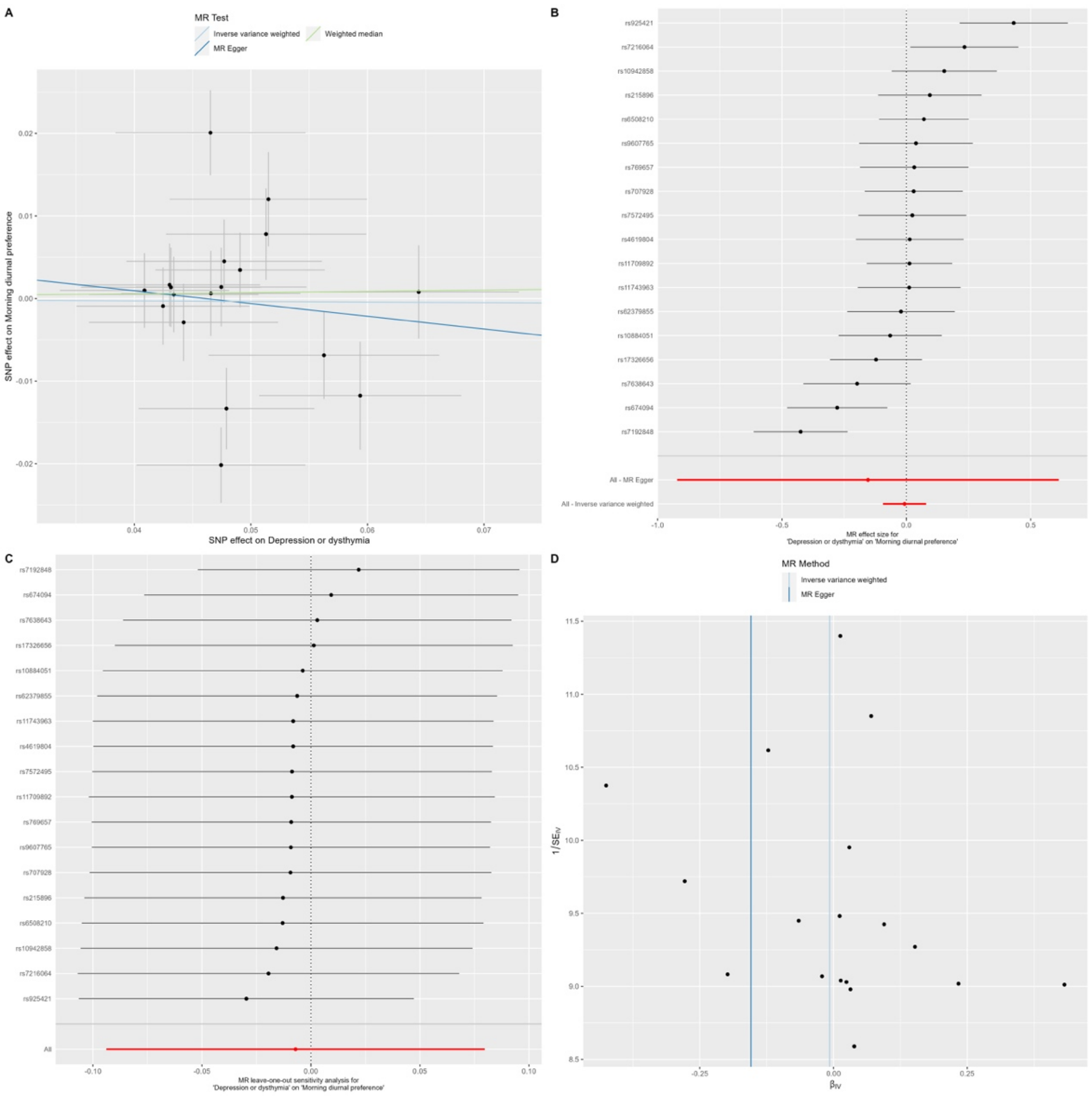

Supplementary Figure51. Reversed mendelian randomization plots for the relationship of depression or dysthymia with morning diurnal preference

Note: A, Scatterplot of SNP effects on morning diurnal preference with the slope of each line corresponding to estimated MR effect (IVW, WM, and MR-E methods); B, Forest plot of individual and combined SNP MR-estimated effects sizes for relative morning diurnal preference; C, The leave-one-out plot visualized how the causal estimates (point with horizontal line) for the effect of depression or dysthymia on morning diurnal preference were influenced by the removal of single variant; D, Funnel plot assessing heterogeneity. Blue line represents the inverse-variance weighted estimate, and dark blue line represents the MR-Egger estimate.

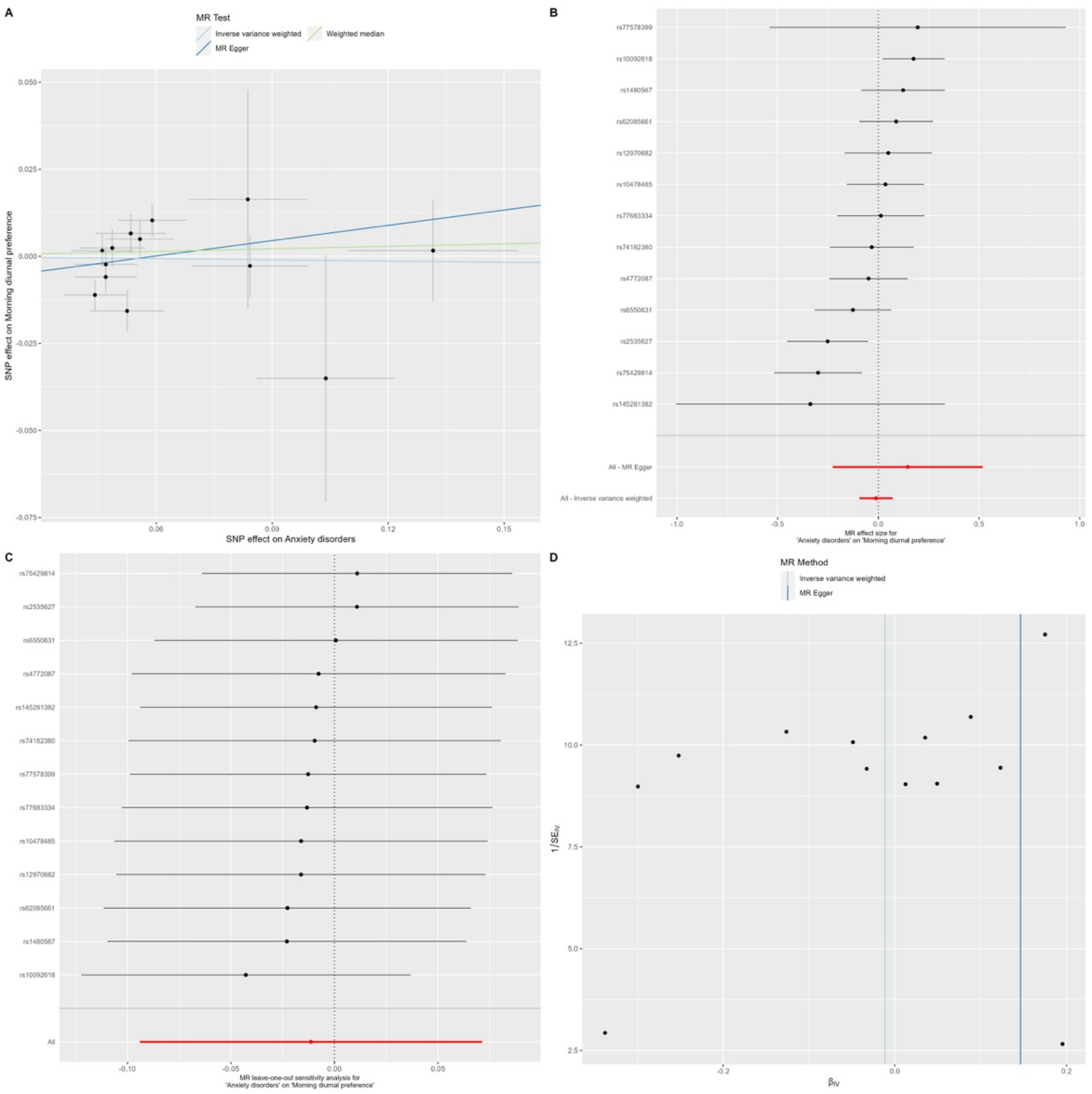

Supplementary Figure52. Reversed mendelian randomization plots for the relationship of anxiety disorders with morning diurnal preference

Note: A, Scatterplot of SNP effects on morning diurnal preference with the slope of each line corresponding to estimated MR effect (IVW, WM, and MR-E methods); B, Forest plot of individual and combined SNP MR-estimated effects sizes for relative morning diurnal preference; C, The leave-one-out plot visualized how the causal estimates (point with horizontal line) for the effect of anxiety disorders on morning diurnal preference were influenced by the removal of single variant; D, Funnel plot assessing heterogeneity. Blue line represents the inverse-variance weighted estimate, and dark blue line represents the MR-Egger estimate.

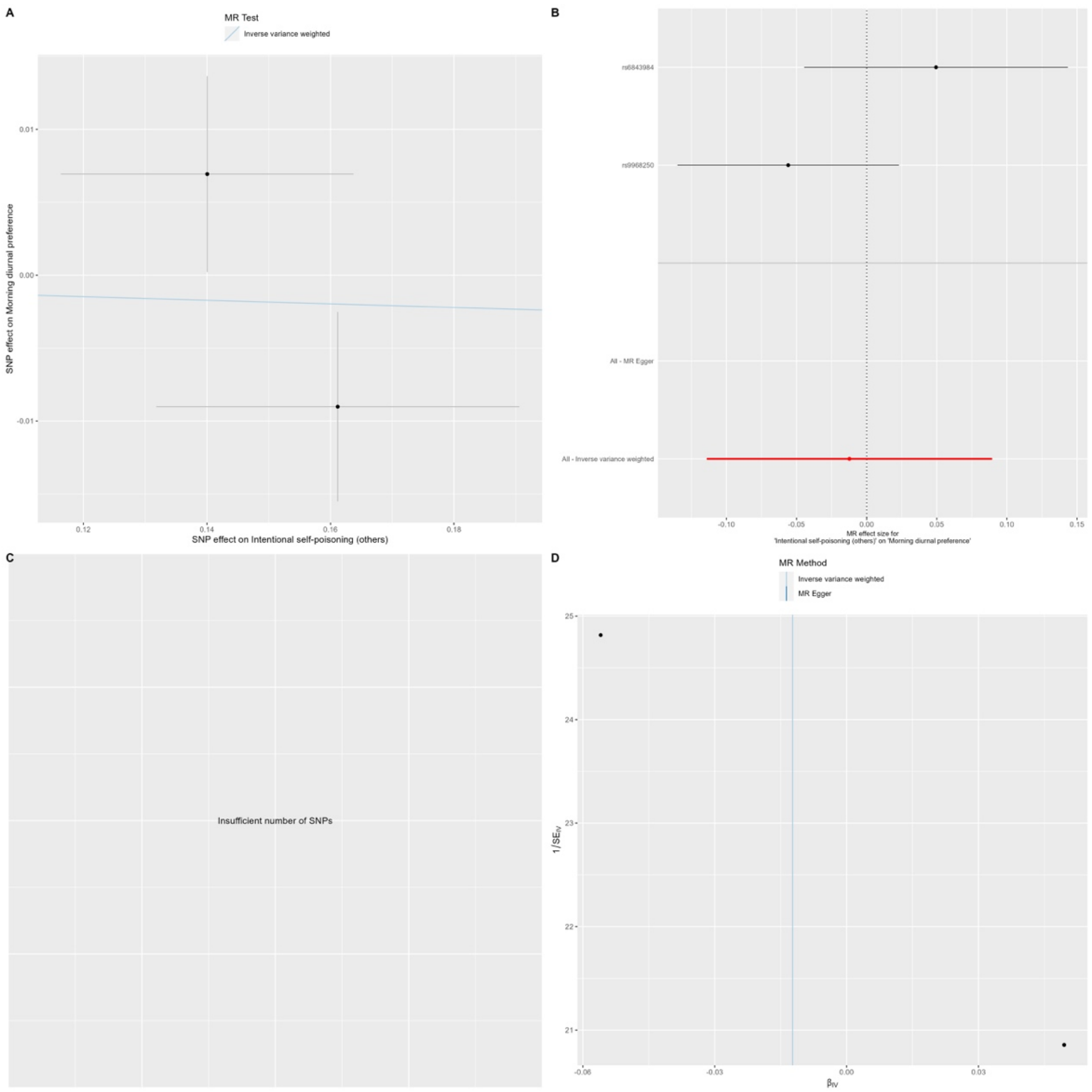

Supplementary Figure53. Reversed mendelian randomization plots for the relationship of intentional self-poisoning (others) with morning diurnal preference

Note: A, Scatterplot of SNP effects on morning diurnal preference with the slope of each line corresponding to estimated MR effect (IVW, WM, and MR-E methods); B, Forest plot of individual and combined SNP MR-estimated effects sizes for relative morning diurnal preference; C, The leave-one-out plot visualized how the causal estimates (point with horizontal line) for the effect of intentional self-poisoning (others) on morning diurnal preference were influenced by the removal of single variant; D, Funnel plot assessing heterogeneity. Blue line represents the inverse-variance weighted estimate, and dark blue line represents the MR-Egger estimate.

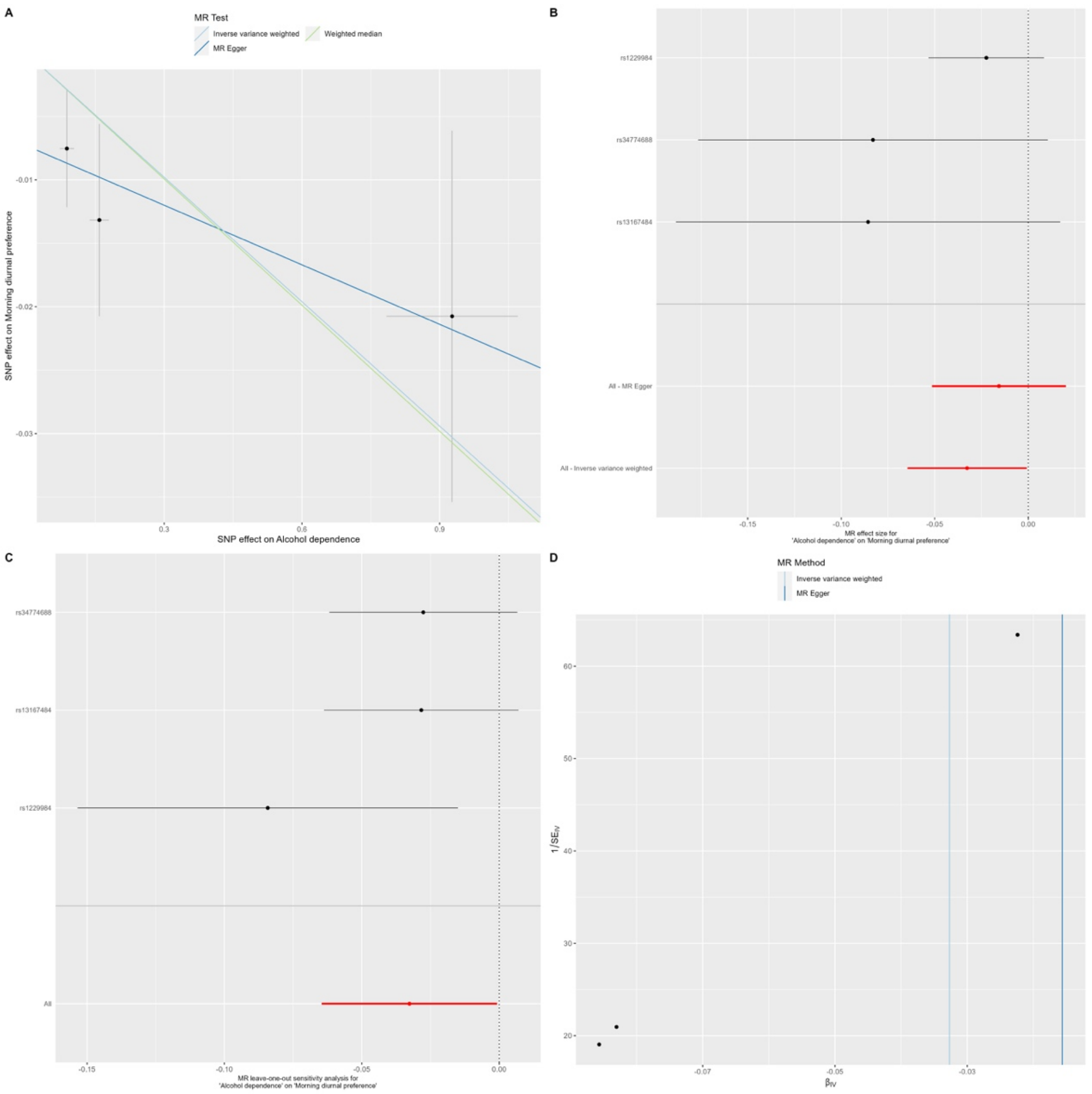

Supplementary Figure54. Reversed mendelian randomization plots for the relationship of alcohol dependence with morning diurnal preference

Note: A, Scatterplot of SNP effects on morning diurnal preference with the slope of each line corresponding to estimated MR effect (IVW, WM, and MR-E methods); B, Forest plot of individual and combined SNP MR-estimated effects sizes for relative morning diurnal preference; C, The leave-one-out plot visualized how the causal estimates (point with horizontal line) for the effect of alcohol dependence on morning diurnal preference were influenced by the removal of single variant; D, Funnel plot assessing heterogeneity. Blue line represents the inverse-variance weighted estimate, and dark blue line represents the MR-Egger estimate.

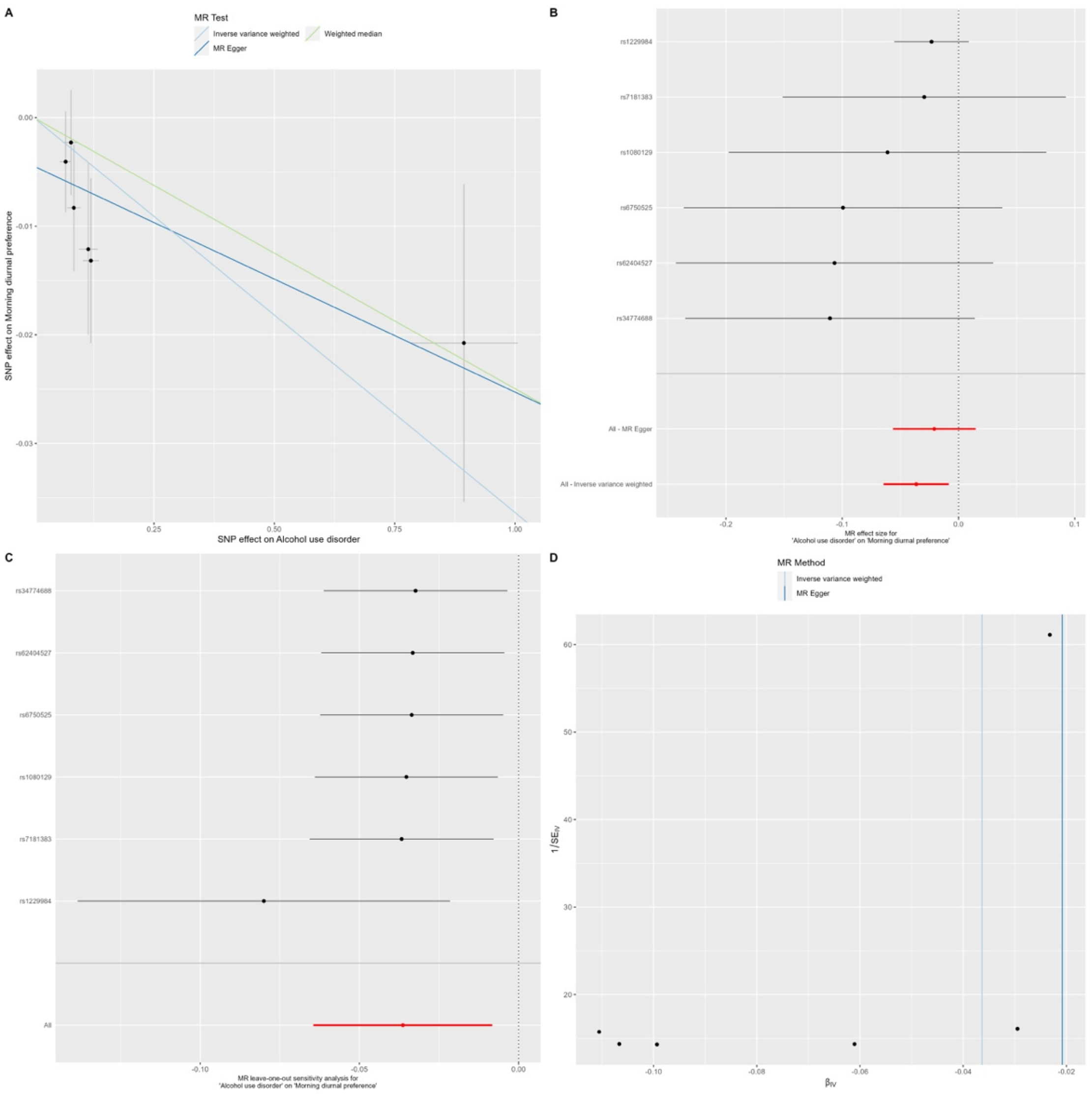

Supplementary Figure55. Reversed mendelian randomization plots for the relationship of alcohol use disorder with morning diurnal preference

Note: A, Scatterplot of SNP effects on morning diurnal preference with the slope of each line corresponding to estimated MR effect (IVW, WM, and MR-E methods); B, Forest plot of individual and combined SNP MR-estimated effects sizes for relative morning diurnal preference; C, The leave-one-out plot visualized how the causal estimates (point with horizontal line) for the effect of alcohol use disorder on morning diurnal preference were influenced by the removal of single variant; D, Funnel plot assessing heterogeneity. Blue line represents the inverse-variance weighted estimate, and dark blue line represents the MR-Egger estimate.

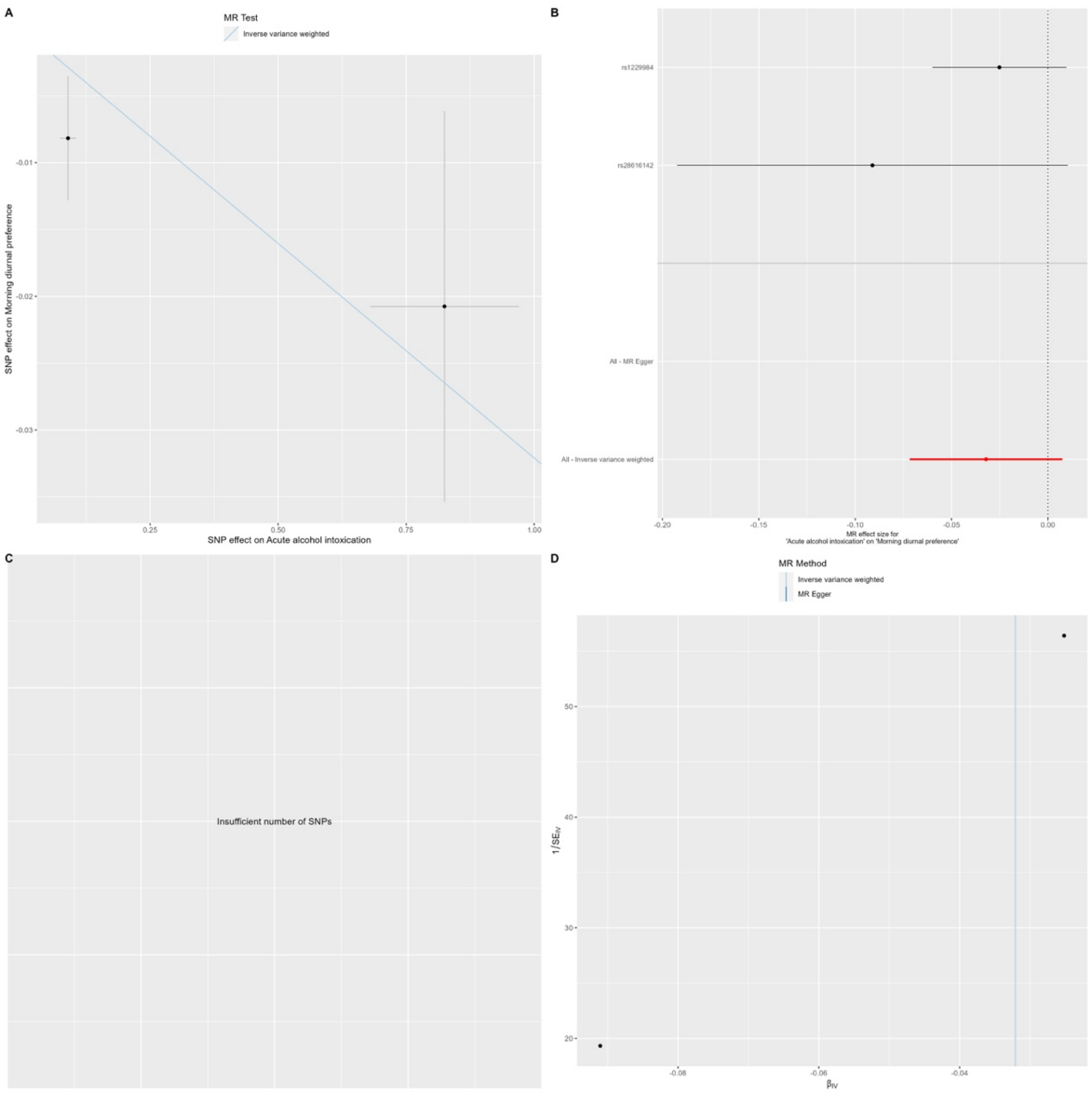

Supplementary Figure56. Reversed mendelian randomization plots for the relationship of acute alcohol intoxication with morning diurnal preference

Note: A, Scatterplot of SNP effects on morning diurnal preference with the slope of each line corresponding to estimated MR effect (IVW, WM, and MR-E methods); B, Forest plot of individual and combined SNP MR-estimated effects sizes for relative morning diurnal preference; C, The leave-one-out plot visualized how the causal estimates (point with horizontal line) for the effect of acute alcohol intoxication on morning diurnal preference were influenced by the removal of single variant; D, Funnel plot assessing heterogeneity. Blue line represents the inverse-variance weighted estimate, and dark blue line represents the MR-Egger estimate.

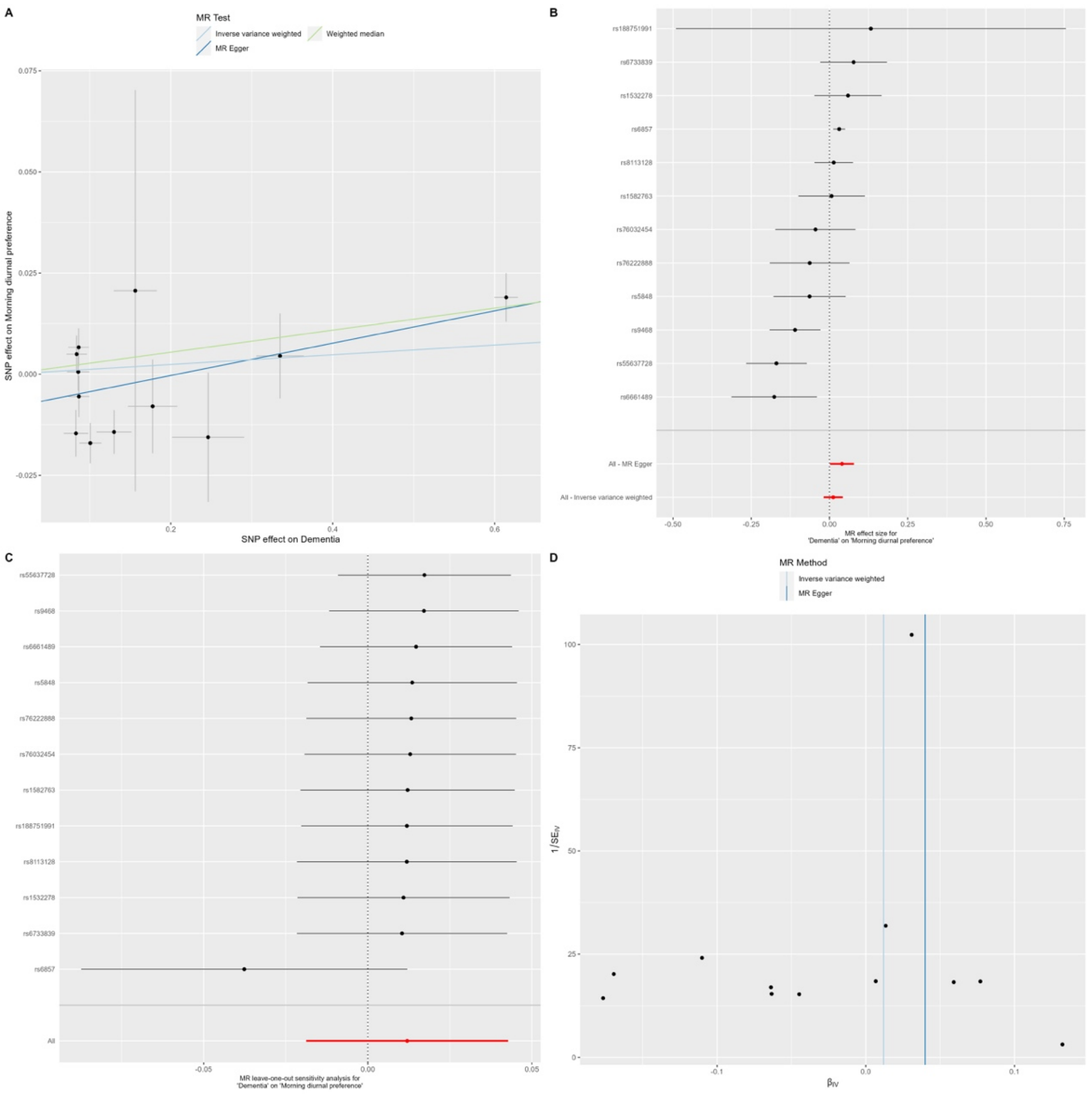

Supplementary Figure57. Reversed mendelian randomization plots for the relationship of dementia with morning diurnal preference

Note: A, Scatterplot of SNP effects on morning diurnal preference with the slope of each line corresponding to estimated MR effect (IVW, WM, and MR-E methods); B, Forest plot of individual and combined SNP MR-estimated effects sizes for relative morning diurnal preference; C, The leave-one-out plot visualized how the causal estimates (point with horizontal line) for the effect of dementia on morning diurnal preference were influenced by the removal of single variant; D, Funnel plot assessing heterogeneity. Blue line represents the inverse-variance weighted estimate, and dark blue line represents the MR-Egger estimate.

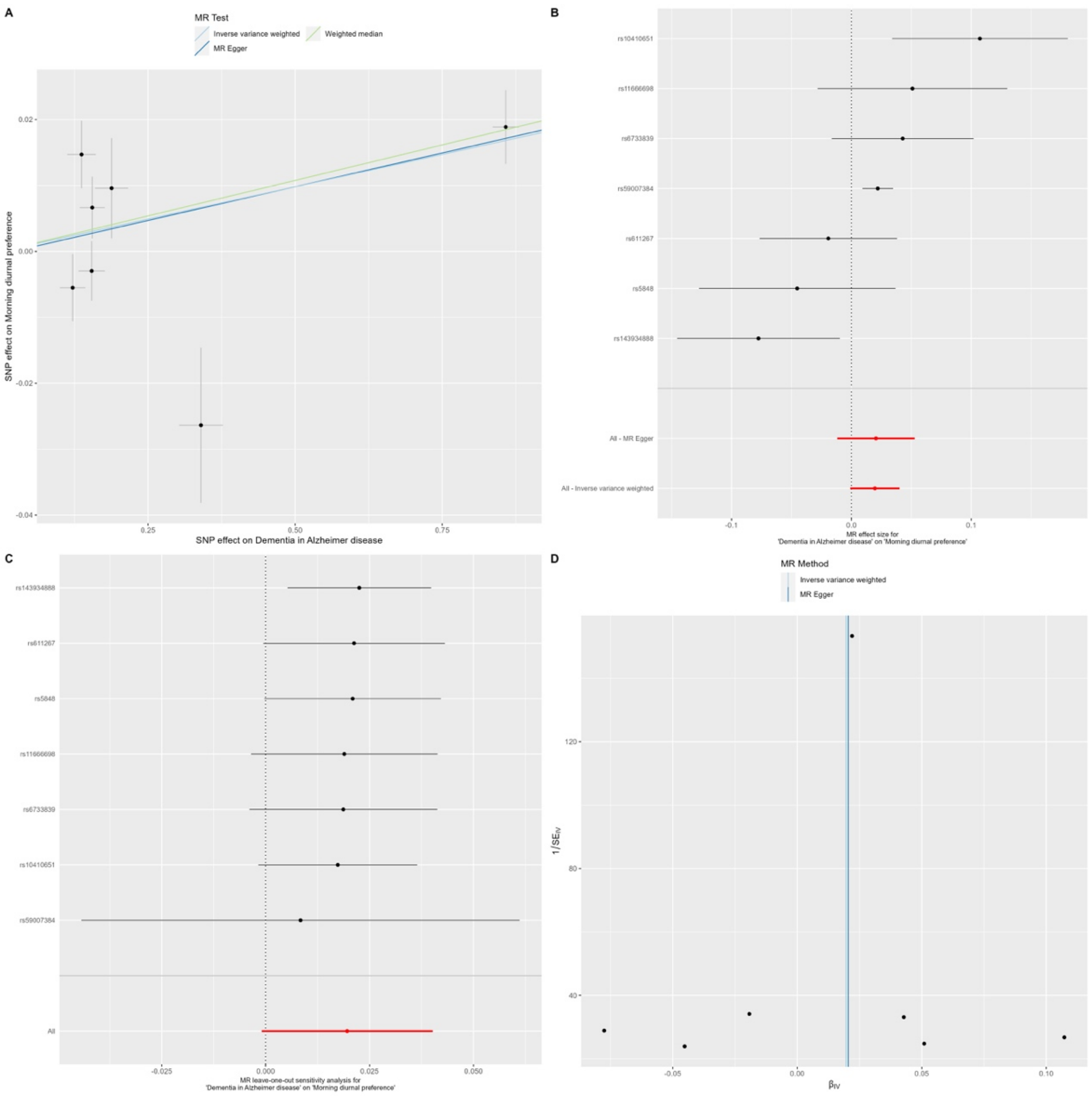

Supplementary Figure58. Reversed mendelian randomization plots for the relationship of dementia in Alzheimer disease with morning diurnal preference

Note: A, Scatterplot of SNP effects on morning diurnal preference with the slope of each line corresponding to estimated MR effect (IVW, WM, and MR-E methods); B, Forest plot of individual and combined SNP MR-estimated effects sizes for relative morning diurnal preference; C, The leave-one-out plot visualized how the causal estimates (point with horizontal line) for the effect of dementia in Alzheimer disease on morning diurnal preference were influenced by the removal of single variant; D, Funnel plot assessing heterogeneity. Blue line represents the inverse-variance weighted estimate, and dark blue line represents the MR-Egger estimate.
