# Supplementary material for: Exploring the Causal Association between Morning Diurnal Preference and Psychiatric Disorders: A Bidirectional Two-Sample Mendelian Randomization Analysis
Source: Life (Basel). 2024 Sep 25;14(10):1225. doi: 10.3390/life14101225 (PMC11508865; doi:10.3390/life14101225)
Supplement: Supplementary file 1 [file life-14-01225-s001.zip › Supplementary tables.pdf]

Supplementary Table1 Summary information on diurnal preference SNPs used as genetic instruments for the Mendelian randomization analyses

SNP	CHR	Effect allele	Other allele	Effect allele frequency	β	SE	*P*-value	Sample size	R^2	*F*-value
rs17374439	1	C	T	0.804	-0.056	0.006	1.20E-22	403195	0.001	393.220
rs3767240	1	T	C	0.618	-0.031	0.005	7.30E-12	403195	0.000	186.067
rs12140153	1	G	T	0.905	0.048	0.008	4.80E-10	403195	0.000	163.104
rs7547493	1	A	G	0.822	-0.058	0.006	2.50E-23	403195	0.001	401.240
rs11588913	1	G	A	0.601	0.025	0.005	2.50E-08	403195	0.000	123.152
rs72720396	1	A	G	0.769	-0.043	0.005	5.30E-16	403195	0.001	265.759
rs6656331	1	C	T	0.476	0.027	0.005	1.50E-09	403195	0.000	148.032
rs17575798	1	G	A	0.807	0.039	0.006	7.80E-12	403195	0.000	193.521
rs6537834	1	T	C	0.385	-0.025	0.005	3.50E-08	403195	0.000	121.465
rs11587758	1	G	A	0.604	-0.035	0.005	3.80E-14	403195	0.001	231.087
rs75650221	1	C	T	0.962	-0.065	0.012	3.30E-08	403195	0.000	124.475
rs11580135	1	T	C	0.317	-0.028	0.005	1.30E-08	403195	0.000	132.987
rs1144566	1	T	C	0.030	0.171	0.013	2.80E-38	403195	0.002	688.176
rs10196909	2	C	A	0.482	0.032	0.005	6.20E-13	403195	0.001	207.737
rs75120545	2	C	T	0.970	-0.097	0.014	2.90E-12	403195	0.001	224.340
rs786406	2	A	G	0.298	-0.033	0.005	1.00E-11	403195	0.000	180.752
rs10495976	2	A	T	0.610	-0.028	0.005	8.00E-10	403195	0.000	151.197
rs7602425	2	C	T	0.924	-0.054	0.009	3.20E-10	403195	0.000	161.913
rs778147	2	C	A	0.367	0.030	0.005	4.60E-10	403195	0.000	165.095
rs6744983	2	G	T	0.623	-0.025	0.005	4.90E-08	403195	0.000	121.557
rs10520176	2	T	C	0.502	0.033	0.005	2.00E-13	403195	0.001	224.561
rs28380327	2	A	T	0.629	0.029	0.005	7.80E-10	403195	0.000	158.134
rs1947198	2	C	T	0.878	-0.040	0.007	3.20E-09	403195	0.000	136.151
rs11679484	2	C	A	0.626	-0.030	0.005	1.60E-10	403195	0.000	168.243
rs138964083	2	C	T	0.941	-0.059	0.010	1.00E-09	403195	0.000	155.690
rs77008212	2	A	G	0.913	0.083	0.008	1.10E-24	403195	0.001	437.694
rs114870822	2	G	A	0.987	-0.108	0.020	3.40E-08	403195	0.000	119.663
rs34581681	3	G	A	0.839	0.034	0.006	4.20E-08	403195	0.000	129.379
rs149611468	3	T	C	0.988	0.120	0.021	1.30E-08	403195	0.000	137.343
rs13059636	3	A	G	0.528	-0.032	0.005	3.50E-12	403195	0.001	203.149
rs115774037	3	T	C	0.974	-0.079	0.014	3.50E-08	403195	0.000	127.295
rs11712056	3	T	C	0.556	0.032	0.005	3.70E-12	403195	0.001	203.344
rs60194061	3	G	A	0.730	-0.033	0.005	8.60E-11	403195	0.000	169.244
rs957501	3	T	A	0.336	0.026	0.005	3.00E-08	403195	0.000	124.643
rs13065394	3	G	T	0.712	0.030	0.005	2.20E-09	403195	0.000	146.951
rs2102506	3	G	A	0.361	0.029	0.005	8.30E-10	403195	0.000	159.988
rs3850174	3	T	A	0.743	0.032	0.005	1.40E-09	403195	0.000	156.311
rs2239626	3	T	C	0.695	-0.034	0.005	5.00E-12	403195	0.000	192.704
rs34627176	4	G	A	0.786	-0.030	0.006	3.60E-08	403195	0.000	121.090
rs231398	4	G	A	0.838	0.035	0.006	5.80E-09	403195	0.000	137.727
rs12498561	4	C	G	0.495	-0.025	0.005	4.60E-08	403195	0.000	122.872
rs28634184	4	C	T	0.749	0.030	0.005	1.30E-08	403195	0.000	135.498
rs7691121	4	C	G	0.768	0.034	0.005	1.00E-10	403195	0.000	166.699
rs4339281	4	A	G	0.873	0.039	0.007	5.30E-09	403195	0.000	138.890
rs4241964	4	T	G	0.525	-0.033	0.005	7.20E-13	403195	0.001	214.359
rs10067113	5	C	T	0.381	-0.028	0.005	2.50E-09	403195	0.000	149.732
rs7701529	5	A	T	0.238	-0.030	0.005	1.70E-08	403195	0.000	131.615
rs34875688	5	T	A	0.767	0.032	0.005	1.10E-09	403195	0.000	147.780
rs12055234	5	G	A	0.672	-0.027	0.005	1.50E-08	403195	0.000	131.121
rs1027742	5	A	G	0.738	0.031	0.005	3.60E-09	403195	0.000	146.870
rs2910032	5	C	T	0.482	-0.034	0.005	1.10E-13	403195	0.001	228.744
rs7735794	5	G	A	0.776	-0.031	0.006	4.50E-08	403195	0.000	138.194
rs9369915	6	G	A	0.694	-0.037	0.005	3.00E-14	403195	0.001	236.920
rs2842638	6	T	G	0.577	0.031	0.005	1.90E-11	403195	0.000	185.071
rs2653343	6	T	A	0.215	0.061	0.005	5.20E-29	403195	0.001	512.305
rs60616179	6	A	G	0.945	0.056	0.010	9.20E-09	403195	0.000	132.524
rs520954	6	A	G	0.673	-0.042	0.005	1.60E-18	403195	0.001	317.238
rs3877930	6	A	G	0.986	-0.112	0.019	3.00E-09	403195	0.000	139.883
rs9365769	6	A	G	0.437	0.026	0.005	7.30E-09	403195	0.000	137.089
rs1494185	7	G	A	0.726	0.029	0.005	1.10E-08	403195	0.000	133.069
rs12669911	7	A	C	0.384	-0.029	0.005	1.80E-10	403195	0.000	165.781
rs6967481	7	C	T	0.503	-0.030	0.005	1.90E-11	403195	0.000	180.580
rs202157	7	C	T	0.299	0.040	0.005	1.30E-15	403195	0.001	266.649
rs4729854	7	T	A	0.517	0.044	0.005	1.90E-21	403195	0.001	395.697
rs1524472	7	A	G	0.441	0.025	0.005	2.10E-08	403195	0.000	127.232

rs13269289	8	G	A	0.673	-0.028	0.005	5.50E-09	403195	0.000	134.932
rs11786306	8	G	C	0.646	-0.029	0.005	7.00E-10	403195	0.000	157.469
rs13255030	8	A	G	0.585	0.026	0.005	1.50E-08	403195	0.000	133.175
rs7001604	8	T	C	0.613	-0.032	0.005	7.30E-12	403195	0.000	193.989
rs769066	8	T	C	0.816	-0.033	0.006	1.90E-08	403195	0.000	131.047
rs4321976	8	T	C	0.779	0.033	0.005	8.70E-10	403195	0.000	149.791
rs1470764	8	G	A	0.387	0.029	0.005	5.90E-10	403195	0.000	156.358
rs12682033	8	T	C	0.240	0.035	0.005	6.10E-11	403195	0.000	179.242
rs10976942	9	C	A	0.917	-0.049	0.008	1.00E-09	403195	0.000	149.409
rs308521	9	T	C	0.603	0.028	0.005	1.50E-09	403195	0.000	150.432
rs62553781	9	C	T	0.965	0.083	0.012	3.00E-11	403195	0.000	186.557
rs10123584	9	G	A	0.293	0.027	0.005	4.00E-08	403195	0.000	122.128
rs10818834	9	T	C	0.733	0.032	0.005	4.20E-10	403195	0.000	164.695
rs34619169	9	G	A	0.692	-0.027	0.005	1.50E-08	403195	0.000	127.928
rs28458909	9	C	T	0.876	0.068	0.007	4.70E-23	403195	0.001	399.983
rs2893787	10	G	A	0.255	0.029	0.005	3.50E-08	403195	0.000	127.604
rs4752593	10	G	C	0.372	0.027	0.005	9.80E-09	403195	0.000	132.951
rs6599694	10	G	T	0.656	0.027	0.005	2.10E-08	403195	0.000	129.454
rs72632979	11	A	G	0.829	0.035	0.006	4.00E-09	403195	0.000	137.150
rs10501087	11	T	C	0.799	-0.035	0.006	5.40E-10	403195	0.000	155.675
rs512647	11	G	C	0.556	-0.024	0.005	4.20E-08	403195	0.000	119.395
rs11032362	11	G	A	0.909	-0.067	0.008	7.30E-18	403195	0.001	301.640
rs11229543	11	G	A	0.760	0.034	0.005	1.10E-10	403195	0.000	167.407
rs4936291	11	A	G	0.611	-0.028	0.005	9.40E-09	403195	0.000	146.568
rs73606718	11	G	A	0.879	0.040	0.007	5.70E-09	403195	0.000	138.028
rs2467109	12	T	A	0.718	-0.028	0.005	3.90E-08	403195	0.000	125.111
rs7302062	12	T	C	0.553	0.032	0.005	5.20E-12	403195	0.000	201.380
rs7313852	12	G	A	0.565	0.048	0.005	3.80E-25	403195	0.001	451.644
rs11174781	12	T	C	0.878	0.048	0.007	3.90E-12	403195	0.000	195.662
rs7959983	12	T	C	0.596	-0.032	0.005	4.70E-12	403195	0.000	196.817
rs7304278	12	A	G	0.275	-0.033	0.005	9.60E-11	403195	0.000	172.775
rs9597241	13	A	C	0.811	0.032	0.006	2.70E-08	403195	0.000	128.481
rs9573971	13	A	G	0.966	0.108	0.012	4.90E-18	403195	0.001	310.611
rs11841335	13	G	A	0.746	0.028	0.005	4.00E-08	403195	0.000	123.060
rs10149448	14	A	G	0.604	0.027	0.005	5.60E-09	403195	0.000	143.124
rs12927162	16	A	G	0.722	0.046	0.005	2.00E-20	403195	0.001	342.775
rs1421085	16	T	C	0.597	-0.042	0.005	1.80E-19	403195	0.001	336.501
rs62046253	16	C	T	0.656	-0.030	0.005	1.40E-10	403195	0.000	163.522
rs11645898	16	T	C	0.833	0.036	0.006	1.80E-09	403195	0.000	142.850
rs2518022	17	T	C	0.084	0.063	0.008	7.60E-15	403195	0.001	242.582
rs3760185	17	C	T	0.752	0.037	0.005	2.10E-12	403195	0.001	207.575
rs72829936	17	G	A	0.836	-0.035	0.006	5.50E-09	403195	0.000	137.232
rs35653190	17	C	T	0.772	0.030	0.005	2.00E-08	403195	0.000	130.927
rs17682747	17	G	A	0.767	-0.031	0.005	8.70E-09	403195	0.000	134.308
rs2949923	17	A	G	0.539	-0.027	0.005	4.20E-09	403195	0.000	141.348
rs62082401	18	C	G	0.809	-0.043	0.006	1.20E-14	403195	0.001	234.123
rs4239386	18	T	A	0.665	0.034	0.005	4.20E-13	403195	0.001	211.652
rs12969848	18	C	T	0.470	-0.036	0.005	2.60E-15	403195	0.001	263.425
rs9962650	18	C	G	0.577	-0.026	0.005	5.80E-09	403195	0.000	137.138
rs9964420	18	C	A	0.696	0.041	0.005	2.40E-16	403195	0.001	281.767
rs11152350	18	A	C	0.471	-0.028	0.005	6.40E-10	403195	0.000	157.433
rs9636202	19	G	A	0.734	0.028	0.005	2.70E-08	403195	0.000	124.495
rs11670534	19	C	T	0.834	0.033	0.006	4.70E-08	403195	0.000	120.445
rs78095690	20	T	C	0.564	-0.028	0.005	1.10E-09	403195	0.000	151.007
rs2072727	20	T	C	0.436	0.028	0.005	5.60E-10	403195	0.000	153.721
rs139911	22	C	T	0.424	0.038	0.005	2.20E-16	403195	0.001	280.217
rs4822107	22	G	A	0.494	-0.028	0.005	8.70E-10	403195	0.000	156.330

Note: This study first identified SNPs with p-value <5×10-8, and further performed PLINK clumping with a cut-off value of R2 =0.001 and a gene distance window =10000 kb based on the European populations in the 1000 genomes reference panel. The SNP with the lowest p-value was retained when in linkage disequilibrium.

Supplementary Table2 Summary information on psychiatric disorders SNPs used as genetic instruments for the Mendelian randomization analyses

SNP	CHR	Effect allele	Other allele	Effect allele frequency	β	SE	*P*-value	Sample size	R2	F-value
Mood [affective] disorders										
rs77115466	2	A	T	0.047	0.093	0.017	2.16E-08	377277	7.77E-04	293.303
rs6707756	2	C	T	0.290	-0.044	0.008	3.64E-08	377277	7.92E-04	298.956
rs4619804	3	C	A	0.662	0.043	0.008	1.57E-08	377277	8.30E-04	313.226
rs11709892	3	C	T	0.772	-0.055	0.008	1.08E-10	377277	1.06E-03	398.542
rs113661867	4	T	C	0.041	0.100	0.018	1.49E-08	377277	7.80E-04	294.499
rs1816980	5	A	C	0.582	-0.050	0.007	6.12E-12	377277	1.22E-03	459.930
rs9324959	5	A	G	0.390	0.042	0.007	8.29E-09	377277	8.57E-04	323.418
rs1923466	6	C	A	0.367	-0.042	0.007	2.10E-08	377277	8.17E-04	308.306
rs6911230	6	G	A	0.655	-0.049	0.008	9.40E-11	377277	1.07E-03	405.109
rs4732435	7	G	A	0.634	0.041	0.008	4.22E-08	377277	7.93E-04	299.263
rs1027190	10	G	T	0.722	-0.045	0.008	1.32E-08	377277	8.30E-04	313.412
rs4923546	11	A	G	0.351	-0.046	0.008	8.87E-10	377277	9.80E-04	370.261
rs4752965	11	A	G	0.227	-0.048	0.009	3.22E-08	377277	8.03E-04	303.223
rs1995514	12	A	G	0.392	-0.040	0.007	4.76E-08	377277	7.73E-04	291.909
rs215896	16	C	T	0.248	0.049	0.008	3.22E-09	377277	8.99E-04	339.454
rs12449073	16	T	A	0.467	0.045	0.007	4.63E-10	377277	9.98E-04	376.916
rs62099231	18	A	G	0.460	0.051	0.007	1.22E-12	377277	1.30E-03	492.710
Depression										
rs12479064	2	T	C	0.169	0.056	0.010	2.03E-08	372472	8.68E-04	323.611
rs6707756	2	C	T	0.290	-0.046	0.008	3.31E-08	372472	8.66E-04	322.949
rs4619804	3	C	A	0.662	0.044	0.008	2.20E-08	372472	8.83E-04	329.238
rs3773087	3	C	T	0.142	-0.059	0.011	4.51E-08	372472	8.58E-04	319.948
rs530758485	3	A	G	0.107	0.081	0.012	6.88E-12	372472	1.27E-03	472.941
rs6876567	5	G	A	0.399	0.051	0.008	2.69E-11	372472	1.24E-03	462.552
rs9324959	5	A	G	0.390	0.045	0.008	5.02E-09	372472	9.58E-04	357.239
rs11756123	6	T	A	0.639	-0.054	0.008	3.61E-12	372472	1.34E-03	500.680
rs57268239	9	A	C	0.204	0.051	0.009	3.48E-08	372472	8.47E-04	315.728
rs1027190	10	G	T	0.722	-0.046	0.008	3.73E-08	372472	8.45E-04	315.061
rs4923546	11	A	G	0.351	-0.048	0.008	9.02E-10	372472	1.06E-03	397.046
rs215896	16	C	T	0.248	0.052	0.009	1.83E-09	372472	1.01E-03	375.214
rs7192848	16	G	A	0.469	0.049	0.007	8.58E-11	372472	1.18E-03	438.513
rs57852066	16	A	G	0.049	0.094	0.017	2.20E-08	372472	8.31E-04	309.694
rs62099231	18	A	G	0.459	0.053	0.008	1.48E-12	372472	1.41E-03	524.598
Depression or dysthymia										
rs7572495	2	A	G	0.520	-0.041	0.007	1.78E-08	274330	8.34E-04	228.964
rs17326656	2	T	G	0.164	-0.056	0.010	1.30E-08	274330	8.68E-04	238.364
rs4619804	3	C	A	0.661	0.047	0.008	1.36E-09	274330	9.72E-04	266.924
rs11709892	3	C	T	0.773	-0.064	0.009	6.11E-14	274330	1.46E-03	400.215
rs7638643	3	T	C	0.229	-0.059	0.009	7.30E-12	274330	1.24E-03	341.591
rs925421	4	G	A	0.731	-0.047	0.008	1.19E-08	274330	8.51E-04	233.645
rs769657	4	G	A	0.335	-0.043	0.008	2.29E-08	274330	8.29E-04	227.610
rs10942858	5	C	A	0.766	0.051	0.009	2.37E-09	274330	9.44E-04	259.240
rs147831713	5	C	A	0.348	0.055	0.008	5.23E-13	274330	1.36E-03	373.613
rs62379855	5	G	T	0.394	0.042	0.007	1.04E-08	274330	8.61E-04	236.454
rs11743963	5	T	C	0.479	-0.043	0.007	2.47E-09	274330	9.40E-04	257.975
rs707928	6	G	A	0.433	-0.047	0.007	8.91E-11	274330	1.11E-03	303.654
rs10884051	10	G	A	0.723	-0.044	0.008	4.90E-08	274330	7.83E-04	214.992
rs674094	11	C	A	0.644	-0.048	0.008	2.13E-10	274330	1.05E-03	289.090
rs215896	16	C	T	0.247	0.048	0.008	1.29E-08	274330	8.47E-04	232.601
rs7192848	16	G	A	0.469	0.047	0.007	5.77E-11	274330	1.12E-03	307.807
rs57852066	16	A	G	0.049	0.104	0.016	1.89E-10	274330	1.02E-03	279.676
rs7216064	17	A	G	0.238	0.052	0.008	1.16E-09	274330	9.62E-04	264.021
rs6508210	18	C	A	0.487	0.049	0.007	1.45E-11	274330	1.20E-03	330.425
rs9607765	22	T	C	0.319	0.043	0.008	3.02E-08	274330	8.05E-04	220.916
Anxiety disorders										
rs75429814	2	C	T	0.232	-0.052	0.010	4.58E-08	317717	9.82E-04	312.176
rs74182380	2	A	G	0.080	-0.084	0.015	2.40E-08	317717	1.04E-03	332.115
rs6550631	3	C	T	0.539	0.047	0.008	6.03E-09	317717	1.09E-03	347.919
rs2535627	3	C	T	0.477	-0.044	0.008	4.62E-08	317717	9.71E-04	308.885
rs10478485	5	T	C	0.484	0.046	0.008	1.23E-08	317717	1.06E-03	336.209
rs77578399	6	C	T	0.077	-0.084	0.015	4.06E-08	317717	1.00E-03	317.993
rs10092618	8	A	G	0.708	0.059	0.009	4.46E-11	317717	1.44E-03	456.751
rs77683334	12	T	C	0.037	-0.132	0.022	2.04E-09	317717	1.22E-03	388.916
rs4772087	13	T	C	0.415	0.047	0.008	9.22E-09	317717	1.07E-03	340.155

rs1480567	16	C	T	0.262	0.053	0.009	3.78E-09	317717	1.10E-03	350.666
rs145281382	17	T	C	0.052	0.104	0.018	5.43E-09	317717	1.05E-03	335.493
rs62085661	17	G	C	0.721	-0.056	0.009	4.23E-10	317717	1.25E-03	397.472
rs12970682	18	A	G	0.336	-0.049	0.009	1.21E-08	317717	1.05E-03	335.031
Intentional self-poisoning (others)										
rs9968250	4	G	A	0.131	-0.161	0.029	4.16E-08	377277	5.91E-03	2244.344
rs6843984	4	G	C	0.217	-0.140	0.024	3.44E-09	377277	6.67E-03	2534.971
Alcohol dependence										
rs1229984	4	C	T	0.995	0.927	0.143	8.86E-11	363231	8.84E-03	3239.858
rs34774688	4	T	A	0.137	0.159	0.021	2.04E-14	363231	5.96E-03	2176.758
rs13167484	5	G	C	0.615	0.088	0.015	1.01E-08	363231	3.67E-03	1338.876
Alcohol use disorder										
rs6750525	2	C	G	0.222	0.084	0.014	2.37E-09	377277	2.42E-03	914.970
rs1229984	4	C	T	0.995	0.894	0.112	1.36E-15	377277	8.14E-03	3095.732
rs34774688	4	T	A	0.137	0.119	0.017	8.36E-13	377277	3.37E-03	1274.512
rs62404527	6	C	T	0.097	0.114	0.019	4.84E-09	377277	2.27E-03	857.528
rs1080129	14	C	T	0.410	-0.067	0.012	3.36E-08	377277	2.15E-03	811.577
rs7181383	15	T	C	0.770	-0.078	0.014	2.52E-08	377277	2.14E-03	810.668
Acute alcohol intoxication										
rs1229984	4	C	T	0.995	0.825	0.145	1.26E-08	362312	7.01E-03	2559.077
rs28616142	15	T	C	0.361	0.090	0.016	1.60E-08	362312	3.72E-03	1351.360
Dementia										
rs6661489	1	C	T	0.792	-0.083	0.015	4.10E-08	373159	2.26E-03	845.845
rs6733839	2	T	C	0.374	0.086	0.013	2.17E-11	373159	3.48E-03	1301.964
rs1532278	8	C	T	0.584	0.084	0.013	3.37E-11	373159	3.40E-03	1274.319
rs76222888	9	C	T	0.019	0.246	0.045	3.32E-08	373159	2.22E-03	828.421
rs1582763	11	A	G	0.282	-0.086	0.014	7.13E-10	373159	2.97E-03	1111.404
rs5848	17	T	C	0.344	0.086	0.013	3.19E-11	373159	3.37E-03	1262.592
rs9468	17	C	T	0.097	-0.130	0.022	1.84E-09	373159	2.95E-03	1102.611
rs6857	19	T	C	0.196	0.614	0.015	0	373159	1.19E-01	50256.065
rs143473297	19	C	T	0.040	0.703	0.029	1.73E-133	373159	3.75E-02	14551.480
rs8113128	19	C	A	0.043	0.335	0.029	5.57E-30	373159	9.18E-03	3457.297
rs55637728	19	T	C	0.309	-0.101	0.014	9.54E-14	373159	4.32E-03	1620.806
rs76032454	19	A	G	0.041	0.177	0.031	6.17E-09	373159	2.46E-03	919.753
rs188751991	20	T	C	0.055	0.156	0.027	4.68E-09	373159	2.55E-03	953.433
Dementia in Alzheimer disease										
rs6733839	2	T	C	0.373	0.155	0.021	2.36E-13	362082	1.13E-02	4132.361
rs611267	11	G	A	0.682	0.154	0.022	6.18E-12	362082	1.03E-02	3778.244
rs5848	17	T	C	0.343	0.122	0.022	1.63E-08	362082	6.70E-03	2442.386
rs10410651	19	C	T	0.243	-0.137	0.024	1.54E-08	362082	6.91E-03	2519.032
rs143934888	19	G	A	0.073	0.340	0.037	5.22E-20	362082	1.56E-02	5756.016
rs59007384	19	T	G	0.218	0.858	0.022	0	362082	2.51E-01	121551.893
rs7246703	19	G	C	0.551	-0.264	0.021	8.73E-38	362082	3.45E-02	12957.461
rs11666698	19	G	A	0.152	0.188	0.028	1.76E-11	362082	9.12E-03	3330.800

Supplementary Table3 GWAS summary statistics: Overview of the data sources of the instrumental variables used in the MR study

Variables	Case number	Control number	Ancestry	Download
**Diurnal preference**				
Morning person	252287	150908	European	https://doi.org/10.1038/s41467-018-08259-7
**Psychiatric disorders**				
**Mood disorders**				
Mood [affective] disorders	48085	329192	Europe. FIN	https://storage.googleapis.com/finngen-public-data-r9/summary_stats/finngen_R9_F5_MOOD.gz
Persistent mood disorders	6756	329192	Europe. FIN	https://storage.googleapis.com/finngen-public-data-r9/summary_stats/finngen_R9_F5_PERSMOOD.gz
Depression	43280	329192	Europe. FIN	https://storage.googleapis.com/finngen-public-data-r9/summary_stats/finngen_R9_F5_DEPRESSIO.gz
Depression or dysthymia	48847	225483	Europe. FIN	https://storage.googleapis.com/finngen-public-data-r9/summary_stats/finngen_R9_F5_DEPRESSION_DYSTHYMIA.gz
Bipolar affective disorders	7006	329192	Europe. FIN	https://storage.googleapis.com/finngen-public-data-r9/summary_stats/finngen_R9_F5_BIPO.gz
Manic episode	934	329192	Europe. FIN	https://storage.googleapis.com/finngen-public-data-r9/summary_stats/finngen_R9_F5_MANIA.gz
**Anxiety disorders**				
Phobic anxiety disorders	4303	337577	Europe. FIN	https://storage.googleapis.com/finngen-public-data-r9/summary_stats/finngen_R9_F5_PHOBANX.gz
Anxiety disorders	40191	277526	Europe. FIN	https://storage.googleapis.com/finngen-public-data-r9/summary_stats/finngen_R9_F5_PHOBANX.gz
Other anxiety disorders	16887	337577	Europe. FIN	https://storage.googleapis.com/finngen-public-data-r9/summary_stats/finngen_R9_F5_ANXIETY.gz
Anxious personality disorder	534	366637	Europe. FIN	https://storage.googleapis.com/finngen-public-data-r9/summary_stats/finngen_R9_F5_ANXPER.gz
Panic disorder	4801	337577	Europe. FIN	https://storage.googleapis.com/finngen-public-data-r9/summary_stats/finngen_R9_F5_PANIC.gz
Agoraphobia	890	337577	Europe. FIN	https://storage.googleapis.com/finngen-public-data-r9/summary_stats/finngen_R9_F5_AGORAPHOBIA.gz
Social phobias	2482	337577	Europe. FIN	https://storage.googleapis.com/finngen-public-data-r9/summary_stats/finngen_R9_F5_SOCPHOB.gz
Obsessive-compulsive disorder	1962	337577	Europe. FIN	https://storage.googleapis.com/finngen-public-data-r9/summary_stats/finngen_R9_F5_OCD.gz
Post-traumatic stress disorder	2282	337577	Europe. FIN	https://storage.googleapis.com/finngen-public-data-r9/summary_stats/finngen_R9_F5_PTSD.gz
Generalized anxiety disorder	4666	337577	Europe. FIN	https://storage.googleapis.com/finngen-public-data-r9/summary_stats/finngen_R9_F5_GAD.gz
Disorders of psychological development	3793	373484	Europe. FIN	https://storage.googleapis.com/finngen-public-data-r9/summary_stats/finngen_R9_F5_PSYCHDEV.gz

**Self-harm**				
Intentional self-harm by hanging	157	377120	Europe. FIN	https://storage.googleapis.com/finngen-public-data-r9/summary_stats/finngen_R9_VWXY20_INTENTI_SELF_H_HANGI_STRANGU_SUFFO.gz
Intentional self-harm by jumping from a high place	156	377121	Europe. FIN	https://storage.googleapis.com/finngen-public-data-r9/summary_stats/finngen_R9_VWXY20_INTENTI_SELF_H_JUMPI_A_HIGH_PLACE.gz
Intentional self-harm by sharp object	658	376619	Europe. FIN	https://storage.googleapis.com/finngen-public-data-r9/summary_stats/finngen_R9_VWXY20_INTENTI_SELF_H_SHARP_OBJECT.gz
Intentional self-harm by unspecified means	312	376965	Europe. FIN	https://storage.googleapis.com/finngen-public-data-r9/summary_stats/finngen_R9_VWXY20_INTENTI_SELF_H_UNSPE_MEANS.gz
Intentional self-poisoning (others)	5811	371466	Europe. FIN	https://storage.googleapis.com/finngen-public-data-r9/summary_stats/finngen_R9_VWXY20_INTENTI_SELF_P_EXPOS_OTHER_UNSPE_CHEMIC_NOXIO_SUBST.gz
Suicide or other Intentional self-harm	8978	368299	Europe. FIN	https://storage.googleapis.com/finngen-public-data-r9/summary_stats/finngen_R9_VWXY20_SUICI_OTHER_INTENTI_SELF_H.gz
**Substance-use disorders**				
Substance use	5164	337577	Europe. FIN	https://storage.googleapis.com/finngen-public-data-r9/summary_stats/finngen_R9_F5_SUBSNOALCO.gz
Alcohol abuse	4859	277526	Europe. FIN	https://storage.googleapis.com/finngen-public-data-r9/summary_stats/finngen_R9_KRA_PSY_ALCOH_EXMORE.gz
Alcohol dependence	9876	353355	Europe. FIN	https://storage.googleapis.com/finngen-public-data-r9/summary_stats/finngen_R9_F5_ALCOHOL_DEPENDENCE.gz
Alcohol use disorder	15715	361562	Europe. FIN	https://storage.googleapis.com/finngen-public-data-r9/summary_stats/finngen_R9_AUD.gz
Acute alcohol intoxication	8957	353355	Europe. FIN	https://storage.googleapis.com/finngen-public-data-r9/summary_stats/finngen_R9_F5_ALCOHOLAC.gz
Epileptic seizures related to alcohol	167	377110	Europe. FIN	https://storage.googleapis.com/finngen-public-data-r9/summary_stats/finngen_R9_EPISEIZALCO.gz
Epileptic seizures related to drugs	91	377186	Europe. FIN	https://storage.googleapis.com/finngen-public-data-r9/summary_stats/finngen_R9_EPILEPTIC_SEIZU_RELATED_DRUGS.gz
Mental and behavioural disorders due to multiple drug use	2299	370672	Europe. FIN	https://storage.googleapis.com/finngen-public-data-r9/summary_stats/finngen_R9_F5_OTHERSUB.gz
Mental and behavioural disorders due to opioids	1108	370672	Europe. FIN	https://storage.googleapis.com/finngen-public-data-r9/summary_stats/finngen_R9_F5_OPIOIDS.gz

**Impulse-control disorders**				
Habit and impulse disorders	459	366637	Europe. FIN	https://storage.googleapis.com/finngen-public-data-r9/summary_stats/finngen_R9_F5_HABIT.gz
Eating disorders				
Other eating disorders	1551	366876	Europe. FIN	https://storage.googleapis.com/finngen-public-data-r9/summary_stats/finngen_R9_F5_EATOTH.gz
Anorexia	1897	366876	Europe. FIN	https://storage.googleapis.com/finngen-public-data-r9/summary_stats/finngen_R9_R18_ANOREXIA.gz
Bulimia nervosa	1028	366876	Europe. FIN	https://storage.googleapis.com/finngen-public-data-r9/summary_stats/finngen_R9_F5_BULIMIA.gz
**Schizophrenia and other psychotic disorders**				
Schizophrenia	6515	364160	Europe. FIN	https://storage.googleapis.com/finngen-public-data-r9/summary_stats/finngen_R9_F5_SCHZPHR.gz
Schizotypal disorder	798	364160	Europe. FIN	https://storage.googleapis.com/finngen-public-data-r9/summary_stats/finngen_R9_F5_SCHIZOTYP.gz
Schizoid personality disorder	550	366637	Europe. FIN	https://storage.googleapis.com/finngen-public-data-r9/summary_stats/finngen_R9_F5_SCHIZPER.gz
Schizophrenia or delusion	13061	277526	Europe. FIN	https://storage.googleapis.com/finngen-public-data-r9/summary_stats/finngen_R9_KRA_PSY_SCHIZODEL_EXMORE.gz
Acute and transient psychotic disorders	4255	364160	Europe. FIN	https://storage.googleapis.com/finngen-public-data-r9/summary_stats/finngen_R9_F5_PSYTRANS.gz
Psychotic depression	1938	228968	Europe. FIN	https://storage.googleapis.com/finngen-public-data-r9/summary_stats/finngen_R9_F5_DEPRESSION_PSYCHOTIC.gz
Persistent delusional disorders	2418	364160	Europe. FIN	https://storage.googleapis.com/finngen-public-data-r9/summary_stats/finngen_R9_F5_DELUSIO.gz
Schizoaffective disorder	2742	364160	Europe. FIN	https://storage.googleapis.com/finngen-public-data-r9/summary_stats/finngen_R9_F5_SCHIZOAFF.gz
**Somatoform disorder**				
Somatoform disorder	5164	337577	Europe. FIN	https://storage.googleapis.com/finngen-public-data-r9/summary_stats/finngen_R9_F5_SOMATOFORM.gz
**Dementia**				
Vascular dementia	2335	360778	Europe. FIN	https://storage.googleapis.com/finngen-public-data-r9/summary_stats/finngen_R9_F5_VASCDEM.gz
Dementia	16499	356660	Europe. FIN	https://storage.googleapis.com/finngen-public-data-r9/summary_stats/finngen_R9_F5_VASCDEM.gz
Dementia in Alzheimer disease	5422	356660	Europe. FIN	https://storage.googleapis.com/finngen-public-data-r9/summary_stats/finngen_R9_F5_ALZHDEMENT.gz

Supplementary Table4 Post-hoc power calculations for Mendelian randomization analyses performed at varying causal effect sizes

Exposure	Variance explained (%)	Outcome	Sample size (proportion of cases)	Selected scenarios (OR)				
				0.9	0.8	0.7	0.6	0.5
**Morning diurnal preference**	0.0518	Mood disorders						
		Mood [affective] disorders	377277(0.1275)	1.00	1.00	1.00	1.00	1.00
		Persistent mood disorders	335948(0.0201)	0.46	0.96	1.00	1.00	1.00
		Depression	372472(0.1162)	0.99	1.00	1.00	1.00	1.00
		Depression or dysthymia	274330(0.1781)	1.00	1.00	1.00	1.00	1.00
		Bipolar affective disorders	336198(0.0208)	0.47	0.97	1.00	1.00	1.00
		Manic episode	330126(0.0028)	0.11	0.28	0.55	0.79	0.93
		Anxiety disorders						
		Phobic anxiety disorders	341880(0.0126)	0.32	0.85	0.99	1.00	1.00
		Anxiety disorders	317717(0.1265)	0.99	1.00	1.00	1.00	1.00
		Other anxiety disorders	354464(0.0476)	0.83	1.00	1.00	1.00	1.00
		Anxious personality disorder	367171(0.0015)	0.08	0.19	0.36	0.57	0.76
		Panic disorder	342378(0.014)	0.35	0.88	1.00	1.00	1.00
		Agoraphobia	338467(0.0026)	0.10	0.27	0.53	0.77	0.92
		Social phobias	340059(0.0073)	0.20	0.62	0.92	0.99	1.00
		Obsessive-compulsive disorder	339539(0.0058)	0.17	0.52	0.86	0.98	1.00
		Post-traumatic stress disorder	339859(0.0067)	0.19	0.58	0.90	0.99	1.00
		Generalized anxiety disorder	342243(0.0136)	0.34	0.87	1.00	1.00	1.00
		Disorders of psychological development	377277(0.0101)	0.29	0.80	0.99	1.00	1.00
		Self-harm						
		Intentional self-harm by hanging	377277(0.0004)	0.06	0.09	0.13	0.20	0.29
		Intentional self-harm by jumping from a high place	377277(0.0004)	0.06	0.09	0.13	0.20	0.29
		Intentional self-harm by sharp object	377277(0.0017)	0.09	0.21	0.41	0.64	0.82
		Intentional self-harm by unspecified means	377277(0.0008)	0.07	0.12	0.22	0.35	0.51
		Intentional self-poisoning (others)	377277(0.0154)	0.18	0.54	0.87	0.98	1.00
		Suicide or other Intentional self-harm	377277(0.0238)	0.57	0.99	1.00	1.00	1.00
		Substance-use disorders						
		Substance use	342741(0.0151)	0.37	0.90	1.00	1.00	1.00
		Alcohol abuse	282385(0.0172)	0.35	0.88	1.00	1.00	1.00
		Alcohol dependence	363231(0.0272)	0.61	0.99	1.00	1.00	1.00
		Alcohol use disorder	377277(0.0417)	0.80	1.00	1.00	1.00	1.00
		Acute alcohol intoxication	362312(0.0247)	0.57	0.99	1.00	1.00	1.00
		Epileptic seizures related to alcohol	377277(0.0004)	0.06	0.09	0.13	0.20	0.29
		Epileptic seizures related to drugs	377277(0.0002)	0.05	0.07	0.09	0.12	0.17
		Mental and behavioral disorders due to multiple drug use	372971(0.0062)	0.19	0.59	0.91	0.99	1.00
		Mental and behavioral disorders due to opioids	371780(0.003)	0.12	0.33	0.63	0.86	0.97
		Impulse-control disorders						
		Habit and impulse disorders	367096(0.0013)	0.08	0.17	0.32	0.51	0.70
		Eating disorders						
		Other eating disorders	368427(0.0042)	0.15	0.43	0.77	0.95	0.99
		Anorexia	368773(0.0051)	0.17	0.50	0.84	0.98	1.00
		Bulimia nervosa	367904(0.0028)	0.11	0.31	0.59	0.83	0.95

	**Schizophrenia and other psychotic disorders**						
	Schizophrenia	370675(0.0176)	0.45	0.96	1.00	1.00	1.00
	Schizotypal disorder	364958(0.0022)	0.10	0.25	0.49	0.73	0.90
	Schizoid personality disorder	367187(0.0015)	0.08	0.19	0.36	0.57	0.76
	Schizophrenia or delusion	290587(0.0449)	0.72	1.00	1.00	1.00	1.00
	Acute and transient psychotic disorders	368415(0.0115)	0.31	0.84	0.99	1.00	1.00
	Psychotic depression	230906(0.0084)	0.17	0.52	0.85	0.98	1.00
	Persistent delusional disorders	366578(0.0066)	0.20	0.61	0.92	0.99	1.00
	Schizoaffective disorder	366902(0.0075)	0.22	0.66	0.95	1.00	1.00
	**Somatoform disorder**						
	Somatoform disorder	342741(0.0151)	0.37	0.90	1.00	1.00	1.00
	**Dementia**						
	Vascular dementia	363113(0.0064)	0.19	0.59	0.91	0.99	1.00
	Dementia	373159(0.0442)	0.82	1.00	1.00	1.00	1.00
	Dementia in Alzheimer disease	362082(0.015)	0.39	0.92	1.00	1.00	1.00

Note: Variance explained, the proportion of variance in the phenotype explained by the genetic variants used in the MR analyses; Proportion of cases, cases/sample size; OR, odds ratio; Type-1 error rate of 0.05; R^2 for variance explained in the exposure.

Supplementary Table5 Post-hoc power calculations for Mendelian randomization analyses performed at varying causal effect sizes

Exposure	Variance explained (%)	Outcome	Sample size (proportion of cases)	Selected scenarios (OR)				
				0.9	0.8	0.7	0.6	0.5
**Psychiatric disorders**								
Mood [affective] disorders	0.0156	**Morning diurnal preference**	403195(0.6257)	0.98	1.00	1.00	1.00	1.00
Depression	0.0155			0.98	1.00	1.00	1.00	1.00
Depression or dysthymia	0.0201			1.00	1.00	1.00	1.00	1.00
Anxiety disorders	0.0143			0.98	1.00	1.00	1.00	1.00
Intentional self-poisoning (others)	0.0126			0.96	1.00	1.00	1.00	1.00
Alcohol dependence	0.0185			0.99	1.00	1.00	1.00	1.00
Alcohol use disorder	0.0205			1.00	1.00	1.00	1.00	1.00
Acute alcohol intoxication	0.0107			0.92	1.00	1.00	1.00	1.00
Dementia	0.1954			1.00	1.00	1.00	1.00	1.00
Dementia in Alzheimer disease	0.3459			1.00	1.00	1.00	1.00	1.00

Variance explained, the proportion of variance in the phenotype explained by the genetic variants used in the MR analyses; Proportion of cases, cases/samplesize; OR, odds ratio; Type-1 error rate of 0.05; R^2 for variance explained in the exposure.

Supplementary Table6 Potential confounders of morning person SNPs with P < 1×10-5 using the PhenoScanner database

Variables	Excluded SNP	Trait with significant associations
Morning diurnal preference	rs308521	Diastolic blood pressure
	rs1421085	Type II diabetes, self-reported diabetes, self-reported type 2 diabetes, self-reported hypertension
	rs4729854	Diabetes diagnosed by doctor, self-reported diabetes
	rs7959983	Diastolic blood pressure, self-reported hypertension
	rs9636202	Systolic blood pressure
	rs11229543	Self-reported hypertension, systolic blood pressure
	rs12140153	Diabetes diagnosed by doctor
	rs28380327	Diastolic blood pressure, mood swings
	rs28458909	Mood swings

SNP: single nucleotide polymorphism. All statistical tests were two-sided. A *P*-value < 1 ×10−5 with a potential confounder in the PhenoScanner database was considered genome-wide significant and removed.

Supplementary Table7 The association between morning diurnal preference and psychiatric disorders using MR methods

**Psychiatric disorders**	SNPs	Methods	OR (95%CI)	*P*-value
**Mood disorders**				
Mood [affective] disorders	102	MR Egger	0.95(0.76,1.18)	0.644
	102	Weighted median	0.99(0.92,1.07)	0.812
	102	Inverse variance weighted	0.94(0.87,1.01)	0.077
	102	Simple mode	1.16(0.92,1.45)	0.217
	102	Weighted mode	1.09(0.89,1.33)	0.393
Persistent mood disorders	103	MR Egger	0.73(0.46,1.15)	0.175
	103	Weighted median	0.90(0.75,1.09)	0.285
	103	Inverse variance weighted	0.90(0.78,1.04)	0.150
	103	Simple mode	1.13(0.64,1.99)	0.673
	103	Weighted mode	0.75(0.48,1.17)	0.210
Depression	103	MR Egger	0.97(0.77,1.22)	0.793
	103	Weighted median	0.98(0.91,1.07)	0.686
	103	Inverse variance weighted	0.94(0.88,1.01)	0.103
	103	Simple mode	0.97(0.77,1.22)	0.797
	103	Weighted mode	1.02(0.84,1.24)	0.826
Depression or dysthymia	103	MR Egger	0.98(0.80,1.20)	0.823
	103	Weighted median	0.95(0.88,1.02)	0.167
	103	Inverse variance weighted	0.93(0.88,1.00)	0.039
	103	Simple mode	0.94(0.77,1.15)	0.544
	103	Weighted mode	0.92(0.77,1.11)	0.403
Bipolar affective disorders	103	MR Egger	0.91(0.60,1.39)	0.672
	103	Weighted median	0.98(0.81,1.18)	0.839
	103	Inverse variance weighted	0.91(0.79,1.04)	0.158
	103	Simple mode	1.04(0.65,1.66)	0.881
	103	Weighted mode	1.01(0.65,1.57)	0.950
Manic episode	103	MR Egger	0.56(0.19,1.61)	0.284
	103	Weighted median	0.74(0.46,1.20)	0.220
	103	Inverse variance weighted	0.84(0.60,1.18)	0.320
	103	Simple mode	0.90(0.26,3.14)	0.864
	103	Weighted mode	0.67(0.24,1.87)	0.447
**Anxiety disorders**				
Phobic anxiety disorders	103	MR Egger	0.84(0.54,1.31)	0.450
	103	Weighted median	0.94(0.75,1.17)	0.567
	103	Inverse variance weighted	0.92(0.80,1.06)	0.267
	103	Simple mode	0.77(0.43,1.39)	0.392
	103	Weighted mode	0.91(0.58,1.42)	0.681
Anxiety disorders	103	MR Egger	0.97(0.78,1.21)	0.798
	103	Weighted median	0.93(0.86,1.01)	0.077
	103	Inverse variance weighted	0.90(0.84,0.96)	0.002
	103	Simple mode	0.92(0.76,1.11)	0.380
	103	Weighted mode	0.94(0.79,1.13)	0.534
Other anxiety disorders	104	MR Egger	0.98(0.74,1.31)	0.910
	104	Weighted median	0.90(0.79,1.01)	0.078
	104	Inverse variance weighted	0.90(0.82,0.99)	0.025
	104	Simple mode	0.88(0.62,1.24)	0.472
	104	Weighted mode	0.88(0.66,1.18)	0.390
Anxious personality disorder	104	MR Egger	0.94(0.28,3.22)	0.926
	104	Weighted median	0.90(0.50,1.61)	0.720
	104	Inverse variance weighted	0.96(0.65,1.42)	0.836
	104	Simple mode	0.80(0.19,3.37)	0.767
	104	Weighted mode	0.83(0.22,3.11)	0.781
Panic disorder	103	MR Egger	0.82(0.54,1.24)	0.357
	103	Weighted median	0.90(0.74,1.10)	0.297
	103	Inverse variance weighted	0.91(0.80,1.04)	0.188
	103	Simple mode	0.81(0.49,1.35)	0.423
	103	Weighted mode	0.83(0.52,1.33)	0.435
Agoraphobia	103	MR Egger	0.36(0.13,1.01)	0.054
	103	Weighted median	0.63(0.40,1.00)	0.051
	103	Inverse variance weighted	0.79(0.56,1.10)	0.163
	103	Simple mode	0.73(0.25,2.10)	0.557
	103	Weighted mode	0.60(0.25,1.47)	0.270

	103	MR Egger	0.77(0.43,1.36)	0.369
	103	Weighted median	1.12(0.86,1.48)	0.401
Social phobias	103	Inverse variance weighted	0.92(0.77,1.11)	0.399
	103	Simple mode	1.47(0.72,3.00)	0.298
	103	Weighted mode	1.36(0.74,2.49)	0.327
	104	MR Egger	1.23(0.59,2.59)	0.578
	104	Weighted median	0.92(0.67,1.24)	0.573
Obsessive-compulsive disorder	104	Inverse variance weighted	0.94(0.74,1.20)	0.626
	104	Simple mode	0.76(0.29,1.98)	0.579
	104	Weighted mode	0.71(0.27,1.86)	0.488
	103	MR Egger	0.85(0.46,1.55)	0.590
	103	Weighted median	0.82(0.62,1.10)	0.183
Post-traumatic stress disorder	103	Inverse variance weighted	0.89(0.73,1.08)	0.221
	103	Simple mode	1.01(0.45,2.29)	0.984
	103	Weighted mode	0.89(0.45,1.73)	0.726
	104	MR Egger	1.11(0.69,1.78)	0.668
	104	Weighted median	0.97(0.78,1.19)	0.762
Generalized anxiety disorder	104	Inverse variance weighted	0.94(0.81,1.09)	0.403
	104	Simple mode	1.15(0.69,1.91)	0.604
	104	Weighted mode	1.07(0.68,1.66)	0.777
	103	MR Egger	1.19(0.74,1.92)	0.466
	103	Weighted median	1.07(0.85,1.34)	0.562
Disorders of psychological development	103	Inverse variance weighted	1.03(0.88,1.20)	0.701
	103	Simple mode	1.08(0.60,1.94)	0.802
	103	Weighted mode	1.20(0.72,1.99)	0.490
**Self-harm**				
	104	MR Egger	0.71(0.07,6.76)	0.768
	104	Weighted median	1.10(0.37,3.29)	0.863
Intentional self-harm by hanging	104	Inverse variance weighted	0.94(0.45,1.94)	0.866
	104	Simple mode	0.88(0.04,17.73)	0.933
	104	Weighted mode	1.13(0.08,16.80)	0.932
	103	MR Egger	0.06(0.01,0.66)	0.024
	103	Weighted median	0.48(0.15,1.53)	0.218
Intentional self-harm by jumping from a high place	103	Inverse variance weighted	0.53(0.24,1.17)	0.116
	103	Simple mode	0.6(0.03,11.77)	0.738
	103	Weighted mode	0.32(0.03,3.68)	0.360
	103	MR Egger	1.09(0.36,3.31)	0.886
	103	Weighted median	0.95(0.56,1.59)	0.835
Intentional self-harm by sharp object	103	Inverse variance weighted	0.79(0.55,1.13)	0.201
	103	Simple mode	1.73(0.43,6.95)	0.442
	103	Weighted mode	1.17(0.39,3.53)	0.786
	104	MR Egger	0.21(0.04,1.08)	0.065
	104	Weighted median	0.64(0.30,1.39)	0.261
Intentional self-harm by unspecified means	104	Inverse variance weighted	0.82(0.48,1.38)	0.451
	104	Simple mode	0.37(0.06,2.44)	0.307
	104	Weighted mode	0.41(0.08,1.98)	0.269
	103	MR Egger	0.67(0.44,1.01)	0.059
	103	Weighted median	0.91(0.75,1.10)	0.327
Intentional self-poisoning (others)	103	Inverse variance weighted	0.88(0.77,1.00)	0.058
	103	Simple mode	0.90(0.54,1.51)	0.686
	103	Weighted mode	0.90(0.59,1.38)	0.626
	103	MR Egger	0.60(0.41,0.89)	0.013
	103	Weighted median	0.86(0.74,1.01)	0.073
Suicide or other Intentional self-harm	103	Inverse variance weighted	0.87(0.76,0.99)	0.033
	103	Simple mode	0.97(0.61,1.56)	0.911
	103	Weighted mode	0.92(0.63,1.34)	0.654
**Substance-use disorders**				
	104	MR Egger	0.80(0.52,1.23)	0.320
	104	Weighted median	0.88(0.73,1.05)	0.159
Substance use	104	Inverse variance weighted	0.81(0.71,0.93)	0.002
	104	Simple mode	1.08(0.65,1.80)	0.764
	104	Weighted mode	1.12(0.68,1.85)	0.649
	104	MR Egger	0.83(0.50,1.40)	0.491
	104	Weighted median	0.99(0.80,1.22)	0.919
Alcohol abuse	104	Inverse variance weighted	0.86(0.73,1.02)	0.084
	104	Simple mode	1.12(0.60,2.08)	0.715
	104	Weighted mode	1.14(0.68,1.90)	0.624

Alcohol dependence	103	MR Egger	0.73(0.51,1.05)	0.091
	103	Weighted median	0.86(0.73,1.00)	0.056
	103	Inverse variance weighted	0.82(0.73,0.92)	0.001
	103	Simple mode	0.95(0.61,1.48)	0.817
	103	Weighted mode	0.89(0.65,1.23)	0.482
Alcohol use disorder	103	MR Egger	0.76(0.55,1.04)	0.086
	103	Weighted median	0.84(0.74,0.95)	0.005
	103	Inverse variance weighted	0.85(0.76,0.94)	0.001
	103	Simple mode	0.85(0.60,1.21)	0.370
	103	Weighted mode	0.82(0.61,1.10)	0.195
Acute alcohol intoxication	103	MR Egger	0.82(0.57,1.18)	0.286
	103	Weighted median	0.89(0.77,1.04)	0.144
	103	Inverse variance weighted	0.86(0.76,0.96)	0.010
	103	Simple mode	0.83(0.53,1.30)	0.411
	103	Weighted mode	1.13(0.79,1.62)	0.515
Epileptic seizures related to alcohol	104	MR Egger	0.28(0.03,2.52)	0.259
	104	Weighted median	1.72(0.62,4.78)	0.299
	104	Inverse variance weighted	1.47(0.72,3.00)	0.285
	104	Simple mode	2.59(0.22,30.00)	0.447
	104	Weighted mode	2.33(0.28,19.12)	0.434
Epileptic seizures related to drugs	104	MR Egger	2.99(0.14,65.85)	0.490
	104	Weighted median	0.76(0.17,3.37)	0.722
	104	Inverse variance weighted	0.47(0.17,1.27)	0.137
	104	Simple mode	1.33(0.03,54.82)	0.880
	104	Weighted mode	1.68(0.06,50.22)	0.766
Mental and behavioural disorders due to multiple drug use	104	MR Egger	0.66(0.34,1.28)	0.218
	104	Weighted median	0.96(0.73,1.28)	0.801
	104	Inverse variance weighted	0.92(0.74,1.14)	0.445
	104	Simple mode	1.22(0.57,2.59)	0.608
	104	Weighted mode	1.00(0.53,1.87)	0.991
Mental and behavioural disorders due to opioids	103	MR Egger	0.55(0.20,1.50)	0.246
	103	Weighted median	0.86(0.55,1.34)	0.501
	103	Inverse variance weighted	0.80(0.58,1.10)	0.167
	103	Simple mode	0.75(0.25,2.24)	0.602
	103	Weighted mode	0.76(0.31,1.90)	0.563
**Impulse-control disorders**				
Habit and impulse disorders	104	MR Egger	0.62(0.16,2.47)	0.500
	104	Weighted median	0.84(0.45,1.59)	0.595
	104	Inverse variance weighted	0.87(0.56,1.36)	0.536
	104	Simple mode	0.49(0.11,2.12)	0.342
	104	Weighted mode	0.62(0.17,2.22)	0.465
**Eating disorders**				
Other eating disorders	103	MR Egger	0.86(0.42,1.78)	0.684
	103	Weighted median	0.89(0.64,1.25)	0.517
	103	Inverse variance weighted	0.98(0.77,1.24)	0.843
	103	Simple mode	0.76(0.36,1.63)	0.487
	103	Weighted mode	0.76(0.36,1.61)	0.477
Anorexia	103	MR Egger	0.80(0.41,1.54)	0.505
	103	Weighted median	1.01(0.73,1.38)	0.972
	103	Inverse variance weighted	0.99(0.80,1.23)	0.931
	103	Simple mode	0.97(0.42,2.27)	0.945
	103	Weighted mode	0.94(0.44,2.01)	0.868
Bulimia nervosa (incl. atypical)	104	MR Egger	1.16(0.48,2.84)	0.739
	104	Weighted median	1.01(0.67,1.53)	0.947
	104	Inverse variance weighted	0.96(0.72,1.28)	0.786
	104	Simple mode	2.06(0.63,6.75)	0.235
	104	Weighted mode	0.94(0.34,2.65)	0.909
**Schizophrenia and other psychotic disorders**				
Schizophrenia	103	MR Egger	0.58(0.34,0.99)	0.048
	103	Weighted median	0.73(0.57,0.93)	0.010
	103	Inverse variance weighted	0.77(0.65,0.92)	0.003
	103	Simple mode	0.52(0.27,1.00)	0.052
	103	Weighted mode	0.50(0.26,0.95)	0.036

	104	MR Egger	0.73(0.22,2.45)	0.616
	104	Weighted median	1.02(0.59,1.77)	0.942
Schizotypal disorder	104	Inverse variance weighted	0.93(0.63,1.37)	0.710
	104	Simple mode	1.41(0.34,5.90)	0.638
	104	Weighted mode	1.45(0.37,5.77)	0.595
	103	MR Egger	0.47(0.14,1.60)	0.230
	103	Weighted median	0.92(0.52,1.61)	0.764
Schizoid personality disorder	103	Inverse variance weighted	1.05(0.71,1.56)	0.809
	103	Simple mode	0.71(0.20,2.50)	0.595
	103	Weighted mode	0.67(0.21,2.17)	0.507
	103	MR Egger	0.73(0.48,1.11)	0.144
	103	Weighted median	0.72(0.60,0.86)	0.000
Schizophrenia or delusion	103	Inverse variance weighted	0.80(0.70,0.92)	0.001
	103	Simple mode	0.56(0.32,0.96)	0.036
	103	Weighted mode	0.54(0.32,0.93)	0.029
	103	MR Egger	0.67(0.38,1.17)	0.159
	103	Weighted median	0.95(0.74,1.22)	0.709
Acute and transient psychotic disorders	103	Inverse variance weighted	0.94(0.79,1.13)	0.532
	103	Simple mode	1.14(0.55,2.36)	0.728
	103	Weighted mode	0.94(0.51,1.75)	0.852
	104	MR Egger	1.93(0.95,3.94)	0.073
	104	Weighted median	1.38(0.98,1.93)	0.063
Psychotic depression	104	Inverse variance weighted	1.06(0.84,1.34)	0.618
	104	Simple mode	1.81(0.80,4.07)	0.157
	104	Weighted mode	1.65(0.85,3.18)	0.141
	103	MR Egger	0.62(0.32,1.19)	0.152
	103	Weighted median	1.02(0.75,1.37)	0.923
Persistent delusional disorders	103	Inverse variance weighted	0.99(0.80,1.22)	0.894
	103	Simple mode	1.18(0.51,2.74)	0.696
	103	Weighted mode	1.22(0.57,2.64)	0.611
	104	MR Egger	0.69(0.34,1.42)	0.319
	104	Weighted median	0.96(0.69,1.32)	0.785
Schizoaffective disorder	104	Inverse variance weighted	0.96(0.77,1.21)	0.759
	104	Simple mode	0.99(0.44,2.22)	0.984
	104	Weighted mode	0.97(0.48,1.98)	0.941
**Somatoform disorder**				
	104	MR Egger	0.83(0.56,1.23)	0.358
	104	Weighted median	0.89(0.74,1.07)	0.226
Somatoform disorder	104	Inverse variance weighted	0.90(0.79,1.02)	0.095
	104	Simple mode	0.92(0.58,1.45)	0.721
	104	Weighted mode	0.91(0.60,1.38)	0.660
**Dementia**				
	104	MR Egger	0.87(0.48,1.57)	0.639
	104	Weighted median	0.89(0.68,1.17)	0.415
Vascular dementia	104	Inverse variance weighted	0.93(0.77,1.13)	0.454
	104	Simple mode	0.98(0.47,2.03)	0.950
	104	Weighted mode	0.93(0.48,1.82)	0.839
	103	MR Egger	1.17(0.88,1.56)	0.272
	103	Weighted median	1.01(0.89,1.14)	0.860
Dementia	103	Inverse variance weighted	0.98(0.90,1.08)	0.737
	103	Simple mode	0.99(0.73,1.33)	0.926
	103	Weighted mode	1.05(0.83,1.32)	0.702
	103	MR Egger	1.16(0.71,1.91)	0.556
	103	Weighted median	0.91(0.74,1.12)	0.380
Dementia in Alzheimer disease	103	Inverse variance weighted	0.93(0.79,1.09)	0.349
	103	Simple mode	0.62(0.33,1.17)	0.144
	103	Weighted mode	0.73(0.41,1.30)	0.290

Supplementary Table8 Results of MR Setiger direction test for morning diurnal preference on psychiatric disorders

Outcomes	R^2 exposure	R^2 outcome	Correct causal direction	Setiger *P*-value
**Mood disorders**				
Mood [affective] disorders	1.18E-02	6.69E-04	TRUE	1.04E-293
Persistent mood disorders	1.18E-02	4.75E-04	TRUE	0.00E+00
Depression	1.18E-02	6.33E-04	TRUE	1.21E-298
Depression or dysthymia	1.18E-02	5.47E-04	TRUE	0.00E+00
Bipolar affective disorders	1.18E-02	3.69E-04	TRUE	0.00E+00
Manic episode	1.18E-02	3.45E-04	TRUE	0.00E+00
**Anxiety disorders**				
Phobic anxiety disorders	1.18E-02	2.78E-04	TRUE	0.00E+00
Anxiety disorders	1.18E-02	5.57E-04	TRUE	0.00E+00
Other anxiety disorders	1.18E-02	4.70E-04	TRUE	0.00E+00
Anxious personality disorder	1.18E-02	2.61E-04	TRUE	0.00E+00
Panic disorder	1.18E-02	2.65E-04	TRUE	0.00E+00
Agoraphobia	1.18E-02	3.34E-04	TRUE	0.00E+00
Social phobias	1.18E-02	2.34E-04	TRUE	0.00E+00
Obsessive-compulsive disorder	1.18E-02	3.61E-04	TRUE	0.00E+00
Post-traumatic stress disorder	1.18E-02	3.13E-04	TRUE	0.00E+00
Generalized anxiety disorder	1.18E-02	3.52E-04	TRUE	0.00E+00
Disorders of psychological development	1.18E-02	2.61E-04	TRUE	0.00E+00
**Self-harm**				
Intentional self-harm by hanging	1.18E-02	2.75E-04	TRUE	0.00E+00
Intentional self-harm by jumping from a high place	1.18E-02	3.48E-04	TRUE	0.00E+00
Intentional self-harm by sharp object	1.18E-02	2.61E-04	TRUE	0.00E+00
Intentional self-harm by unspecified means	1.18E-02	2.92E-04	TRUE	0.00E+00
Intentional self-poisoning (others)	1.18E-02	3.41E-04	TRUE	0.00E+00
Suicide or other Intentional self-harm	1.18E-02	4.67E-04	TRUE	0.00E+00
**Substance-use disorders**				
Substance use	1.18E-02	4.22E-04	TRUE	0.00E+00
Alcohol abuse	1.18E-02	4.08E-04	TRUE	0.00E+00
Alcohol dependence	1.18E-02	4.56E-04	TRUE	0.00E+00
Alcohol use disorder	1.18E-02	5.48E-04	TRUE	0.00E+00
Acute alcohol intoxication	1.18E-02	4.03E-04	TRUE	0.00E+00
Epileptic seizures related to alcohol	1.18E-02	2.88E-04	TRUE	0.00E+00
Epileptic seizures related to drugs	1.18E-02	3.10E-04	TRUE	0.00E+00
Mental and behavioural disorders due to multiple drug use	1.18E-02	3.32E-04	TRUE	0.00E+00
Mental and behavioural disorders due to opioids	1.18E-02	3.88E-04	TRUE	0.00E+00
**Impulse-control disorders**				
Habit and impulse disorders	1.18E-02	3.04E-04	TRUE	0.00E+00
**Eating disorders**				
Other eating disorders	1.18E-02	2.46E-04	TRUE	0.00E+00
Anorexia	1.18E-02	2.82E-04	TRUE	0.00E+00
Bulimia nervosa	1.18E-02	2.76E-04	TRUE	0.00E+00
**Schizophrenia and other psychotic disorders**				
Schizophrenia	1.18E-02	3.18E-04	TRUE	0.00E+00
Schizotypal disorder	1.18E-02	3.13E-04	TRUE	0.00E+00
Schizoid personality disorder	1.18E-02	1.96E-04	TRUE	0.00E+00
Schizophrenia or delusion	1.18E-02	4.02E-04	TRUE	0.00E+00

Acute and transient psychotic disorders	1.18E-02	3.09E-04	TRUE	0.00E+00
Psychotic depression	1.18E-02	3.10E-04	TRUE	0.00E+00
Persistent delusional disorders	1.18E-02	2.95E-04	TRUE	0.00E+00
Schizoaffective disorder	1.18E-02	2.69E-04	TRUE	0.00E+00
**Somatoform disorder**				
Somatoform disorder	1.18E-02	2.88E-04	TRUE	0.00E+00
**Dementia**				
Vascular dementia	1.18E-02	2.61E-04	TRUE	0.00E+00
Dementia	1.18E-02	3.87E-04	TRUE	0.00E+00
Dementia in Alzheimer disease	1.18E-02	4.24E-04	TRUE	0.00E+00

Supplementary Table9 Inspected the horizontal pleiotropy through its intercept and provides estimates after correcting for the pleiotropic effects in the MR- Egger regression

Exposure	Outcome	Estimates	Intercept	*P*-value
Morning diurnal preference	**Mood disorders**			
	Mood [affective] disorders	0.004	0.000	0.909
	Persistent mood disorders	0.008	0.008	0.344
	Depression	0.004	-0.001	0.783
	Depression or dysthymia	0.004	-0.002	0.642
	Bipolar affective disorders	0.008	0.000	0.974
	Manic episode	0.020	0.016	0.426
	**Anxiety disorders**			
	Phobic anxiety disorders	0.008	0.003	0.673
	Anxiety disorders	0.004	-0.003	0.442
	Other anxiety disorders	0.005	-0.003	0.525
	Anxious personality disorder	0.023	0.001	0.978
	Panic disorder	0.008	0.004	0.599
	Agoraphobia	0.019	0.030	0.117
	Social phobias	0.011	0.007	0.507
	Obsessive-compulsive disorder	0.014	-0.010	0.453
	Post-traumatic stress disorder	0.011	0.002	0.877
	Generalized anxiety disorder	0.009	-0.006	0.462
	Disorders of psychological development	0.009	-0.006	0.523
	**Self-harm**			
	Intentional self-harm by hanging	0.041	0.011	0.799
	Intentional self-harm by jumping from a high place	0.045	0.084	0.063
	Intentional self-harm by sharp object	0.020	-0.012	0.557
	Intentional self-harm by unspecified means	0.030	0.051	0.089
	Intentional self-poisoning (others)	0.008	0.011	0.174
	Suicide or other Intentional self-harm	0.007	0.014	0.056
	**Substance-use disorders**			
	Substance use	0.008	0.000	0.977
	Alcohol abuse	0.009	0.001	0.886
	Alcohol dependence	0.007	0.004	0.506
	Alcohol use disorder	0.006	0.004	0.458
	Acute alcohol intoxication	0.007	0.002	0.800
	Epileptic seizures related to alcohol	0.040	0.063	0.121
	Epileptic seizures related to drugs	0.057	-0.071	0.217
	Mental and behavioural disorders due to multiple drug use	0.012	0.013	0.297
	Mental and behavioural disorders due to opioids	0.018	0.014	0.448
	**Impulse-control disorders**			
	Habit and impulse disorders	0.025	0.013	0.614
	**Eating disorders**			
	Other eating disorders	0.013	0.005	0.717
	Anorexia	0.012	0.008	0.500
	Bulimia nervosa	0.016	-0.007	0.657
	**Schizophrenia and other psychotic disorders**			
	Schizophrenia	0.010	0.011	0.272
	Schizotypal disorder	0.022	0.009	0.686
	Schizoid personality disorder	0.022	0.031	0.177
	Schizophrenia or delusion	0.008	0.004	0.646
	Acute and transient psychotic disorders	0.010	0.013	0.201
	Psychotic depression	0.013	-0.023	0.085
	Persistent delusional disorders	0.012	0.018	0.142
	Schizoaffective disorder	0.013	0.012	0.343
	**Somatoform disorder**			
	Somatoform disorder	0.007	0.003	0.684
	**Dementia**			
	Vascular dementia	0.011	0.003	0.810
	Dementia	0.005	-0.007	0.202
	Dementia in Alzheimer disease	0.009	-0.009	0.346

Supplementary Table10 Verified the heterogeneity between the causal estimates of each SNPs in the IVW and MR-Egger methods

Exposure	Outcome	Methods	Q statistic	*P*-value
Morning diurnal preference	**Mood disorders**			
	Mood [affective] disorders	MR Egger	241.679	<0.001
		Inverse variance weighted	241.711	<0.001
	Persistent mood disorders	MR Egger	169.277	<0.001
		Inverse variance weighted	170.790	<0.001
	Depression	MR Egger	232.179	<0.001
		Inverse variance weighted	232.355	<0.001
	Depression or dysthymia	MR Egger	197.149	<0.001
		Inverse variance weighted	197.573	<0.001
	Bipolar affective disorders	MR Egger	130.169	0.027
		Inverse variance weighted	130.171	0.031
	Manic episode	MR Egger	123.649	0.063
		Inverse variance weighted	124.430	0.065
	**Anxiety disorders**			
	Phobic anxiety disorders	MR Egger	102.688	0.434
		Inverse variance weighted	102.870	0.457
	Anxiety disorders	MR Egger	185.394	<0.001
		Inverse variance weighted	186.490	<0.001
	Other anxiety disorders	MR Egger	162.131	<0.001
		Inverse variance weighted	162.778	<0.001
	Anxious personality disorder	MR Egger	95.094	0.673
		Inverse variance weighted	95.095	0.698
	Panic disorder	MR Egger	93.801	0.681
		Inverse variance weighted	94.079	0.699
	Agoraphobia	MR Egger	118.707	0.110
		Inverse variance weighted	121.645	0.090
	Social phobias	MR Egger	84.151	0.887
		Inverse variance weighted	84.594	0.894
	Obsessive-compulsive disorder	MR Egger	131.642	0.026
		Inverse variance weighted	132.375	0.027
	Post-traumatic stress disorder	MR Egger	101.878	0.457
		Inverse variance weighted	101.902	0.484
	Generalized anxiety disorder	MR Egger	127.776	0.043
		Inverse variance weighted	128.460	0.045
	Disorders of psychological development	MR Egger	96.909	0.597
		Inverse variance weighted	97.321	0.613
	**Self-harm**			
	Intentional self-harm by hanging	MR Egger	101.331	0.500
		Inverse variance weighted	101.396	0.526
	Intentional self-harm by jumping from a high place	MR Egger	117.986	0.119
		Inverse variance weighted	122.124	0.085
	Intentional self-harm by sharp object	MR Egger	89.608	0.784
		Inverse variance weighted	89.955	0.797
	Intentional self-harm by unspecified means	MR Egger	103.850	0.430
		Inverse variance weighted	106.845	0.378
	Intentional self-poisoning (others)	MR Egger	120.639	0.089
		Inverse variance weighted	122.883	0.078
	Suicide or other Intentional self-harm	MR Egger	161.394	<0.001
		Inverse variance weighted	167.361	<0.001
	**Substance-use disorders**			
	Substance use	MR Egger	143.733	0.004
		Inverse variance weighted	143.734	0.005
	Alcohol abuse	MR Egger	145.300	0.003
		Inverse variance weighted	145.330	0.004
	Alcohol dependence	MR Egger	152.385	0.001
		Inverse variance weighted	153.058	0.001
	Alcohol use disorder	MR Egger	184.231	<0.001
		Inverse variance weighted	185.246	<0.001
	Acute alcohol intoxication	MR Egger	140.924	0.005
		Inverse variance weighted	141.014	0.006

Epileptic seizures related to alcohol	MR Egger	102.558	0.466
	Inverse variance weighted	105.016	0.426
Epileptic seizures related to drugs	MR Egger	111.264	0.249
	Inverse variance weighted	112.948	0.236
Mental and behavioural disorders due to multiple drug use	MR Egger	121.514	0.091
	Inverse variance weighted	122.824	0.089
Mental and behavioural disorders due to opioids	MR Egger	136.725	0.010
	Inverse variance weighted	137.510	0.011
**Impulse-control disorders**			
Habit and impulse disorders	MR Egger	111.340	0.248
	Inverse variance weighted	111.618	0.264
**Eating disorders**			
Other eating disorders	MR Egger	76.871	0.965
	Inverse variance weighted	77.003	0.969
Anorexia	MR Egger	103.454	0.414
	Inverse variance weighted	103.924	0.428
Bulimia nervosa	MR Egger	102.582	0.465
	Inverse variance weighted	102.781	0.488
**Schizophrenia and other psychotic disorders**			
Schizophrenia	MR Egger	104.540	0.385
	Inverse variance weighted	105.804	0.378
Schizotypal disorder	MR Egger	117.514	0.140
	Inverse variance weighted	117.704	0.153
Schizoid personality disorder	MR Egger	69.345	0.993
	Inverse variance weighted	71.197	0.991
Schizophrenia or delusion	MR Egger	133.326	0.017
	Inverse variance weighted	133.606	0.020
Acute and transient psychotic disorders	MR Egger	114.051	0.177
	Inverse variance weighted	115.919	0.164
Psychotic depression	MR Egger	111.168	0.251
	Inverse variance weighted	114.472	0.207
Persistent delusional disorders	MR Egger	107.132	0.319
	Inverse variance weighted	109.450	0.289
Schizoaffective disorder	MR Egger	99.027	0.565
	Inverse variance weighted	99.934	0.567
**Somatoform disorder**			
Somatoform disorder	MR Egger	102.309	0.473
	Inverse variance weighted	102.476	0.496
**Dementia**			
Vascular dementia	MR Egger	95.392	0.665
	Inverse variance weighted	95.451	0.689
Dementia	MR Egger	134.131	0.015
	Inverse variance weighted	136.321	0.013
Dementia in Alzheimer disease	MR Egger	152.837	0.001
	Inverse variance weighted	154.196	0.001

Supplementary Table11 Identify outlier SNPs using outlier (MR-PRESSO) test in IVW methods

Exposure	Outcome	SNPs	OR (95%CI)	*P*-value
Morning diurnal preference	**Mood disorders**			
	Mood [affective] disorders	95	0.95(0.9,1.01)	0.108
	Persistent mood disorders	98	0.88(0.77,1.01)	0.065
	Depression	97	0.95(0.89,1.01)	0.106
	Depression or dysthymia	95	0.97(0.91,1.02)	0.208
	Bipolar affective disorders	100	0.92(0.82,1.05)	0.215
	**Anxiety disorders**			
	Anxiety disorders	96	0.92(0.87,0.97)	0.004
	Other anxiety disorders	102	0.92(0.84,1.01)	0.070
	Obsessive-compulsive disorder	102	1(0.8,1.25)	0.970
	Generalized anxiety disorder	103	0.92(0.79,1.07)	0.265
	**Self-harm**			
	Suicide or other Intentional self-harm	98	0.89(0.79,0.99)	0.037
	**Substance-use disorders**			
	Substance use	102	0.8(0.71,0.91)	0.001
	Alcohol abuse	103	0.88(0.75,1.04)	0.125
	Alcohol dependence	100	0.84(0.75,0.93)	0.002
	Alcohol use disorder	97	0.83(0.76,0.91)	0.000
	Acute alcohol intoxication	101	0.88(0.79,0.98)	0.026
	Mental and behavioural disorders due to opioids	101	0.8(0.59,1.09)	0.164
	**Schizophrenia and other psychotic disorders**			
	Schizophrenia or delusion	100	0.79(0.69,0.89)	<0.001
	**Dementia**			
	Dementia	100	0.99(0.91,1.08)	0.893
	Dementia in Alzheimer disease	99	0.97(0.84,1.13)	0.711

Supplementary Table12 The association between psychiatric disorders and morning diurnal preference using MR methods

Exposure	Outcome	SNPs	Methods	OR (95%CI)	*P*-value
Mood [affective] disorders	Morning diurnal preference	16	MR Egger	0.95(0.68,1.33)	0.768
		16	Weighted median	1.01(0.94,1.09)	0.699
		16	Inverse variance weighted	1.04(0.98,1.11)	0.194
		16	Simple mode	0.97(0.85,1.12)	0.703
		16	Weighted mode	0.99(0.87,1.12)	0.834
Depression		13	MR Egger	1.43(0.49,4.18)	0.527
		13	Weighted median	1.05(0.98,1.14)	0.185
		13	Inverse variance weighted	1.00(0.92,1.09)	0.922
		13	Simple mode	1.07(0.95,1.22)	0.285
		13	Weighted mode	1.07(0.95,1.20)	0.274
Depression or dysthymia		18	MR Egger	0.86(0.40,1.85)	0.699
		18	Weighted median	1.01(0.94,1.09)	0.688
		18	Inverse variance weighted	0.99(0.91,1.08)	0.873
		18	Simple mode	1.02(0.92,1.14)	0.660
		18	Weighted mode	1.02(0.93,1.14)	0.643
Anxiety disorders		13	MR Egger	1.16(0.80,1.68)	0.457
		13	Weighted median	1.02(0.94,1.12)	0.593
		13	Inverse variance weighted	0.99(0.91,1.07)	0.788
		13	Simple mode	1.05(0.90,1.21)	0.561
		13	Weighted mode	1.04(0.92,1.19)	0.528
Intentional self-poisoning (others)		2	Inverse variance weighted	0.99(0.89,1.09)	0.813
Alcohol dependence		3	MR Egger	0.98(0.95,1.02)	0.550
		3	Weighted median	0.97(0.94,1.00)	0.040
		3	Inverse variance weighted	0.97(0.94,1.00)	0.045
		3	Simple mode	0.92(0.85,1.00)	0.184
		3	Weighted mode	0.98(0.95,1.01)	0.292
Alcohol use disorder		6	MR Egger	0.98(0.95,1.01)	0.316
		6	Weighted median	0.98(0.95,1.01)	0.116
		6	Inverse variance weighted	0.96(0.94,0.99)	0.011
		6	Simple mode	0.90(0.84,0.98)	0.053
		6	Weighted mode	0.98(0.95,1.01)	0.199
Acute alcohol intoxication		2	Inverse variance weighted	0.97(0.93,1.01)	0.112
Dementia		12	MR Egger	1.04(1.00,1.08)	0.067
		12	Weighted median	1.03(1.01,1.05)	0.004
		12	Inverse variance weighted	1.01(0.98,1.04)	0.444
		12	Simple mode	1.00(0.93,1.08)	0.942
		12	Weighted mode	1.03(1.01,1.05)	0.007
Dementia in Alzheimer disease		7	MR Egger	1.02(0.99,1.05)	0.269
		7	Weighted median	1.02(1.01,1.03)	0.001
		7	Inverse variance weighted	1.02(1.00,1.04)	0.062
		7	Simple mode	1.03(0.98,1.08)	0.283
		7	Weighted mode	1.02(1.01,1.04)	0.013

Supplementary Table13 Results of MR Setiger direction test for psychiatric disorders on morning diurnal preference

Exposure	Outcomes	R^2 exposure	R^2 outcome	Correct causal direction	Setiger *P*-value
Mood [affective] disorders	Morning diurnal preference	0.001605698	9.91E-05	TRUE	2.16E-40
Depression		0.001263429	7.68E-05	TRUE	2.76E-32
Depression or dysthymia		0.001782419	0.000138343	TRUE	2.81E-41
Anxiety disorders		0.001165813	6.00E-05	TRUE	2.04E-31
Intentional self-poisoning (others)		0.000172255	7.70E-06	TRUE	4.88E-06
Alcohol dependence		0.000353494	1.90E-05	TRUE	1.83E-10
Alcohol use disorder		0.000653196	2.61E-05	TRUE	1.73E-19
Acute alcohol intoxication		0.000170464	1.35E-05	TRUE	3.41E-05
Dementia		0.001379527	0.000100473	TRUE	4.55E-33
Dementia in Alzheimer disease		0.001216616	7.17E-05	TRUE	1.87E-31

Supplementary Table14 Inspected the horizontal pleiotropy through its intercept and provides estimates after correcting for the pleiotropic effects in the MR- Egger regression

Exposure	Outcome	Estimates	Intercept	*P*-value
Mood [affective] disorders	Morning diurnal preference	0.008	0.005	0.591
Depression		0.027	-0.018	0.531
Depression or dysthymia		0.019	0.007	0.710
Anxiety disorders		0.010	-0.009	0.412
Intentional self-poisoning (others)		NA	NA	NA
Alcohol dependence		0.005	-0.007	0.372
Alcohol use disorder		0.003	-0.004	0.236
Acute alcohol intoxication		NA	NA	NA
Dementia		0.004	-0.008	0.068
Dementia in Alzheimer disease		0.006	0.000	0.944

Supplementary Table15 Verified the heterogeneity between the causal estimates of each SNPs in the IVW and MR- Egger methods

Exposure	Outcome	Methods	Q statistic	*P*-value
Mood [affective] disorders	Morning diurnal preference	MR Egger	20.787	0.107
		Inverse variance weighted	21.237	0.129
Depression		MR Egger	29.289	0.002
		Inverse variance weighted	30.404	0.002
Depression or dysthymia		MR Egger	54.822	0.000
		Inverse variance weighted	55.313	0.000
Anxiety disorders		MR Egger	22.441	0.021
		Inverse variance weighted	23.922	0.021
Intentional self-poisoning (others)		Inverse variance weighted	2.833	0.092
		MR Egger	0.266	0.606
Alcohol dependence		Inverse variance weighted	2.558	0.278
		MR Egger	2.039	0.729
Alcohol use disorder		Inverse variance weighted	3.976	0.553
		Inverse variance weighted	1.452	0.228
Dementia		MR Egger	27.627	0.002
		Inverse variance weighted	39.205	0.000
Dementia in Alzheimer disease		MR Egger	18.835	0.002
		Inverse variance weighted	18.856	0.004

Supplementary Table16 Identify outlier SNPs using outlier (MR-PRESSO) test in MR methods

Exposure	Outcome	Methods	SNPs	OR (95%CI)	P-value
Depression	Morning diurnal preference	MR Egger	12	1.23(0.63,2.38)	0.557
		Weighted median	12	1.06(0.98,1.14)	0.126
		Inverse variance weighted	12	1.04(0.99,1.11)	0.130
		Simple mode	12	1.07(0.94,1.23)	0.323
		Weighted mode	12	1.07(0.94,1.22)	0.334
Depression or dysthymia		MR Egger	16	0.88(0.53,1.46)	0.629
		Weighted median	16	1.02(0.95,1.09)	0.667
		Inverse variance weighted	16	1.00(0.94,1.06)	0.982
		Simple mode	16	1.02(0.91,1.15)	0.695
		Weighted mode	16	1.02(0.91,1.15)	0.701
